# Repurposing a Fully Reducing Polyketide Synthase toward 2-Methyl Guerbet-like Lipids

**DOI:** 10.1021/acscatal.4c04714

**Published:** 2024-10-31

**Authors:** Michael A. Herrera, Stephen McColm, Louise-Marie Craigie, Joanna Simpson, Fraser Brown, David J. Clarke, Reuben Carr, Dominic J. Campopiano

**Affiliations:** 1 School of Chemistry, The University of Edinburgh, Edinburgh EH9 3FJ, U.K.; 2 Ingenza Ltd., Roslin Innovation Centre, Edinburgh EH25 9RG, U.K.

**Keywords:** biocatalysis, biosynthesis, polyketides, lipids, PKS, Guerbet

## Abstract

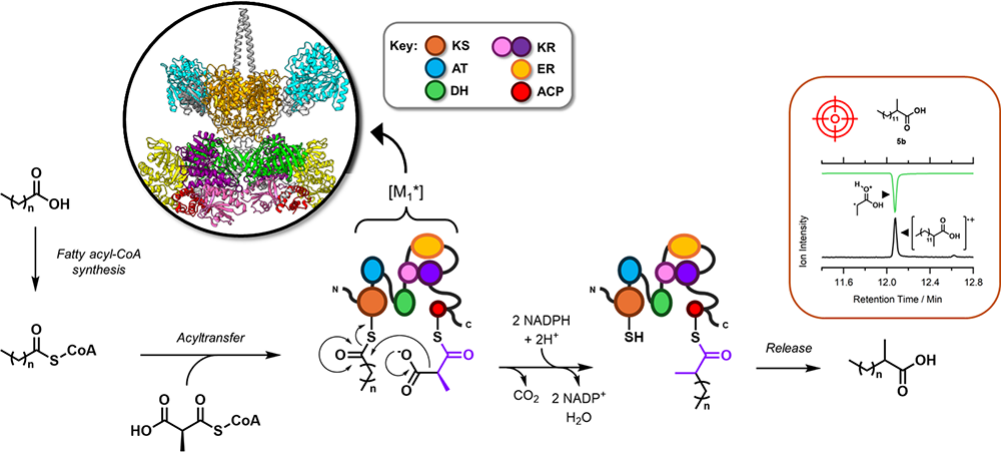

In nature, thousands of diverse and bioactive polyketides are assembled by a family of multifunctional, “assembly line” enzyme complexes called polyketide synthases (PKS). Since the late 20th century, there have been several attempts to decode, rearrange, and “reprogram” the PKS assembly line to generate valuable materials such as biofuels and platform chemicals. Here, the first module from *Mycobacterium tuberculosis* (*Mt*) PKS12, an unorthodox, “modularly iterative” PKS, was modified and repurposed toward the formation of 2-methyl Guerbet lipids, which have wide applications in industry. We established a robust method for the recombinant expression and purification of this modified module (named [M_1_*]), and we demonstrated its ability to catalyze the formation of several 2-methyl Guerbet-like lipids (C_13_–C_21_). Furthermore, we studied and applied the promiscuous thioesterase activity of a neighboring β-ketoacyl synthase (KS) to release [M_1_*]-bound condensation products in a one-pot biosynthetic cascade. Finally, starting from lauric acid, we could generate our primary target compound (2-methyltetradecanoic acid) by coupling the *Escherichia coli* fatty acyl-CoA synthetase FadD to [M_1_*]. This work supports the biosynthetic utility of engineered PKS modules such as [M_1_*] and their ability to derive valuable Guerbet-like lipids from inexpensive fatty acids.

## Introduction

Biocatalysis is the application and manipulation of biological catalysts, or enzymes, toward synthetically useful chemical transformations.^1−3^ Systems that involve crosstalk between many different enzymes can not only sustain the basic functions of life but also generate a myriad of complex natural products (NP), which confer a competitive advantage to their producers. In nature, this chemistry is achieved using renewable and biodegradable feedstocks under relatively benign aqueous conditions. Biocatalysis has benefitted greatly from the significant expansion of engineering toolkits, high-throughput (HTP) screening, metagenomics libraries, and the rapid acceleration of machine learning. Therefore, there is burgeoning potential to replace conventional synthetic routes with artificial one-pot biocatalytic cascades for greener chemical manufacture.

Polyketides are a class of NP synthesized by giant multifunctional biocatalysts called polyketide synthases (PKS).^4,5^ In prokaryotes, such megasynthases are typically multidomain, modular “assembly line” complexes organized from one or more polypeptides. 6-Deoxyerythronolide B synthase (DEBS) from *Saccharopolyspora erythraea* is the archetypical example of such a modular PKS system (Figure S1).^6,7^ A single PKS module is summarily responsible for the elongation of polyketide intermediates by a single ketide unit, in a manner analogous to fatty acid biosynthesis (Figure S2). In brief, the polyketide extension is performed by three catalytic domains within the module: the β-ketoacyl synthase (or simply ketosynthase, abbreviated as KS), the acyltransferase (AT), and the acyl carrier protein (ACP), which requires post-translational modification to its *holo* form by the attachment of a 4′-phosphopantetheine (4′-Ppant) prosthetic group. Together, these domains catalyze the decarboxylative Claisen-like condensation of a malonyl-CoA extender unit with the nascent polyketide intermediate. The β-keto condensation product remains tethered to the ACP and is subsequently processed by a suite of β-processing domains. These include the ketoreductase (KR), the dehydratase (DH), and the enoylreductase (ER). The PKS can encode all, some, or none of these processing domains, thereby furnishing fully, partially, or nonreduced polyketide intermediates. The final polyketide scaffold can be released by a *cis*- or *trans*-acting domain, typically a thioesterase (TE) or a thioester reductase (TR), and further modified (“tailored”) by additional auxiliary enzymes.^8^

As our structural and mechanistic understanding of these complex molecular machines has deepened, so too has the potential to repurpose PKSs toward the production of high-value specialty chemicals.^9,10^ An important chemical target are Guerbet alcohols, acids, and derivatives thereof.^11^ These 2,2-dialkyl fatty molecules have superior lubricity and liquidity compared to their linear stereoisomers and thus have broad applications in industry including lubrication, metalworking, solvation, paper processing, cosmetics, and more. Accordingly, the Guerbet alcohol market was valued at US$1.2 billion in 2022.^12^ Traditionally, Guerbet alcohols are produced via the Guerbet reaction (also known as alcohol coupling), which requires elevated temperatures (180–360 °C), strong alkali, and a metal cocatalyst; this reaction produces β-branched fatty alcohol dimers by sequential dehydrogenation, aldol addition, dehydration, and (re)hydrogenation (Figure S3).^13^ Guerbet alcohols can be further processed to yield useful acid and ester derivatives. Overall, the process requires harsh and energy-intensive reaction conditions, and while a greener chemoenzymatic route toward these chemicals was reported in 2016,^14^ to date, no fully biocatalytic route has been proposed. There is potential, therefore, to build an artificial biosynthetic pathway toward Guerbet-like lipids, where the requisite building blocks can be generated renewably from cheap carbon sources (e.g., glucose, glycerol, or waste carbon-rich resources). To this end, we aimed to contribute some potential biocatalysts that could be used in such a cascade.

**Scheme 1:**
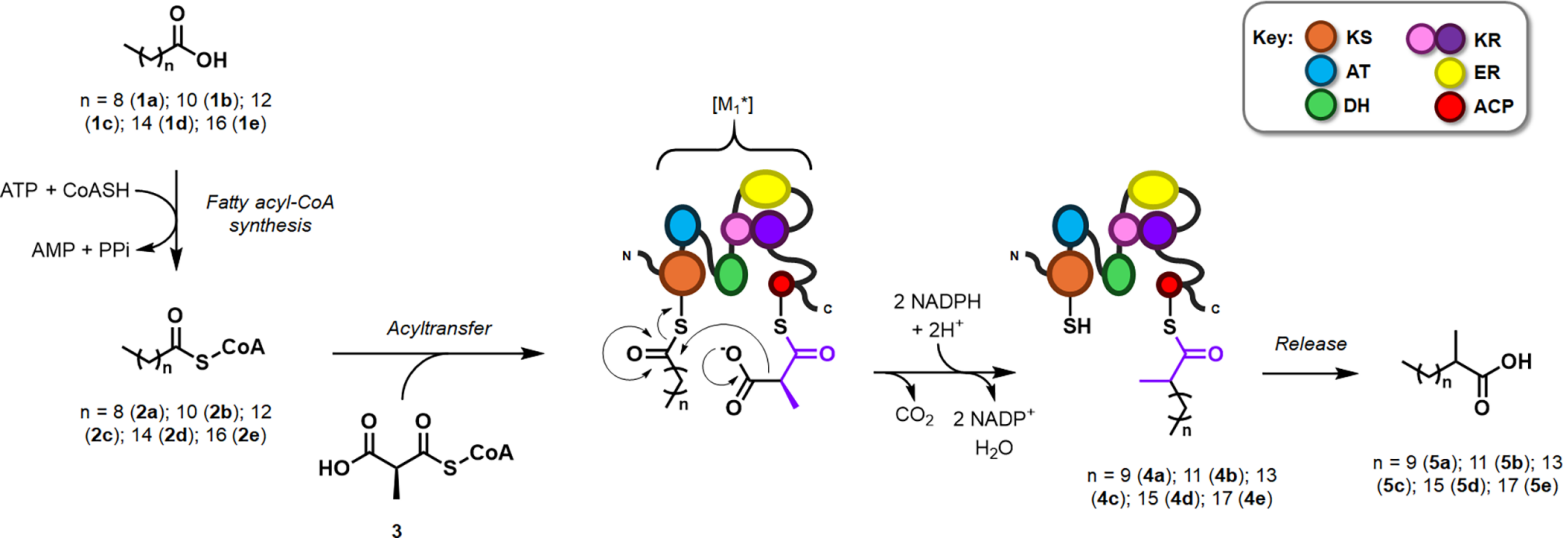
Proposed Utilization of *Mt*PKS12-derived [M_1_*] to Produce 2-Methyl Guerbet-like Lipids The domain color coding shown here is used throughout this work.

We hypothesized that polyketide synthase 12 (PKS12) from *Mycobacterium tuberculosis* (*Mt*) could be repurposed to make Guerbet-like lipids. In nature, *Mt*PKS12 produces the multimethyl-branched antigen mannosyl-β-1-phosphomycoketide (MPM) via a “modularly iterative” catalysis, which involves the head-to-tail supramolecular assembly of several bimodular units via N- and C-terminal docking domains (DDs, see Figure S4).^15−18^ The first module ([M_1_]) in the unit is selective for 2(*S*)-methylmalonyl-CoA (2-MMal-CoA, **3**), whereas the second module ([M_2_]) is selective for malonyl-CoA (Mal-CoA). Both modules encode the necessary domains to furnish fully saturated carbon scaffolds, but only [M_1_] can install methyl branches. By isolating and studying [M_1_] as a standalone biocatalyst, we demonstrate the utility of this PKS module to produce valuable 2-methyl scaffolds **4a**–**e** (Scheme 1). We specially targeted the 2-methytetradecanoyl scaffold **4b** and its valuable fatty acid derivative **5b**, which is used in commercial Guerbet formulations with wide industry applications. In the absence of a suitable chain-terminating enzyme, we investigated and applied the promiscuous TE activity of a KS domain as a novel workaround for the release of ACP-tethered intermediates as fatty acids. Furthermore, we demonstrate the PKS-driven formation of **4b** from lauric acid **1b** by coupling the *Escherichia coli* (*E. coli*) fatty acyl-CoA synthetase FadD (*Ec*FadD), thereby deriving high-value Guerbet-like products from inexpensive fatty acids.

## Results and Discussion

### Design and Recombinant Expression of [M_1_*]

Putative *Mt*PKS12 domain boundaries were predicted and refined using a combination of ClusterCAD,^19,20^ BLASTp,^21^ and AI structural modeling (Figure S5).^22,23^ Catalytic and supporting residues were identified via sequence alignment with homologous, structurally characterized PKS domains. Domains belonging to either [M_1_] or [M_2_] will hereafter be denoted by a numerical subscript (e.g., [ACP_1_] and [KS_2_]).

**Figure 1:**
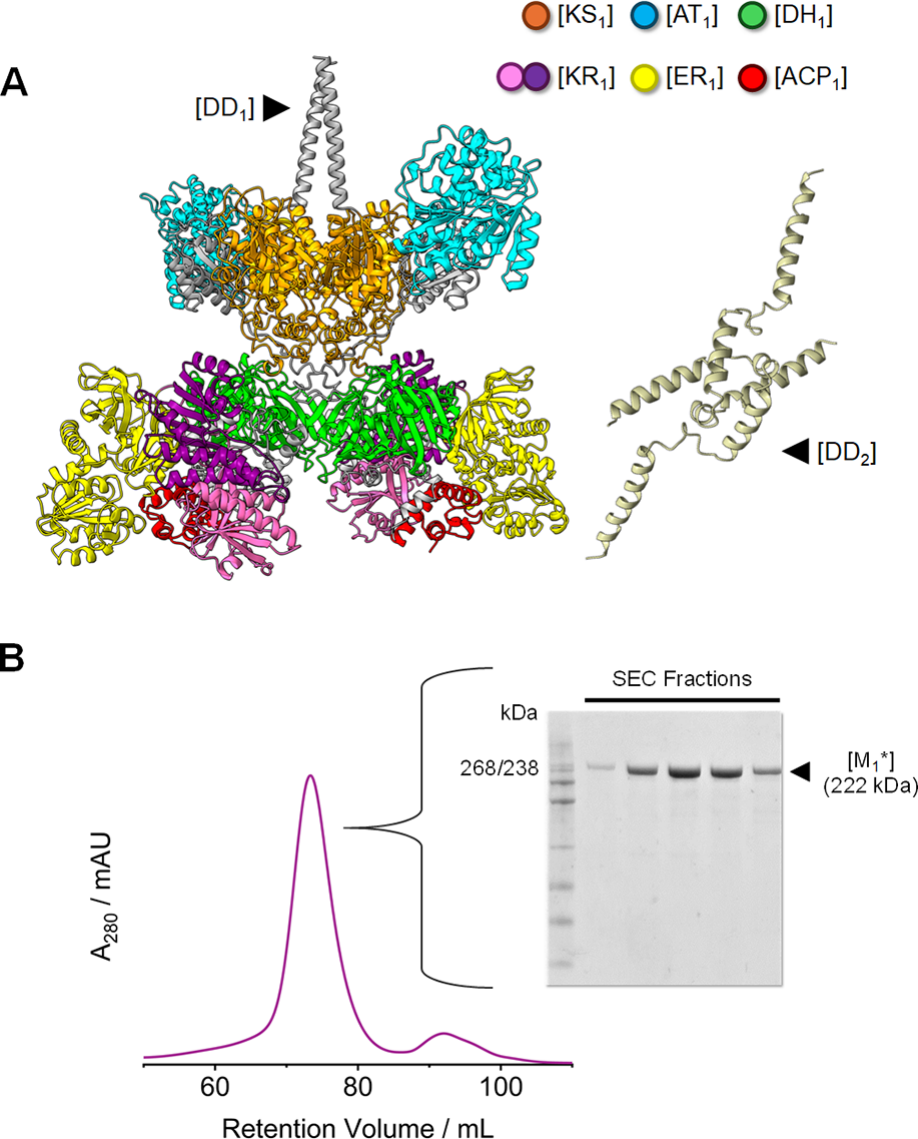
(A) Composite, color-coded structural model of *Mt*PKS12-derived [M_1_*]. All domain structures were predicted using ColabFold. The crystal structure deposited by MuCullough et al. (PDB 8G7W)^24^ was used as a template to approximate the relative organization of the predicted reducing region ([DH_1_][KR_1_][ER_1_]) using structural alignment tools. (B) SEC purification of [M_1_*] monitored by UV–vis spectroscopy (280 nm) and SDS-PAGE. A HiMark prestained protein standard was used as the protein ladder.

The gene encoding *Mt*PKS12 [M_1_] was amplified from *Mt* H37Rv genomic DNA (NCBI accession: NC_000962.3). To improve the structural form of the C-terminus, the C-terminal DD from *Mt*PKS12 [M_2_] ([DD_2_]) was appended to the post-[ACP_1_] linker with an additional poly-His affinity tag for downstream purification. The new sequence therefore differs from the wild type by 97 residues at the C-terminus (Figure S6), with a new theoretical pI and molecular weight (MW) of 5.10 and 221,798.67 Da, respectively. This chimeric module is hereby termed [M_1_*] (Figure 1A and Table S5). [M_1_*] was cloned into pET26b and heterologously expressed in an *E. coli* BW25113 (DE3) Δ*arnA* knockout chassis, cultivated in a 12 L fermenter. Sequential immobilized metal affinity chromatography (IMAC) and size-exclusion chromatography (SEC) enabled the routine preparation of highly pure [M_1_*], with recoveries of approximately 7 mg per gram of wet biomass (Figure 1B). The production and homodimerization of [M_1_*] were confirmed by trypsin digest MS and NativePAGE, respectively (Figure S7 and Tables S6 and S7). In our hands, despite [M_1_*] possessing both the native *Mt*PKS12 N- and C-terminal DDs, we could not find compelling evidence of supramolecular assembly by SEC-multiangle light scattering (Figure S8).

### Probing the Activity of [KR_1_]

**Figure 2:**
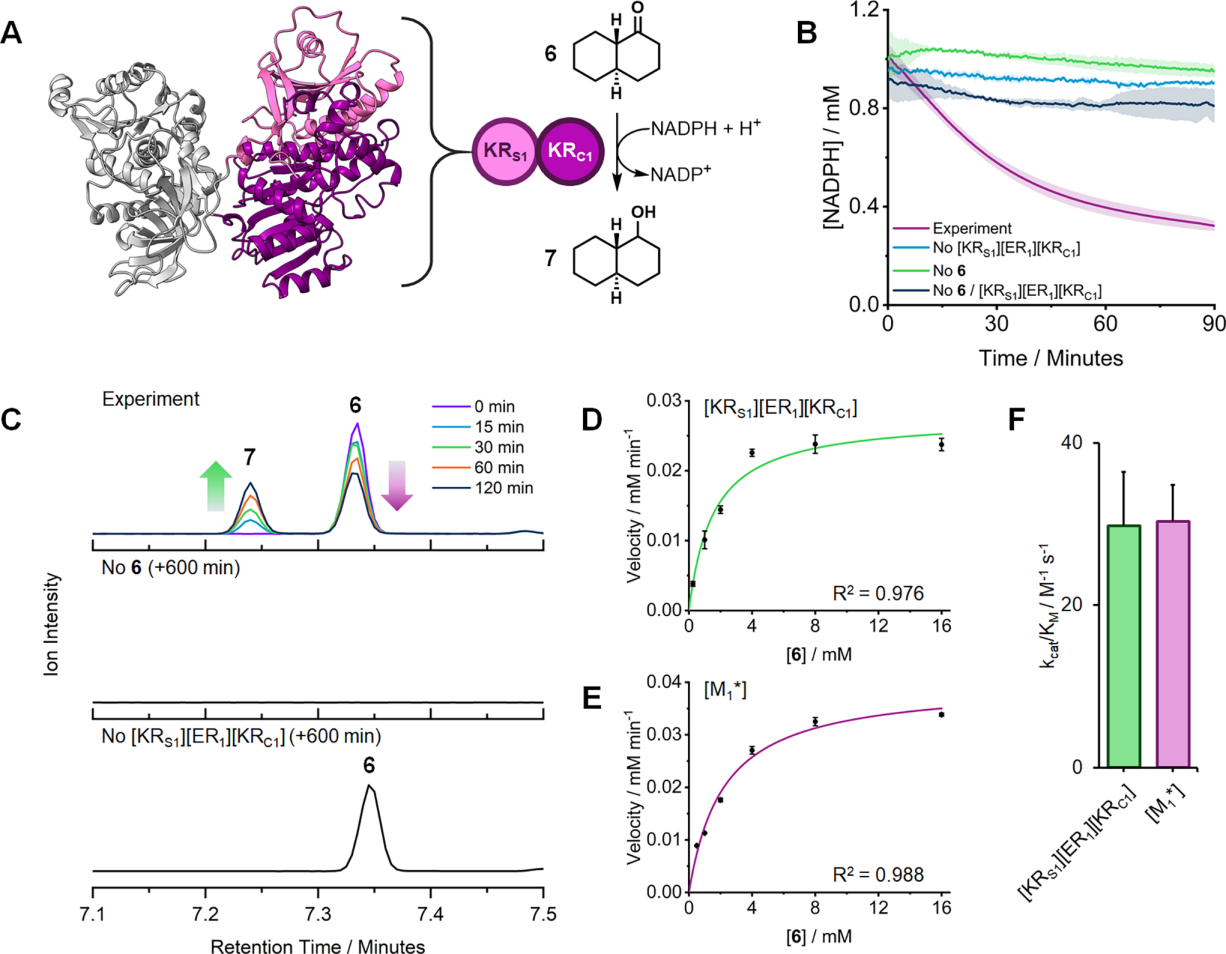
(A) NADPH-dependent reduction of ketone **6** to alcohol **7** by *Mt*PKS12 [KR_1_]. [KR_1_] is composed of structural [KR_S_] and catalytic [KR_C_] subdomains, separated in sequence by [ER_1_]. The AF2 predicted structure of the *Mt*PKS12 [KR_S1_][ER_1_][KR_C1_] didomain is shown ([ER_1_] is grayed out). (B) Monitoring *Mt*PKS12 [KR_1_]-catalyzed reduction of **6** by NADPH depletion (340 nm). All reactions were performed in technical triplicate. Standard deviations are shown as colored error bands. (C) GC/EI-MS chromatograms showing the *Mt*PKS12 [KR_1_]-catalyzed reduction of **6** to **7**. The reaction reaches equilibrium after 120 min (see also Figure S11). Negative controls (sampled after 600 min) are also shown. (D) Michaelis–Menten curve for *Mt*PKS12 [KR_S1_][ER_1_][KR_C1_] using ketone **6**. Error bars represent standard error of three technical replicates. (E) Michaelis–Menten curve for [M_1_*] using ketone **6**. Error bars represent the standard error of three technical replicates. (F) *k*_cat_/*K*_M_ comparison between *Mt*PKS12 [KR_S1_][ER_1_][KR_C1_] and [M_1_*] against ketone **6**. Error bars represent the standard error of three technical replicates.

Following the successful expression and purification of [M_1_*], we next tested the activity of its KR domain. The KR domains of fully/partially reducing PKSs can be targeted using the surrogate alicyclic ketone substrate *trans*-1-decalone **6**.^25,26^ Due to the architectural sophistication of this domain, which requires the association of structural (KR_S_) and catalytic (KR_C_) subdomains, separated in sequence by the ER (Figure 2A),^27−29^ a successful ketoreduction would be indicative of correct protein folding and, crucially, correct domain organization. For our initial tests, we subcloned the [KR_S1_][ER_1_][KR_C1_] didomain (E_1191_-L_1953_) into pET28a with an N-terminal poly-His affinity tag (Figure S9A). Putative hits were identified by restriction digest (Figure S9B) and sequence-confirmed. [KR_S1_][ER_1_][KR_C1_] was overexpressed in *E. coli* BL21 (DE3) and purified using IMAC and SEC. The 83 kDa, 796 aa polypeptide was clearly identified by SDS-PAGE, and an estimated MW of 83,420 Da was returned by LC/ESI-MS using maximum entropy deconvolution, indicating the loss of the N-terminal methionine (83,550–131 Da, see Figure S9C,D). Furthermore, the retention volume of [KR_S1_][ER_1_][KR_C1_] by SEC (73.15 mL) suggests that the didomain is monomeric (∼97 kDa, or ∼1.2-fold the monomeric MW, see Figures S10 and S11 and Table S8); this is consistent with previously reported observations that the ER domains of multimodular PKSs do not form dimeric contacts, unlike those belonging to mammalian FAS and iterative PKSs.^24,29^

Using ketone **6**, the activity of purified [KR_S1_][ER_1_][KR_C1_] was monitored by GC/EI-MS and UV–vis spectroscopy (340 nm). It was shown that, when studied as a standalone biocatalyst, [KR_1_] could catalyze the NADPH-dependent conversion of **6** to *trans*-1-decalinol **7** (Figure 2A,D and Figures S12 and S13), thereby providing the benchmark catalytic activity against which [M_1_*] could be compared (Table S9). Encouragingly, purified [M_1_*] displayed nearly identical reaction kinetics (Figure 2E,F and Table S9), indicating that the KR domain retains its catalytic efficiency when encoded and embedded in the chimeric PKS module. Together with the preceding protein characterization, this result afforded the necessary confidence that [M_1_*] could be produced and folded into an active biocatalyst using *E. coli*.

### *Mt*PKS12 [ACP_1_] *apo*-to-*holo* Conversion

For a PKS module to be condensation-competent, its ACP must be modified with a 4′-Ppant prosthetic group. In this active *holo* form, intermediates are tethered, built, and processed on the ACP via a thioester linkage on the 4′-Ppant group. In nature, the post-translational modification of the carrier protein is performed by a 4′-phosphopantetheinyl transferase (PPTase), which utilizes free coenzyme A (CoASH) and a magnesium (Mg^2+^) cofactor to transfer the 4′-Ppant arm to a highly conserved serine alcohol.^30^ Such *apo*-to-*holo* conversions are conventionally achieved *in vitro* using the *Bacillus subtilis* PPTase known as Sfp (*Bs*Sfp), which displays a surprisingly relaxed selectivity for ACPs.^31^

It was important to test for ourselves the activation of [ACP_1_] using *Bs*Sfp. To this end, His-tagged *Bs*Sfp and *apo*-[ACP_1_] were heterologously expressed in *E. coli* BL21 (DE3) and purified to homogeneity using IMAC and SEC (Figure S14A,B). An *apo*-to-*holo* conversion was performed using a 50:1 molar ratio of *apo*-[ACP_1_] to *Bs*Sfp (Figure S14C). The proteins were coincubated at room temperature with an excess of Mg^2+^, CoASH, and dithiothreitol (DTT). After 1 h, a sample of the reaction mixture was diluted 10-fold in dH_2_O prior to accurate mass analysis by LC/ESI-MS. The clear and quantitative modification of *apo*-[ACP_1_] via Ser_1998_ was signified by an anticipated 340 Da MW increase (13,624.53 ± 0.06 Da) compared to the *apo*-[ACP_1_] control without *Bs*Sfp (13,284.72 ± 0.02 Da), with no detectable trace of the *apo*-form present (Figures S14D and S15–S17 and Table S10). Thus, we confirmed that *Bs*Sfp can be utilized to generate *holo*-[ACP_1_] *in vitro*, and we subsequently deployed it to generate *holo*-[M_1_*].

### [M_1_*] Can Produce C_13_–C_21_ Lipid Scaffolds

**Figure 3:**
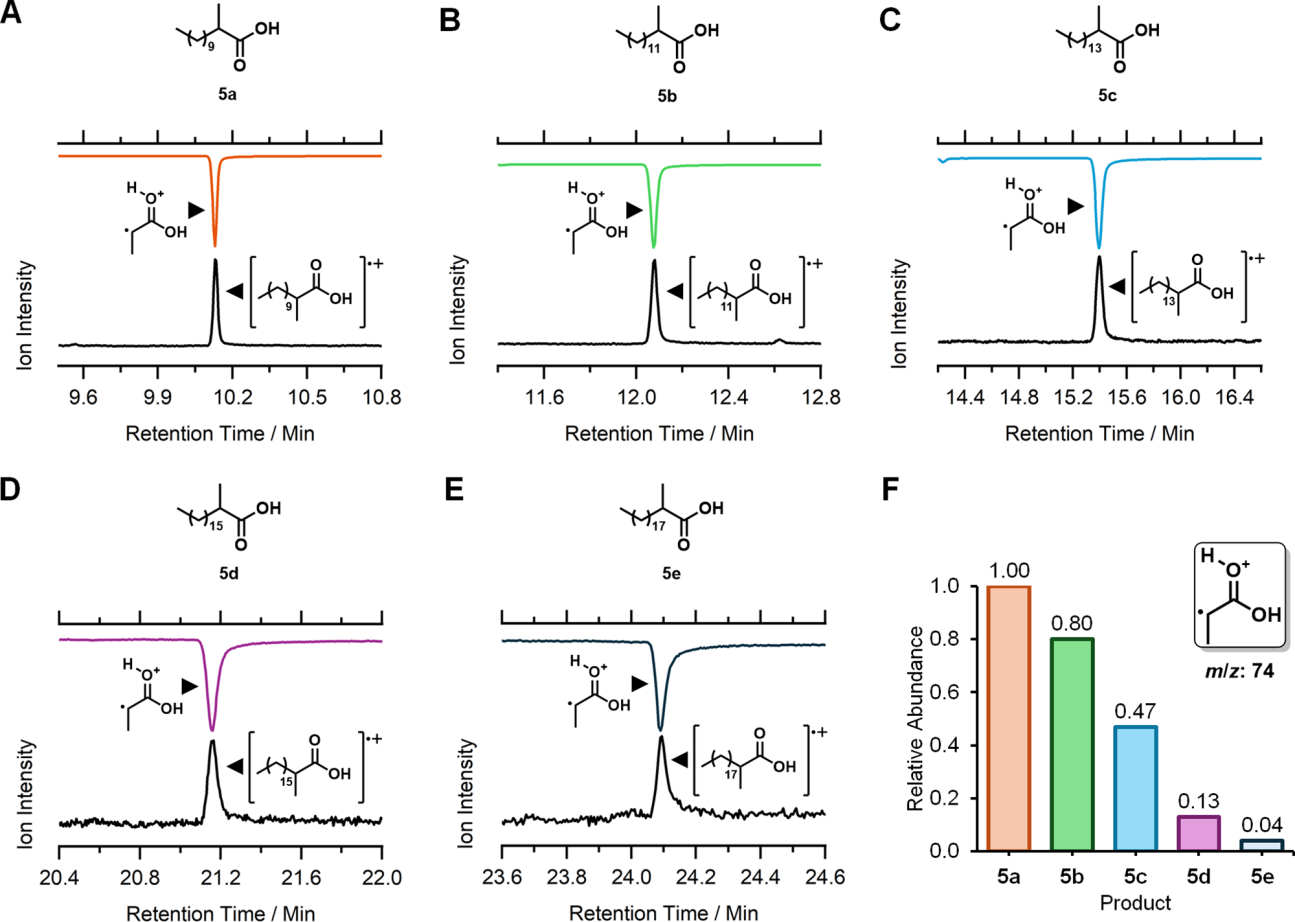
GC/EI-MS analysis of [M_1_*]-derived **5a**–**e**, detected using SIM. The chromatograms show the simultaneous detection of the molecular ion (bottom chromatograms, black) and the common McLafferty product ion (top inverted chromatograms, colored) for each analyte. (A) SIM detection of **5a**. (B) SIM detection of **5b**. (C) SIM detection of **5c**. (D) SIM detection of **5d**. (E) SIM detection of **5e**. (F) Relative abundance of [M_1_*]-derived products, calculated from the intensity of the common McLafferty product ion.

It was reasoned that, similarly to its parental *Mt*PKS12, *holo*-[M_1_*] could utilize a range of acyl-CoA substrates for condensation with 2-MMal-CoA **3**. In the absence of an auxiliary enzyme to release condensation products from [M_1_*], a single turnover assay featuring 8.9 mg of pure *holo*-[M_1_*], 500 μM **3**, and one of five fatty acyl-CoAs (**2a**–**e**, C_10_–C_18_) was initiated by the addition of NADPH. Following overnight incubation, the corresponding 2-methyl condensation products **4a**–**e** were released by alkaline hydrolysis at 50 °C, protonated, and extracted into ultrapure EtOAc. The organic extract was concentrated *in vacuo* and analyzed by GC/EI-MS in selective ion monitoring (SIM) mode (Figure S18). Since the chemical workup was expected to yield 2-methyl Guerbet-like acids **5a**–**e** (C_13_–C_21_), the mass analyzer was configured to scan for two diagnostic ions: the molecular ion (*m*/*z* = 214, 242, 270, 298, or 326) and the common product ion derived from the McLafferty rearrangement (*m*/*z* = 74). Using this strategy, all five Guerbet-like acids were identified on their respective gas chromatograms by the simultaneous detection of both diagnostic ions (Figure 3A–E and Figure S19). It was particularly encouraging to observe that the desired **4b** scaffold could be produced by [M_1_*], with no detectable trace of condensation products in the controls (Figure S20). The recovery of **5b** using our workup procedure was further verified using a commercial standard (Figures S21 and S22 and Table S11). A full EI-MS spectrum was subsequently collected and assigned for each product (Figure S23). The relative abundance of **5a**–**e**, calculated from the intensity of the common McLafferty product ion, suggests that [M_1_*] can process shorter substrates more efficiently under the reaction conditions studied (Figure 3F). Following the successful demonstration of condensation activity, we next sought a means to release [M_1_*]-tethered products enzymatically.

### Leveraging the Promiscuous TE Activity of KS Domains for Product Release

Since neither *Mt*PKS12 nor [M_1_*] encodes a chain-terminating domain, they cannot release condensation products from their ACPs without a *trans*-acting enzyme. In the *Mt* H37Rv genome, immediately downstream of the coding sequence (CDS) for *Mt*PKS12, is a gene that encodes a bifunctional short-chain dehydrogenase/reductase-phosphotransferase (SDR-PT) fusion enzyme (UniProt P9WIH5).^32^ It is hypothesized that this unique enzyme reductively cleaves and phosphorylates polyketides from *Mt*PKS12, thereby supplying the monoalkylphosphate substrates for polyprenol monophosphomannose synthase 1 (Ppm1, encoded by *rv2051c*, see Figure S24).^16,33^ Unfortunately, in our hands, all efforts to express this protein in *E. coli* were unsuccessful due to total insolubility (Figure S25). Furthermore, no success was achieved when we attempted to incubate [M_1_*] with a selection of offloading enzymes from known ACP-dependent/thiotemplated pathways, such as those involved in (phenol)phthiocerol dimycocerosic acid,^34,35^ tambjamine YP1,^36^ and coelimycin P1^37^ biosynthesis (data not shown). Short of an HTP panel of TEs/TRs, we therefore required an alternative strategy to release [M_1_*]-tethered intermediates.

In multimodular PKSs, intermediates are shuttled from one module to the next via interactions between the acyl-ACP and its downstream cognate KS (Figure S26). This transfer involves moving the nascent intermediate to a specific catalytic cysteine residue located in the active site of the KS. We reasoned that, in isolation, any acyl-KS Michaelis complex will eventually hydrolyze to yield a fatty acid and regenerate the catalytic cysteine thiol. This hypothesis was partly inspired by bacillaene KS crystal structures (PDB 4NA2 and 4NA3) from *Bacillus subtilis*,^38^ which feature substrates trapped in the active site thanks to a Cys→Ser mutation. In these examples, His_311_ (required for condensation, hereby termed His^C^) and His_330_ (required for acyltransfer, hereby termed His^T^)^39^ position the water molecule within a feasible attack distance from the ester carbonyl (Figure S27). Following this observation, we hypothesized that His^C^ and His^T^ may possess mild water-activating properties to permit thioester hydrolysis, analogous to TEs, which utilize a Ser/Cys-Asp-His catalytic triad. This promiscuous TE activity would allow the KS to protect itself against irreversible inhibition; it may also prevent unwanted/incorrect polyketide intermediates from advancing for further elongation.

**Figure 4:**
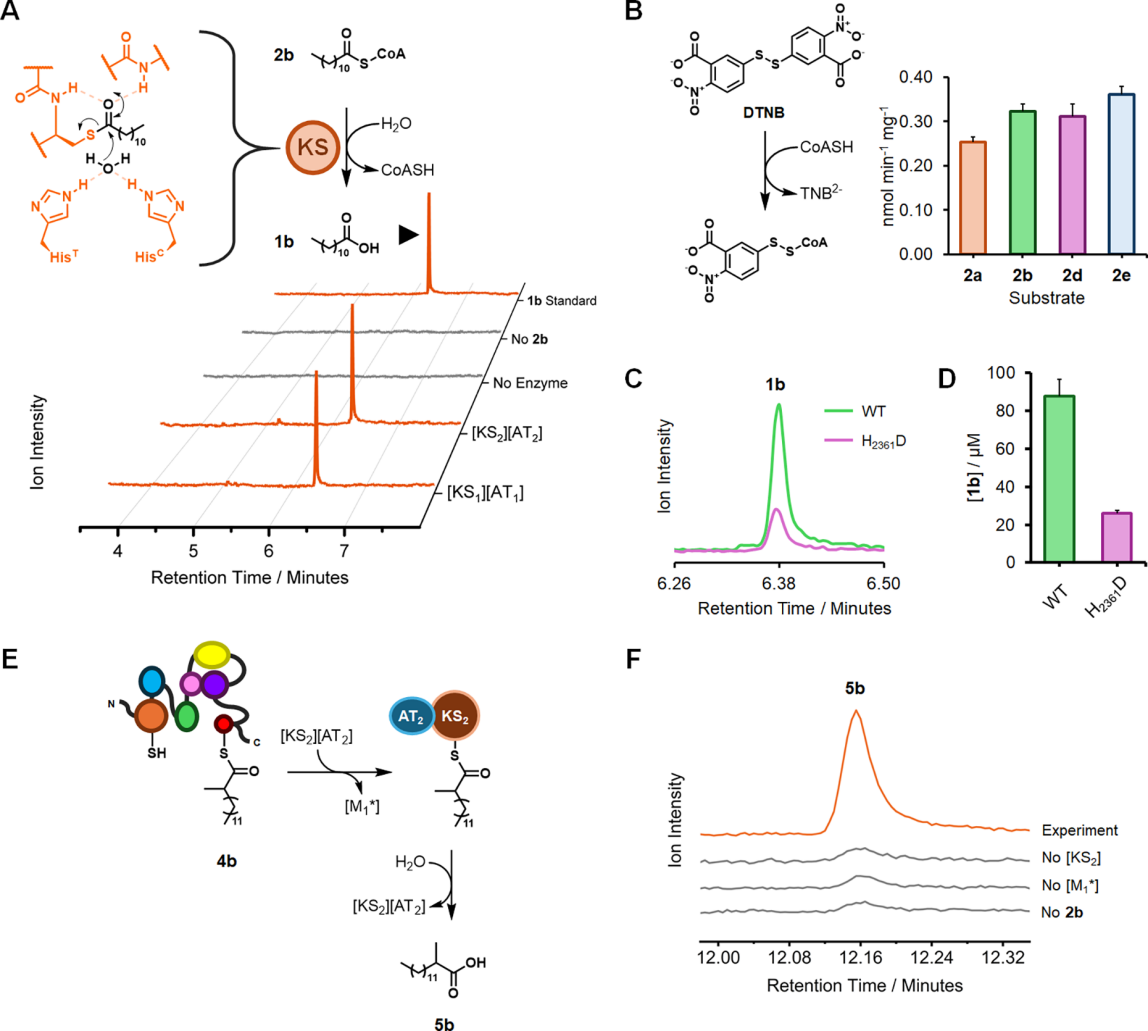
(A) GC/EI-MS chromatograms demonstrating the promiscuous TE activity of *Mt*PKS12 KS domains, alongside negative controls. Chromatograms were recorded after 19.5 h of incubation with **2b**. The proposed hydrolysis of the acyl-[KS_1_] Michaelis complex is also shown. (B) [KS_1_] TE activity against a range of acyl-CoAs (**2a**, **2b**, **2d**, and **2e**), detected using the DTNB assay. Spectrophotometric detection of the TNB^2–^ anion (412 nm) was recorded after 18 h. Background absorbance from nonenzymatic acyl-CoA hydrolysis, as well as the reaction of DTNB with surface-exposed [KS_1_][AT_1_] cysteines, was subtracted from all experimental values. Error bars represent the standard deviation of three technical replicates. (C) Representative GC/EI-MS chromatograms showing the TE activity of wild-type (WT) [KS_2_] and mutant H_2361_D [KS_2_] against **2b**. (D) Quantified **1b** titers using WT and H_2361_D [KS_2_]. Error bars represent the standard deviation of three technical replicates. (E) Proposed [KS_2_]-mediated acyl transfer and hydrolysis of **4b** giving **5b**. (F) GC/EI-MS chromatograms showing the successful [KS_2_]-mediated release of **5b**. The instrument was configured for SIM (*m*/*z* = 74).

In support of this hypothesis, it was shown by GC/EI-MS that 10 μM recombinant *Mt*PKS12 [KS_1_] (expressed as a [KS_1_][AT_1_] didomain, see Figure S28) could slowly hydrolyze an excess of lauroyl-CoA **2b** (Figure 4A and Figure S29). This effect was also demonstrated by capturing the CoASH byproduct using 5,5'-dithio-bis(2-nitrobenzoic acid) (DTNB); using this colorimetric assay, we report TE behavior against a range of acyl-CoAs (Figure 4B and Table S12). A similar hydrolytic activity was identified using the KS domain from *Mt*PKS12 [M_2_] (hereby termed [KS_2_], expressed as a [KS_2_][AT_2_] didomain, see Figure 4A and Figure S30C). Interestingly, when His^C^ (His_2361_) in [KS_2_] was mutated to Asp (Figure S30) to mimic a typical TE catalytic triad, the observed hydrolytic activity instead dropped 4-fold relative to the wild type (Figure 4C,D); the presence of His^C^ is important, but not critical, for TE activity. Following this observation, we hypothesized that wild-type [KS_2_] could be used to transfer and release products from [M_1_*] as Guerbet-like acids, in effect repurposing a thiolase as a pseudo acyl-ACP hydrolase (Figure 4E). To test this, 20 μM [KS_2_][AT_2_] was incubated with 10 μM *holo*-[M_1_*], 500 μM **2b**, 500 μM 2-MMal-CoA **3**, and 2 mM NADPH. The reactions were quenched after 18 h using ultrapure EtOAc, and the organic phase was recovered and sampled for GC/EI-MS analysis. Gratifyingly, the expected **5b** product was identified only when [KS_2_] was coupled with [M_1_*] (Figure 4F), indicating that the release of **5b** is enabled by the TE activity of [KS_2_] and not by background hydrolysis of intermediate **4b**. These data suggest that the promiscuous chain-terminating behavior of [KS_2_] can be leveraged to release Guerbet-like lipids (and possibly polyketide intermediates more broadly) as fatty acids. To the best of our knowledge, this marks the first time that a Guerbet product has been produced in a one-pot, artificial biosynthetic cascade.

### [M_1_*] Production of 2-Methyltetradecanoic Acid **5b** from Lauric Acid Using a Fatty Acyl-CoA Synthetase

**Figure 5:**
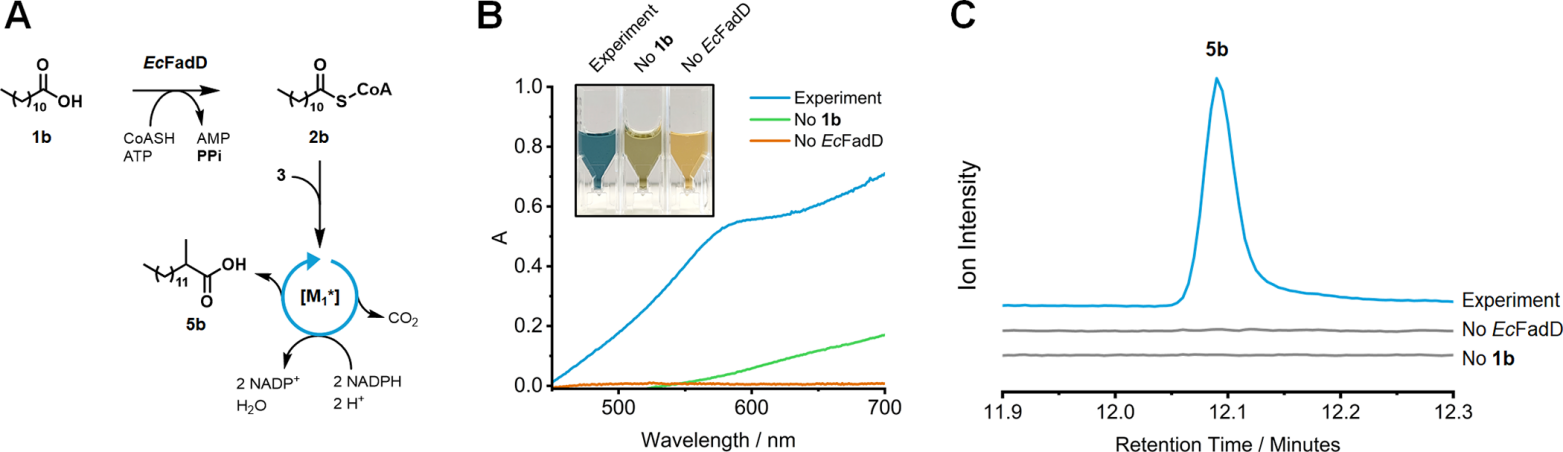
(A) Proposed coupling of *Ec*FadD to [M_1_*] to produce **5b** from **1b** via **2b**. (B) Spectrophotometric detection of the *Ec*FadD-catalyzed conversion of **1b** to **2b** using the ammonium molybdate assay. The released PPi forms a blue phosphomolybdate complex in the presence of a reducing agent (λ_max_ = 580 nm). (C) GC/EI-MS chromatograms showing the successful *Ec*FadD-[M_1_*] coupled production of **5b** from **1b**. The instrument was configured for SIM (*m*/*z* = 74).

Our *in vitro* methods for synthesizing **5a**–**e** have so far relied on costly fatty acyl-CoAs as substrates for [M_1_*]. To make the conversion more cost-effective for future scaled-up manufacturing, it was important to integrate an additional enzyme that can supply the requisite fatty acyl-CoAs *in situ* while also serving as an iterative acyl-CoA recycling system. Accordingly, we identified *Ec*FadD (UniProt P69451)^40^ as an ideal candidate for this role (Figure 5A). In our initial qualitative test, *Ec*FadD was recombinantly expressed, purified (Figure S31), and applied to generate lauroyl-CoA **2b***in vitro* using inexpensive lauric acid **1b**, ATP, and Mg^2+^. The successful production of **2b** from **1b** was indicated by the accumulation of inorganic pyrophosphate (PPi) in the reaction, which was detected using the well-established ammonium molybdate assay (Figure 5B).^41^ Following this successful activity demonstration, we next attempted to couple *Ec*FadD to [M_1_*] to produce **5b** using **1b** and 2-MMal-CoA **3**. This strategy led to the successful generation of target **5b** from an excess of **1b** (Figure 5C). The integration of *Ec*FadD into our PKS-driven pathway represents a practical innovation that could enable the biobased production of Guerbet-like lipids.

## Concluding Remarks

In this study, our objective was to identify potential biocatalysts for sustainable Guerbet-like lipid production, with a particular focus on accessing 2-methyltetradecanoyl carbon scaffolds. To this end, we created a chimeric PKS module [M_1_*], derived from *Mt*PKS12, and characterized its constitutive catalytic domains in a bottom-up foundational study. We then demonstrated the ability of [M_1_*] to generate a range of 2-methyl fatty scaffolds (**4a**–**e**), with an emphasis on the 2-methyltetradecanoyl scaffold **4b** and its corresponding acid **5b**. In the absence of a compatible chain-terminating enzyme, we instead studied and applied the promiscuous TE activity of *Mt*PKS12 [KS_2_] to generate our target **5b** in a one-pot biosynthetic cascade. Finally, we coupled an efficient fatty acyl-CoA synthetase *Ec*FadD to [M_1_*], which enabled access to the target C_15_ product from inexpensive lauric acid.

We envision that the range of Guerbet-like chemicals can be diversified by engineering the AT domain to accept more unorthodox extender units (e.g., ethyl/propyl/butylmalonyl-CoA); the AT motif swap recently demonstrated by Kalkreuter et al. is a promising development in this field.^42^ Furthermore, we expect that [M_1_*] catalysis can be enhanced by the identification of an efficient and selective chain-terminating enzyme; to this end, it would be greatly beneficial to create and screen a broad panel of TEs/TRs against acyl-[ACP_1_]. Through strategic enzyme engineering and cascade optimization, we anticipate that this work can be developed from its current proof-of-concept stage toward a scalable bioprocess for Guerbet lipid production.

## Supporting Information Available

Details of materials and experimental methods, supporting data, and predictive structural modeling and plasmid maps (PDF)

## ABBREVIATIONS

NADPH: reduced nicotinamide adenine dinucleotide phosphate
NADP^+^: nicotinamide adenine dinucleotide phosphate
CoA: coenzyme A
GC/EI-MS: gas chromatography/electron ionization mass spectrometry
LC/ESI-MS: liquid chromatography/electrospray ionization mass spectrometry
MSD: mass-selective detector
UV–vis: ultraviolet–visible
ATP: adenosine triphosphate
AMP: adenosine monophosphate
