# Supplementary material for: Repurposing a Fully Reducing Polyketide Synthase toward 2-Methyl Guerbet-like Lipids
Source: ACS Catal. 2024 Oct 31;14(22):16834–42. doi: 10.1021/acscatal.4c04714 (PMC11574752; doi:10.1021/acscatal.4c04714)
Supplement: Supplementary file 1 — cs4c04714_si_001.pdf [file cs4c04714_si_001.pdf]

Supporting Information (SI)

for

Repurposing a Fully-Reducing Polyketide Synthase Towards 2-Methyl Guerbet-like Lipids

Michael A. Herrera, Stephen McColm, Louise-Marie Craigie, Joanna Simpson, Fraser Brown, David J. Clarke, Reuben Carr and Dominic J. Campopiano

Table of Contents

Materials 4

Buffers and Reagents. 4

Plasmids, Strains and Proteins. 4

GC/EI-MS Sample Preparation, Hardware and Instrument Configuration 4

Methods 5

Protein Sequence Analysis. 5

Protein Structure Prediction. 5

Creation of BW25113 (DE3) Δ*arnA* Knockout. 5

General Procedure for Protein Expression and Purification. 5

Creation of pET28a-*Mt*PKS12 [KRS_1][ER_1][KRC_1] (pIES02). 6

Monitoring Ketoreduction by Spectrophotometry. 6

Monitoring Ketoreduction by GC/EI-MS. 6

Preparation and Analysis of [M_1*]-derived 2-Methyl Guerbet Acids 5a-e 6

Monitoring *Mt*PKS12 KS Thioesterase Activity by GC/EI-MS. 7

Monitoring *Mt*PKS12 [KS_1] Thioesterase Activity by Spectrophotometry. 7

Creation of *Mt*PKS12 [KS_2][AT_2] H_{2361}D Mutant (pIES12). 7

[M_1*] + *Mt*PKS12 [KS_2][AT_2] Assay. 7

Spectrophotometric detection of *Ec*FadD activity using ammonium molybdate. 8

*Ec*FadD + [M_1*] Coupled Assay. 8

Supplementary Tables 9

Table S1. Summary of buffers, rich media and reagent stocks. 9

Table S2. Plasmid table and purification summary. 9

Table S3. Primers used for subcloning *Mt*PKS12 [KR_{S1}][ER_1][KR_{C1}] (pIES02). 10
Table S4. Primers used for *Mt*PKS12 [KS_2][AT_2] mutagenesis. 10
Table S5. Domain annotation for [M_1*]. 10
Table S6. Trypsin digest MS results for [M_1*] 11
Table S8. HiLoad 16/600 Superdex 200 (120 mL) SEC calibration used to estimate native protein MW. 13
Table S9. Michaelis-Menten kinetics for the ketoreduction of 6 using *Mt*PKS12 [KR_{S1}][ER_1][KR_{C1}] and [M_1*] 13
Table S10. LC/ESI-MS analysis of *Bs*Sfp and *Mt*PKS12 [ACP_1]. 13
Table S11. Calibration data for 5b (2-methyltetradecanoic acid). 14
Table S12. [KS_1][AT_1] TE specific activity calculations. 14
Supplementary Figures 15
Fig. S1. 2D representation of DEBS, the archetypal multi-modular PKS. 15
Fig. S2. Catalytic cycle of a fully-reducing PKS module embedded in a multi-modular PKS. 16
Fig. S3. The preparation of 2,2-dialkylethanols (Guerbet alcohols) via the Guerbet reaction. 16
Fig. S4. The "modularly-iterative" catalysis of *Mt*PKS12. 17
Fig. S5. Confidence metrics for the predicted domain fragments of *Mt*PKS12 [M_1]. 18
Fig. S6. The C-terminus of [M_1*] aligned with the wildtype *Mt*PKS12 [M_1] and [M_2]. 19
Fig. S7. NativePAGE analysis. 20
Fig. S8. SEC-MALS analysis of [M_1*] 20
Fig. S9. Cloning and Purification of *Mt*PKS12 [KR_{S1}][ER_1][KR_{C1}]. 21
Fig. S10. Calibration plot for the HiLoad 16/600 Superdex 200 pg (120 mL) SEC column 22
Fig. S11. SEC purification of [KR_{S1}][ER_1][KR_{C1}] 22
Fig. S12. Supplementary GC/EI-MS data for *trans*-1-decalone assay. 23
Fig. S13. Raw GC/EI-MS data of *cis/trans*-1-decalone 24
Fig. S14. *Mt*PKS12 [ACP_1] handling and post-translational modification by *Bs*Sfp. 25
Fig. S15. LC/ESI-MS charge envelope of *Bs*Sfp 26
Fig. S16. LC/ESI-MS charge envelope of *Mt*PKS12 *apo*-[ACP1] 26
Fig. S17. LC/ESI-MS charge envelope of *Mt*PKS12 *holo*-[ACP1] 27
Fig. S18. A graphical summary of the preparation and analysis of [M_1*] condensation products 28
Fig. S19. Overlaid GC-EI/MS chromatograms showing the detection of [M_1*] Guerbet-like acids 5a-e and acyl-CoA derived fatty acids 1a-e. 29
Fig. S20. Detection of [M_1*]-derived 5b (2-methyltetradecanoic acid) by GC/EI-MS. 30
Fig. S21. Commercial standard of 5b (2-methyltetradecanoic acid) by GC/EI-MS. 31
Fig. S22. 5b (2-methyltetradecanoic acid) calibration plot. 31
Fig. S23. Experimentally-determined EI-MS of [M_1*]-derived products 32

Fig. S24. Putative MPM BGC in *M. tuberculosis* H37Rv. 33
Fig. S25. SDS-PAGE analysis showing the total insolubility of expressed Rv2047c 34
Fig. S26. Modelling the mutual recognition between *Mt*PKS12 [ACP_1] and PKS12 [KS_2]. 34
Fig. S27. Superimposition of modified Bacillaene [KS_2] catalytic triads (PDB 4NA2 and 4NA3) . 35
Fig. S28. Purification of *Mt*PKS12 [KS_1][AT_1]. 35
Fig. S29. *Mt*PKS12 [KS_1][AT_1]-catalysed hydrolysis of lauroyl-CoA (2b) to give lauric acid (1a). 36
Fig. S30. *Mt*PKS12 [KS_2][AT_2] mutagenesis and purification. 37
Fig. S31. Expression and purification of *Ec*FadD using HisTrap HP resin. 38
Plasmid Maps 39
Sfp-pACYC 39
pISL52 40
pIES01 40
pIES02 42
pIES03 43
pIES11 44
pIES12 45
References 46

Materials

Buffers and Reagents. Acyl-CoAs, CoASH, fatty acids (C_{10}-C_{18}), *trans*-1-decalone, *cis*-1-decalinol, chloramphenicol and kanamycin sulfate were purchased from Sigma-Aldrich. 2-methyltetradecanoic acid was purchased from BLDpharm. Ultrapure EtOAc (Hypergrade, LiChrosolv for LC-MS) was purchased from Supelco. NADPH was purchased from Acros Organics (now Thermo Scientific). Imidazole, DTT and IPTG were purchased from Fluorochem. Yeast extract and tryptone were purchased from Merck Life Sciences, and SOC recovery medium (for transformations) was purchased from New England Biolabs. All other chemicals and solvents were purchased from Fisher Scientific. Deionised water (dH_2O) for buffers, media and reagent stocks was obtained using a Sartorius purifier. All media was sterilised by autoclave prior to use. All buffers (A-E, see table S1) for protein purification were filtered (0.22 μm) and de-gassed under vacuum.

Plasmids, Strains and Proteins. A summary of all plasmids, primers and proteins used in this study can be found in table S2. Plasmids Sfp-pACYC, pISL52, pISL53 were provided by Ingenza Ltd. Plasmid pET28a-*fadD* was kindly provided by the Vito Valiante research group. Plasmids pIES01, 03, and 11 were synthesised and cloned by Genscript. Plasmids pIES02 and pIES11 were created in-house. Primers were designed with the assistance of SnapGene Viewer and webtools provided by New England Biolabs (Tm Calculator) and Merck (Oligos in Tubes). Desalted, dry primers were ordered through Merck. Materials for DNA amplification, Gibson Assembly, mutagenesis and restriction digest were purchased from New England Biolabs. Plasmids and PCR products were purified using GeneJet kits purchased from Thermo Scientific. Plasmids were propagated using sub-cloning efficiency *E. coli* DH5a (Invitrogen) or NEB 5-alpha (New England Biolabs) cells. DNA sequencing was performed by Eurofins Genomics via their GATC SupremeRun Sanger sequencing service. For protein production, *E. coli* BL21 (DE3) was purchased from Agilent and *E. coli* BW25113 (DE3) was provided by Ingenza Ltd. All purification hardware (including ÄKTA Start and ÄKTA Go FPLC purifiers) were purchased from Cytiva. SDS-PAGE was performed using pre-cast NuPAGE 4-12% Bis-Tris gels and NuPAGE MES running buffer purchased from Invitrogen and used according to manufacturer protocol. NativePAGE 4-16% Bis-Tris gels, sample buffers and running buffers were purchased from Invitrogen and used according to manufacturer protocol. Protein gels were stained using InstantBlue Coomassie stain (Abcam). Where possible, purified proteins were characterised using a Waters SYNAPT G2 HDMS. For [M_1*], protein bands were excised from SDS-PAGE gels and submitted to the BSRC Mass Spectrometry Facility (University of St Andrews) for trypsin digest MS. SEC-MALS characterisation of [M_1*] was performed by the Edinburgh Protein Production Facility (EPPF).

GC/EI-MS Sample Preparation, Hardware and Instrument Configuration. Ultra-pure LC-MS-grade EtOAc was used to recover hydrophobic volatiles and semi-volatiles from aqueous biocatalytic reactions. The organic phase was recovered using a Heraeus Pico 17 microcentrifuge (Thermo Scientific) and sampled into screw thread autosampler vials with a 250 μL conical insert. All GC/EI-MS analyses were performed using a Shimadzu QP2010 SE fitted with a Zebron ZB-1ms capillary column (Phenomenex, serial number: 1096275, dimensions: 25 mm internal diameter, 25 μm film thickness, 30 m length). Prior to first use, the column was baked at 350 °C for several hours to suppress column bleed. 1 μL sample was injected (split 10:1 or 50:1, 230 °C inlet temperature using a Restek Topaz 3.5 mm ID quartz wool inlet liner) and chromatographically resolved under 1 mL min^{-1} constant helium flow. For total ion chromatograms, the MS was configured to detect ions over a range of 40-620 m/z. The ion source and transfer line temperatures were set to 200 °C. For each experiment involving GC/EI-MS, the oven parameters are specified in the experimental.

Methods

Protein Sequence Analysis. *Mt*PKS12 domain boundaries were determined using a combination of ClusterCAD[1] and BLASTp. BLASTp was configured to search non-redundant sequences (NRS), UniProt KB/Swiss-Prot or Protein Data Bank (PDB) databases for homologous sequences. Multiple sequence alignments (MSA) and percent identity matrices were computed using Clustal Omega[2] using default parameters. MSAs were visualised using ESPript 3.[3] Genome analysis was performed using antiSMASH[4] (bacterial version) using default parameters. Theoretical molecular weights (MWs) and isoelectric points (pI) were computed using ProtScale.[5]

Protein Structure Prediction. All structural predictions were performed using ColabFold.[6,7] In brief, a deep MSA was generated using MMSeqs2 prior to structure prediction using AlphaFold 2 (structural templates were not utilised for prediction). When appropriate, ColabFold was configured to perform homodimeric prediction. The output of the AlphaFold 2 structure module was recycled up to 6 times for refinement. For each sequence, a total of 5 models were generated and ranked by Predicted Template Model score (pTM); Predicted Local Distance Difference Test (pLDDT) scores were also computed for each model to evaluate fold-level confidence. The best model was subsequently relaxed to eliminate steric clashes. Visual inspection was performed in UCSF ChimeraX (v1.3).[8] Composite structural models were assembled in PyMOL (v3).

Creation of BW25113 (DE3) Δ*arnA* Knockout. BW25113 (DE3) Δ*arnA* knockouts were prepared by Lambda Red recombineering *as per* literature protocol.[9,10] In brief, the DNA substrate (encoding a kanamycin selective marker flanked by flippase recognition target (FRT) sites and *arnA* homology regions) was introduced into electrocompetent BW25113 (DE3) (expressing the Red recombinase proteins Exo, Bet and Gam Red, encoded by pKD46) by electroporation. Colonies were developed on kanamycin (50 μg mL^{-1}) LB-agar plates and successful knockouts were identified by colony PCR. Knockout colonies were subsequently cured by Flp-FRT recombination,[11,12] propagated in LB media (5 mL, 37 °C overnight with agitation) and stored in glycerol (20% v/v) at -80 °C until needed. From these cell banks, BW25113 (DE3) Δ*arnA* knockouts were made chemically-competent by subculturing in LB media (5 mL, 37 °C), harvesting at early log phase and washing the biomass using sterile, ice-cold $CaCl_2$ (100 mM). The washed cells were resuspended in ice-cold $CaCl_2$ (100 mM, 2 mL), sub-aliquoted as required and transformed fresh.

General Procedure for Protein Expression and Purification. Using the desired expression construct, chemically-competent *E. coli* BL21 (DE3) cells (Agilent) or BW25113 (DE3) Δ*arnA* cells were transformed by the heat-shock method. Transformant colonies were developed at 37 °C overnight on LB-agar plates supplemented with the appropriate antibiotic (see table S2). A single transformant colony was propagated in LB-media (150-300 mL) with antibiotic. The liquid culture was incubated overnight at 37 °C with agitation. Afterwards, the cells were subcultured (OD_{600} = 0.1) at 37 °C in fresh LB media (600 mL) supplemented with antibiotic until an OD_{600} of 0.6-0.8 was reached. The subcultures were cooled to room temperature and protein expression was induced by the addition of IPTG (0.1 mM). Protein expression proceeded overnight at 16 °C with sufficient agitation for good oxygen mass transfer. The biomass was harvested by centrifugation using a Fiberlite F14-6 x 250y fixed-angle rotor (7000 rpm, 5 minutes), combined into 2-5 g pellets using a Fiberlite F15-8 x 50cy fixed angle rotor (5000 rpm, 10 minutes) and stored at -20 °C until needed. The following steps were performed on ice or at 4 °C. The cell pellet(s) were resuspended (10% w/v) in ice-cold Buffer A and lysed by sonication (20 second pulse, 40 second cooldown, 10-15 cycles). The lysate was pelleted by high-speed centrifugation using a Fiberlite F15-8 x 50cy fixed angle rotor (12000 rpm, 45-60 minutes, 4 °C). The cell-free extract was collected and clarified by filtration (Millex-HP 0.45 μm polyethersulfone, Merck). All His-tagged proteins (excluding *Bs*Sfp) were isolated from the cell-free extract using a HisTrap HP (1 mL) Ni-IMAC column. The bound His-tagged protein was washed with

Buffer B (10 mL) and eluted with Buffer C into fractions containing Buffer D (1 mL). Fractions containing the desired protein were pooled, reduced by centrifugal concentration (< 1 mL) and further purified/exchanged into Buffer D using SEC (hardware specified in table S2). Fractions containing purified protein were pooled, concentrated and used within 1-2 days. His-tagged *Bs*Sfp was purified using a HiTrap TALON Crude (1 mL) Co-IMAC column, washed with Buffer E and eluted with Buffer C (10 mL) into fractions containing Buffer D (1 mL). Fractions containing *Bs*Sfp were dialysed in Buffer D (2-3 L) overnight at 4 °C with gentle magnetic stirring. Stocks of dialysed *Bs*Sfp could be safely flash-frozen in glycerol (10% v/v) and stored at -80 °C.

Creation of pET28a-*Mt*PKS12 [KRS$_1$][ER$_1$][KRC$_1$] (pIES02). A PCR reaction mixture including template DNA (10-100 ng), equimolar dNTPs (200 μM), forward and reverse primers (0.5 μM) and Phusion HF DNA polymerase (1 unit) was prepared in Phusion GC buffer (1X), DMSO (3% v/v) and nuclease-free water for a total reaction volume of 50 μL. Following an initial denaturation step (98 °C, 30 s), the DNA was amplified over 30 cycles of denaturation (98 °C, 10 s), annealing (60 °C, 30 s) and extension (72 °C, 30 s kb^{-1}), followed by a final extension step lasting 10 minutes. The desired amplicon was identified by agarose gel electrophoresis, excised and purified by gel extraction. A Gibson Assembly reaction mixture (20 μL total volume) containing NEB Gibson Assembly Master Mix (1X), a 5-fold excess of insert-to-vector, and nuclease-free water was incubated at 50 °C for 1 hour. The mixture was diluted 4-fold in nuclease-free water prior to heat-shock transformation using chemically-competent NEB 5-alpha cells (New England Biolabs). Successful transformants were selected on LB-agar plates supplemented with 50 μg mL^{-1} kanamycin, and the transformant colonies were allowed to develop overnight at 37 °C. Several colonies were subcultured at 37 °C overnight in LB media (5 mL) with 50 μg mL^{-1} kanamycin. The cells were harvested and plasmids were purified in nuclease-free water (50 μL) by miniprep. Positive clones were confirmed by restriction endonuclease digest and DNA sequencing.

Monitoring Ketoreduction by Spectrophotometry. Freshly-purified *Mt*PKS12 [KR_{S1}][ER_1][KR_{C1}] or [M_1*] (10 μM) was incubated with ketone **6** (0.25-32 mM) and NADPH (1 mM) in DMSO (3.2% v/v) and Buffer D in a total reaction volume of 200 μL. Enzyme-negative and ketone-negative controls were prepared by substituting these components with Buffer D and DMSO, respectively. Substrate-negative controls were prepared by the replacement of **6** with DMSO. The reaction was monitored every 30 seconds for 90 minutes in 96-well format using a BioTek Synergy HXT (30 °C, 340 nm), configured for pathlength correction. A molar attenuation coefficient of 6220 M^{-1} cm^{-1} was used to convert absorbance into concentration using Beer's law.

Monitoring Ketoreduction by GC/EI-MS. Freshly-purified *Mt*PKS12 [KR_{S1}][ER_1][KR_{C1}] (10 μM) was incubated with ketone **6** (1 mM) and NADPH (1 mM) in DMSO (3.2% v/v) and Buffer D in a total reaction volume of 100 μL. Replicate reactions were prepared for a timecourse assay (0-600 minutes). An enzyme-negative control was prepared by the replacement of enzyme with Buffer D. A substrate-negative control was prepared by the replacement of **6** with DMSO. The reactions were incubated at 30 °C with agitation. At the desired timepoint, a reaction replicate was quenched and extracted using ice-cold ultrapure EtOAc (100 μL). The organic extract was recovered by microcentrifugation (13000 rpm, 5 minutes) and analysed by GC/EI-MS using the following oven parameters: 80 °C (initial, 2 minute hold), 165 °C (15 °C min^{-1}, no hold), 200 °C (20 °C min^{-1}, no hold). A solvent delay of 6.67 minutes was used. Analytes were quantified using calibration standards prepared on the day of analysis (see also Fig. S9A-B).

Preparation and Analysis of [M_1*]-derived 2-Methyl Guerbet Acids 5a-e. [M_1*] (40 nmol), *Bs*Sfp (0.5 nmol), $MgCl_2$ (1 μmol), DTT (0.1 μmol) and CoASH (50 nmol) were incubated at room temperature for 1 hour prior to the addition of NADPH (0.2 μmol), **2a-e** (50 nmol) and **3** (50 nmol). The reaction was standardised to 100 μL by the addition of Buffer D. The final concentration of the

reaction components were as follows: [M_1*] (400 μM), *Bs*Sfp (5 μM), $MgCl_2$ (10 mM), DTT (1 mM), CoASH (500 μM), NADPH (2 mM), **2a-e** (500 μM), **3** (500 μM). An enzyme-negative control was prepared by the replacement of [M_1*] with Buffer D. A substrate-negative control was prepared by the replacement of **2a-e** and **3** with dH_2O. After overnight incubation (30 °C), the reaction(s) were quenched by the addition of concentrated NaOH (10 μL) and heated to 50 °C for 1 hour. The quenched mixtures were acidified using neat HCl (10 μL) and extracted using ice-cold ultrapure EtOAc (200 μL). The organic extract was recovered by microcentrifugation (13000 rpm, 5 minutes) and collected. This extraction step was repeated two more times. The EtOAc extracts were pooled, dried *in vacuo* using a RV2-33 CDplus rotational vacuum concentrator and re-dissolved in 20 μL EtOAc. The following oven parameters were used for GC/EI-MS: 80 °C (initial, 2 minute hold), 200 °C (15 °C min^{-1}, 10 minute hold), 280 °C (15 °C min^{-1}, 5 minute hold). A solvent delay of 3.25 minutes was used. For selective ion monitoring, the mass analyser was configured to detect the McLafferty ion (m/z = 74) and the molecular ion. **5b** crude yield: 129.5 ng (0.534 nmol).

Monitoring *Mt*PKS12 KS Thioesterase Activity by GC/EI-MS. Freshly-purified *Mt*PKS12 [KS_1][AT_1], wildtype *Mt*PKS12 [KS_2][AT_2] or *Mt*PKS12 $H_{2361}D$ [KS_2][AT_2] (10 μM) was incubated with **2b** (500 μM) in Buffer D in a total reaction volume of 100 μL. The reactions were incubated at 30 °C with agitation. At the desired timepoint, the reaction(s) were quenched and extracted using ice-cold ultrapure EtOAc (100 μL). The organic extract was recovered by microcentrifugation (13000 rpm, 5 minutes) and analysed by GC/EI-MS using the following oven parameters: 80 °C (initial, 2 minute hold), 260 °C (20 °C min^{-1}, 1 minute hold).

Monitoring *Mt*PKS12 [KS_1] Thioesterase Activity by Spectrophotometry. Freshly-purified *Mt*PKS12 [KS_1][AT_1] (5 μM) was incubated with **2a, 2b, 2d** or **2d** (250 μM) in Buffer D in a total reaction volume of 200 μL. Substrate-negative and enzyme-negative controls were prepared by substituting these components with dH_2O and Buffer D, respectively. The reactions were incubated at 30 °C for 18 hours with rigorous shaking. The reactions were diluted 2-fold in Buffer D prior to the addition of DTNB (500 μM). Absorbances were measured using a BioTek Synergy HXT (28 °C, 412 nm), configured for pathlength correction. A molar attenuation coefficient of 14150 M^{-1} cm^{-1} was used to convert absorbance into concentration using Beer's law.

Creation of *Mt*PKS12 [KS_2][AT_2] $H_{2361}D$ Mutant (pIES12). Using pET28a-[KS_2][AT_2] (pIES11) as the DNA template, the *Mt*PKS12 [KS_2][AT_2] $H_{2361}D$ mutant was prepared using a Q5 Site-Directed Mutagenesis kit exactly according to manufacturer protocol. Following mutagenesis, Kinase-Ligase-DpnI (KLD) treatment and heat-shock transformation, colonies of NEB 5-alpha transformants were developed on kanamycin (50 μg mL^{-1}) LB-agar plates. Several colonies were propagated at 37 °C overnight in LB media (5 mL) with kanamycin (50 μg mL^{-1}). The cells were harvested and plasmid minipreps were prepared in nuclease-free water (50 μL). Since the $H_{2361}D$ mutation eliminates an NcoI restriction site, positive mutant clones were identified by their resistance to restriction endonuclease digest (XbaI/NcoI/XhoI) and later confirmed by DNA sequencing.

[M_1*] + *Mt*PKS12 [KS_2][AT_2] Assay. [M_1*] (1 nmol), *Bs*Sfp (0.2 nmol), $MgCl_2$ (1 μmol), DTT (0.1 μmol) and CoASH (25 nmol) were incubated at room temperature for 1 hour prior to the addition of *Mt*PKS12 [KS_2][AT_2] (0.5-2 nmol), NADPH (0.4 μmol), C_{12}-CoA (0.1 μmol) and 2-MMal-CoA (0.1 μmol). The reactions were standardised to 100 μL by the addition of Buffer D. The final concentrations of the reaction components were as follows: [M_1*] (10 μM), *Bs*Sfp (2 μM), $MgCl_2$ (10 mM), DTT (1 mM), CoASH (250 μM), [KS_2][AT_2] (5-20 μM), NADPH (2 mM), C_{12}-CoA (500 μM), 2-MMal-CoA (500 μM). Enzyme-negative controls were prepared by the replacement of [M_1*] or [KS_2][AT_2] with Buffer D. A substrate-negative control was prepared by the replacement of **2b** and **3** with dH_2O. The reactions were incubated at 30 °C overnight with agitation. The reaction(s) were quenched and extracted using ice-cold EtOAc (100 μL). The organic extract was recovered by microcentrifugation (13000 rpm,

5 minutes) and analysed by GC/EI-MS using the following oven parameters: 80 °C (initial, 2 minute hold), 200 °C (15 °C min^{-1}, 10 minute hold), 280 °C (15 °C min^{-1}, 5 minute hold). A solvent delay of 3.25 minutes was used. For selective ion monitoring, the mass analyser was configured to detect the McLafferty ion (m/z = 74).

Spectrophotometric detection of *Ec*FadD activity using ammonium molybdate. The ammonium molybdate assay was based adapted from Johann E. Kufs *et al.*[13] In brief, a reaction mixture (1 mL) containing purified *Ec*FadD (1 µM), **1b** (1 mM), DTT (1 mM), CoASH (250 µM), ATP (1 mM) and DMSO (0.1% v/v) was prepared in Buffer D. An enzyme-negative control was prepared by the replacement of *Ec*FadD with Buffer D. A **1b**-negative control was prepared by the replacement of 1b with DMSO. The reactions were incubated at 30 °C for 1 hour with rigorous agitation. The reactions were quenched by the addition of a stopping solution (140 µL) containing ammonium molybdate (2.5% w/v) and H_2SO_4 (0.5 N). Following the addition of β-mercaptoethanol solution (0.5 M, 140 µL), the quenched reactions were incubated for 10 minutes at room temperature. Any precipitate was pelleted by microcentrifugation (13000 rpm, 1 minute) and the supernatant was analysed using a pre-blanked spectrophotometer (400-700 nm). The presence of PPi results in a colour change from yellow to blue under reducing conditions (λ_{max} = 580 nm).

***Ec*FadD + [M_1*] Coupled Assay.** A reaction mixture (682.5 µL) containing [M_1*] (11.6 µM), *Mt*PKS12 [KS_2][AT_2] (5.4 µM), BsSfp (1.5 µM) *Ec*FadD (0.8 µM), $MgCl_2$ (7.33 mM), **1b** (733 µM), DTT (733 µM), CoASH (183 µM), ATP (1.83 mM), **3** (366 µM) and DMSO (0.07% v/v) was prepared in Buffer D. An enzyme-negative control was prepared by the replacement of *Ec*FadD with Buffer D. A **1b**-negative control was prepared by the replacement of **1b** with DMSO. Following overnight incubation (30 °C), the reaction(s) were quenched by the addition of concentrated NaOH (68 µL) and heated to 50 °C for 1 hour. The quenched mixtures were acidified using neat HCl (68 µL) and extracted using ice-cold ultrapure EtOAc (700 µL). The organic extract was recovered by microcentrifugation (13000 rpm, 5 minutes) and collected. This extraction step was repeated once more. The EtOAc extracts were pooled, dried *in vacuo* using a RV2-33 CDplus rotational vacuum concentrator and re-dissolved in 30 µL EtOAc. The following oven parameters were used for GC/EI-MS: 80 °C (initial, 2 minute hold), 200 °C (15 °C min^{-1}, 10 minute hold), 280 °C (15 °C min^{-1}, 5 minute hold). A solvent delay of 3.25 minutes was used. For selective ion monitoring, the mass analyser was configured to detect the McLafferty ion (m/z = 74) and the molecular ion (m/z = 242). **5b** crude yield: 84.9 ng (0.350 nmol)

Supplementary Tables

Table S1. Summary of buffers, rich media and reagent stocks.

Item	Contents
**Buffer A**	50 mM HEPES (pH 7.5), 200 mM NaCl, 20 mM imidazole
**Buffer B**	50 mM HEPES (pH 7.5), 200 mM NaCl, 40 mM imidazole
**Buffer C**	50 mM HEPES (pH 7.5), 200 mM NaCl, 500 mM imidazole
**Buffer D**	50 mM HEPES (pH 7.5), 200 mM NaCl
**Buffer E**	50 mM HEPES (pH 7.5), 200 mM NaCl, 30 mM imidazole
**LB media**	Yeast extract (5 g L^{-1}), tryptone (10 g L^{-1}), NaCl (10 g L^{-1})
**LB-Agar**	Yeast extract (5 g L^{-1}), tryptone (10 g L^{-1}), NaCl (10 g L^{-1}), Agar (15 g L^{-1})
**Kanamycin (1000X)**	Kanamycin sulfate (50 mg mL^{-1}) in dH_2O
**Chloramphenicol (1000X)**	Chloramphenicol (25 mg mL^{-1}) in EtOH
**1 M IPTG (1000X)**	IPTG (238 mg mL^{-1}) in dH_2O
**DTT (100X)**	DTT (15.4 mg mL^{-1}) in dH_2O
trans*-1-decalone Master	*trans*-1-decalone (1 M) in DMSO
**25 mM DTNB (50X)**	DTNB (9.91 mg mL^{-1}) in EtOH

*For [KR_{S1}][ER_1][KR_{C1}] kinetics, serially-diluted DMSO stocks of *trans*-1-decalone were prepared from the master DMSO stock.

Table S2. Plasmid table and purification summary.

Internal Name	Product	Vector	Selection	IMAC Purification	SEC Purification
**Sfp-pACYC**	*Bs*Sfp	pACYC-Duet	Chloramphenicol	HiTrap TALON Crude (1 mL)	N/A
**pISL52**	[M_1*]	pET26b	Kanamycin	HisTrap HP (1 mL)	HiLoad 16/600 Superose 6 pg (120 mL)
**pIES01**	*Mt*PKS12 [ACP_1]	pET28a	Kanamycin	HisTrap HP (1 mL)	HiLoad 16/600 Superdex 75 pg (120 mL)
**pIES02**	*Mt*PKS12 [KR_{S1}][ER_1][KR_{C1}]	pET28a	Kanamycin	HisTrap HP (1 mL)	HiLoad 16/600 Superdex 200 pg (120 mL)
**pIES03**	*Mt*PKS12 [KS_1][AT_1]	pET28a	Kanamycin	HisTrap HP (1 mL)	HiLoad 16/600 Superdex 200 pg (120 mL)

**pIES11**	*Mt*[KS_2][AT_2] (wildtype)	pET28a	Kanamycin	HisTrap HP (1 mL)	HiLoad 16/600 Superdex 200 pg (120 mL)
**pIES12**	*Mt*[KS_2][AT_2] ($H_{305}D$)	pET28a	Kanamycin	HisTrap HP (1 mL)	HiLoad 16/600 Superdex 200 pg (120 mL)
**pET28a-*fadD***	*Ec*FadD	pET28a	Kanamycin	HisTrap HP (1 mL)	N/A

Table S3. Primers used for subcloning *Mt*PKS12 [KR_{S1}][ER_1][KR_{C1}] (pIES02).

Internal Name	Gene Origin	Insert Primer Sequence (5'→ 3')	Destination Vector	Destination Vector Primer Sequence (5'→ 3')
**pIES02**	pISL52	**Forward:** GGGTCGCGGATCCGAGCGGCAGCTGCTGG<br>**Reverse:** GGAGCTCGAATTCTTACAGCCCGTGCAGGCG	pET28a (empty)	**Forward:** CGGGCTGTAAGAATTCGAGCTCCGTCG<br>**Reverse:** GCCGCTCGGATCCGCGACCCATTTGC

Table S4. Primers used for *Mt*PKS12 [KS_2][AT_2] mutagenesis.

Internal Name	Gene Origin	Mutagenesis Primer Sequence (5'→ 3')
pIES12	pIES11	**Forward:** GTTGTGGAGGGCGATGGTACAGGC<br>**Reverse:** GTCAACCTCTGCCGCGCTCAG

Table S5. Domain annotation for [M_1*].

Domain	Start Position	End Position	Key Catalytic/Supporting Residues
[DD_1]	Met_1	Glu_{35}	
[KS_1]	Pro_{36}	Val_{460}	Cys_{203}, His_{338}, His_{379}
[AT_1]	Lys_{557}	G_{889}	Gly_{648}, His_{649}, Ser_{650}, Gln_{651}, Gly_{652}, Tyr_{750}, Ala_{751}, Ser_{752}, His_{753}
[DH_1]	His_{926}	Thr_{1190}	H_{558}, D_{1120}
[KR_{S1}]	Glu_{1191}	Ala_{1348}	
[ER_1]	Pro_{1360}	Ala_{1678}	Gly_{1503}, Gly_{1504}, Val_{1505}, Gly_{1506}, Met_{1507}, Ala_{1508}
[KR_{C1}]	Gly_{1679}	Arg_{1907}	Thr_{1685}, Gly_{1686}, Gly_{1687}, Thr_{1688}, Gly_{1689}, Met_{1690}, Ala_{1891}, Gly_{1892}, Leu_{1771}, Asp_{1772}, Asp_{1773}, Ser_{1815}, Tyr_{1828}
[ACP_1]	Pro_{1957}	Gly_{2040}	Ser_{1988}
[DD_2]	Ser_{2041}	Glu_{2119}	

Table S6. Trypsin digest MS results for [M1*] (estimated MW = 222288 Da, calculated pI = 5.10).

Start	End	Expected MW	Calculated MW	E	Peptide ID
1	13	1510.7417	1510.7562	3.00E-05	-.MVDQLQHATEALR.K
1	13	1510.7489	1510.7562	2.00E-05	-.MVDQLQHATEALR.K
1	13	1526.7406	1526.7511	3.40E-05	-.MVDQLQHATEALR.K+ Oxidation (M)
1	14	1638.837	1638.8512	3.50E-08	-.MVDQLQHATEALRK.A
1	14	1638.8413	1638.8512	3.40E-05	-.MVDQLQHATEALRK.A
1	14	1654.8281	1654.8461	8.20E-08	-.MVDQLQHATEALRK.A+ Oxidation (M)
2	13	1379.7061	1379.7157	4.90E-05	M.VDQLQHATEALR.K
14	21	941.5611	941.5658	6.50E-07	R.KALVQVER.L
15	21	813.4668	813.4708	2.40E-05	K.ALVQVER.L
33	45	1405.6572	1405.6694	4.20E-10	R.SSEPIAIVGMSCR.F
33	45	1421.6504	1421.6643	3.80E-09	R.SSEPIAIVGMSCR.F+ Oxidation (M)
46	64	2030.9407	2030.952	1.50E-13	R.FPGGVDSPEGLWQMVADAR.D
46	64	2046.929	2046.9469	2.40E-06	R.FPGGVDSPEGLWQMVADAR.D+ Oxidation (M)
65	74	1195.5076	1195.5179	2.20E-05	R.DVMSEFPTDR.G
65	74	1211.5011	1211.5129	5.80E-06	R.DVMSEFPTDR.G+ Oxidation (M)
65	74	1211.5054	1211.5129	3.60E-07	R.DVMSEFPTDR.G+ Oxidation (M)
65	89	2849.2743	2849.2967	1.30E-06	R.DVMSEFPTDRGWDLAGLFDPDPDVR.H
65	89	2865.2688	2865.2916	2.80E-08	R.DVMSEFPTDRGWDLAGLFDPDPDVR.H+ Oxidation (M)
75	89	1671.7757	1671.7893	7.00E-12	R.GWDLAGLFDPDPDVR.H
96	126	3250.48	3250.503	2.20E-14	R.TGGFVDGVADFDPAFFGISPSEALAMDPQHR.M
96	126	3266.4775	3266.4979	6.60E-14	R.TGGFVDGVADFDPAFFGISPSEALAMDPQHR.M+ Oxidation (M)
127	138	1488.7534	1488.7646	2.40E-06	R.MLLELSWEALER.A
127	138	1488.7569	1488.7646	1.40E-07	R.MLLELSWEALER.A
127	138	1504.7452	1504.7595	3.20E-10	R.MLLELSWEALER.A+ Oxidation (M)
127	138	1504.7514	1504.7595	8.00E-08	R.MLLELSWEALER.A+ Oxidation (M)
139	147	898.4825	898.4872	6.80E-08	R.AGIDPTGLR.G
174	185	1165.567	1165.5761	9.40E-10	R.LTGMTSSVASGR.V
174	185	1181.5629	1181.571	2.30E-08	R.LTGMTSSVASGR.V+ Oxidation (M)
186	218	3328.7157	3328.7159	5.40E-11	R.VAYVLGLEGPAVSVDTACSSSLVALHMAVGSLR.S
219	244	2696.3037	2696.3116	4.30E-12	R.SGECDLALAGGVTVNATPTVFVEFSR.H
245	253	977.5104	977.5155	0.0087	R.HRGLAPDGR.C
261	277	1730.8261	1730.841	3.70E-14	R.ADGVGWSEGGGMLVLQR.L
261	277	1746.8211	1746.8359	2.60E-12	R.ADGVGWSEGGGMLVLQR.L+ Oxidation (M)
261	277	1746.8252	1746.8359	3.90E-09	R.ADGVGWSEGGGMLVLQR.L+ Oxidation (M)
283	316	3365.7132	3365.7553	5.50E-15	R.RLGHPVLAVVVGSAVNQDGASNGLTAPNGPSQQR.V
284	316	3209.6297	3209.6542	6.20E-15	R.LGHPVLAVVVGSAVNQDGASNGLTAPNGPSQQR.V
284	316	3209.6627	3209.6542	1.80E-20	R.LGHPVLAVVVGSAVNQDGASNGLTAPNGPSQQR.V
320	361	4122.0169	4122.0505	3.00E-07	R.AALANAGLSAAEVDVVEGHGTGTTLGDPIEAQALLATYGQDR.G
320	361	4122.0327	4122.0505	7.70E-05	R.AALANAGLSAAEVDVVEGHGTGTTLGDPIEAQALLATYGQDR.G
362	374	1367.6978	1367.7085	9.10E-08	R.GEPGEPLWLGSVK.S
375	391	1610.8027	1610.8199	1.20E-05	K.SNMGHTQAAAGVAGVIK.M
375	391	1610.8058	1610.8199	3.30E-12	K.SNMGHTQAAAGVAGVIK.M
375	391	1610.8095	1610.8199	1.30E-08	K.SNMGHTQAAAGVAGVIK.M
375	391	1626.801	1626.8148	3.80E-11	K.SNMGHTQAAAGVAGVIK.M+ Oxidation (M)
398	428	3326.7199	3326.7412	3.30E-05	R.HELLPATLHVDVPSPHVDWSAGAVELLTAPR.V
438	463	2632.4265	2632.4449	1.10E-09	R.RAGVSSFGISGTNAHVIIEAVPVVPR.R
439	463	2476.3275	2476.3438	9.70E-18	R.AGVSSFGISGTNAHVIIEAVPVVPR.R
439	463	2476.3313	2476.3438	2.20E-05	R.AGVSSFGISGTNAHVIIEAVPVVPR.R
464	480	1807.961	1807.9733	9.60E-08	R.REAGWAGPVVPWVVSAK.S
465	480	1651.8588	1651.8722	4.20E-08	R.EAGWAGPVVPWVVSAK.S
465	480	1651.8589	1651.8722	1.30E-06	R.EAGWAGPVVPWVVSAK.S
481	491	1144.5883	1144.5948	0.00025	K.SESALRGQAAR.L
492	514	2375.1642	2375.187	1.70E-10	R.LAAYVRGDDGLDVADVGWSLAGR.S
498	514	1701.7801	1701.7959	9.40E-15	R.GDDGLDVADVGWSLAGR.S
521	530	1042.5447	1042.552	0.0012	R.AVVVGGDRDR.L
531	549	1882	1882.016	1.90E-14	R.LLAGLDELAGDQLGGSVVR.G
608	617	909.4625	909.4668	5.30E-06	R.GAPGAPGLDR.V
638	666	2858.4806	2858.5039	6.60E-05	K.SVAVHPDAVIGHSQGEIAAAYVAGALSLR.D
638	666	2858.4858	2858.5039	9.40E-10	K.SVAVHPDAVIGHSQGEIAAAYVAGALSLR.D
676	699	2315.1378	2315.1726	1.70E-12	R.SKLLAGLAGPGGMVSIACGADQAR.D+ Oxidation (M)
678	699	2084.0334	2084.0507	4.60E-14	K.LLAGLAGPGGMVSIACGADQAR.D
678	699	2100.0322	2100.0456	2.00E-13	K.LLAGLAGPGGMVSIACGADQAR.D+ Oxidation (M)
678	699	2100.0322	2100.0456	1.50E-08	K.LLAGLAGPGGMVSIACGADQAR.D+ Oxidation (M)
700	708	1002.5039	1002.5134	0.031	R.DLLAPFGDR.V
700	708	1002.5066	1002.5134	2.30E-05	R.DLLAPFGDR.V
745	761	1971.992	1972.0014	1.70E-11	R.RIEVDYASHSVEVEAIR.G
746	761	1815.8728	1815.9003	2.90E-16	R.IEVDYASHSVEVEAIR.G
746	774	3106.5717	3106.5934	5.60E-06	R.IEVDYASHSVEVEAIRGPLAEALSGIEPR.S
762	774	1308.6941	1308.7037	6.30E-09	R.GPLAEALSGIEPR.S
778	788	1227.6154	1227.6248	2.90E-07	R.TVFFSTVTGNR.L
789	801	1557.6973	1557.71	3.50E-13	R.LDTAGLDADYWYR.N
885	902	1915.9053	1915.9204	4.10E-15	R.GTLDGAGYVELPTYAFDK.R
885	903	2072.0092	2072.0215	1.30E-09	R.GTLDGAGYVELPTYAFDKR.R
1085	1092	904.4523	904.4589	0.00013	R.GLTAMWAR.G
1085	1092	920.4472	920.4538	1.50E-05	R.GLTAMWAR.G+ Oxidation (M)
1093	1101	1048.5135	1048.5189	2.50E-06	R.GEEIFAEVR.L
1158	1192	3444.845	3444.865	2.00E-05	R.IAPAGPSAVSVELADGLGLPVLSVASMVARPVTER.Q+ Oxidation (M)

1193	1205	1269.6581	1269.6677	8.60E-10	R.QLLAAVSGSGPDR.L
1246	1270	2674.3645	2674.3827	2.00E-10	R.SHQALAAVQSWLTDHESGVLVVATR.G
1271	1293	2337.2091	2337.2263	3.60E-09	R.GAMALPREDVADLAGAAVWGLVR.S
1271	1293	2353.2039	2353.2212	2.70E-05	R.GAMALPREDVADLAGAAVWGLVR.S+ Oxidation (M)
1278	1293	1640.8428	1640.8522	1.80E-11	R.EDVADLAGAAVWGLVR.S
1303	1331	2924.4949	2924.5165	5.10E-14	R.IVLVDSDAATDDAAIAMALATGEPQVVLR.G
1303	1331	2940.4885	2940.5114	4.20E-10	R.IVLVDSDAATDDAAIAMALATGEPQVVLR.G+ Oxidation (M)
1332	1339	850.4245	850.4297	2.70E-06	R.GGQVYTAR.V
1345	1359	1533.7825	1533.794	3.50E-08	R.AADAILVPPGDGPWR.L
1360	1372	1333.6909	1333.699	6.30E-11	R.LGLGSAGTFENLR.L
1360	1372	1333.6931	1333.699	1.70E-07	R.LGLGSAGTFENLR.L
1360	1389	3044.5641	3044.6043	4.20E-07	R.LGLGSAGTFENLRLEPVPNADAPLGPGQVR.V
1373	1389	1728.8955	1728.9159	5.80E-07	R.LEPVPNADAPLGPGQVR.V
1373	1389	1728.901	1728.9159	2.60E-11	R.LEPVPNADAPLGPGQVR.V
1373	1389	1728.9063	1728.9159	8.40E-09	R.LEPVPNADAPLGPGQVR.V
1496	1514	1820.0005	1820.0091	2.80E-06	R.VLIHAGTGGVGMAAVQLAR.H
1496	1514	1835.9939	1836.004	1.30E-09	R.VLIHAGTGGVGMAAVQLAR.H+ Oxidation (M)
1515	1526	1271.6779	1271.6874	1.30E-08	R.HLGLEVFATASK.G
1527	1533	874.4616	874.4661	0.0012	K.GKWDTLR.A
1534	1546	1464.5852	1464.594	1.20E-08	R.AMGFDDDHISDSR.S
1534	1546	1480.5803	1480.5889	5.00E-10	R.AMGFDDDHISDSR.S+ Oxidation (M)
1547	1553	866.3976	866.4021	0.00012	R.SLEFEDK.F
1547	1555	1169.5638	1169.5717	1.10E-07	R.SLEFEDKFR.A
1562	1580	2008.9965	2009.0106	1.30E-10	R.GFDVVLDSLAGEFVDASLR.L
1581	1593	1316.7055	1316.7162	1.50E-07	R.LVAPGGVFLEMGK.T
1581	1593	1332.6964	1332.7112	7.10E-08	R.LVAPGGVFLEMGK.T+ Oxidation (M)
1594	1610	1883.9786	1883.9854	8.60E-06	K.TDIRDPGVIAQQYPGVR.Y
1598	1610	1398.7145	1398.7256	5.00E-06	R.DPGVIAQQYPGVR.Y
1611	1623	1622.8213	1622.8317	0.00093	R.YRAFDLFEPGRPR.M
1613	1623	1303.6619	1303.6673	0.00082	R.AFDLFEPGRPR.M
1624	1651	3250.62	3250.6519	9.10E-06	R.MHQYMLELATLFGDGVLRPLPVTTFDVR.R+ 2 Oxidation (M)
1669	1697	2760.377	2760.4125	7.80E-12	K.VVMLMPGSWAAGTVLITGGTGMAGSAVAR.H
1669	1697	2776.3938	2776.4074	3.90E-12	K.VVMLMPGSWAAGTVLITGGTGMAGSAVAR.H+ Oxidation (M)
1669	1697	2792.3796	2792.4024	1.70E-11	K.VVMLMPGSWAAGTVLITGGTGMAGSAVAR.H+ 2 Oxidation (M)
1669	1697	2808.3659	2808.3973	2.00E-11	K.VVMLMPGSWAAGTVLITGGTGMAGSAVAR.H+ 3 Oxidation (M)
1707	1713	799.4879	799.4916	4.40E-06	R.NLVLVSR.R
1714	1745	3118.5223	3118.5465	1.50E-13	R.RGPDAPGAAELVAELAAAGAQVQVVACDAADR.A
1751	1783	3422.7382	3422.7868	1.70E-07	K.VIADIPVQHPLSGVIHTAGALDDAVVMSLTPDR.V+ Oxidation (M)
1751	1789	4088.2003	4088.2093	0.011	K.VIADIPVQHPLSGVIHTAGALDDAVVMSLTPDRVDVVLR.S
1790	1803	1661.8556	1661.8638	1.60E-07	R.SKVDAAWHLHELTR.D
1790	1803	1661.8573	1661.8638	6.20E-06	R.SKVDAAWHLHELTR.D
1792	1803	1446.7279	1446.7368	3.80E-05	K.VDAAWHLHELTR.D
1792	1803	1446.728	1446.7368	0.00017	K.VDAAWHLHELTR.D
1843	1874	3292.5827	3292.6411	1.10E-14	R.RAHGLPAISLGWGLWDQASAMTGGLDAADLAR.L+ Oxidation (M)
1843	1874	3292.6083	3292.6411	1.30E-12	R.RAHGLPAISLGWGLWDQASAMTGGLDAADLAR.L+ Oxidation (M)
1844	1874	3136.5149	3136.54	2.30E-16	R.AHGLPAISLGWGLWDQASAMTGGLDAADLAR.L+ Oxidation (M)
1844	1874	3136.5163	3136.54	2.30E-08	R.AHGLPAISLGWGLWDQASAMTGGLDAADLAR.L+ Oxidation (M)
1908	1914	800.4713	800.4756	5.80E-05	R.IDLTALR.A
1915	1933	1936.971	1936.9829	3.00E-08	R.AHAVAVPPMFSDLASAPTR.R
1915	1933	1952.9614	1952.9778	3.60E-10	R.AHAVAVPPMFSDLASAPTR.R+ Oxidation (M)
1915	1934	2109.0653	2109.0789	0.00032	R.AHAVAVPPMFSDLASAPTRR.Q+ Oxidation (M)
1915	1934	2109.0683	2109.0789	0.0032	R.AHAVAVPPMFSDLASAPTRR.Q+ Oxidation (M)
1934	1944	1158.588	1158.5993	3.50E-06	R.RQVDDSVAAAK.S
1935	1944	1002.4923	1002.4982	7.20E-08	R.QVDDSVAAAK.S
1953	1970	1951.0894	1951.1003	1.30E-09	R.LHGLPEAEQHAVLLGLVR.L
1971	1989	2016.0762	2016.0891	1.90E-10	R.LHIATVLGNITPEAIDPDK.A
1990	2005	1812.8675	1812.8716	4.40E-08	K.AFQELGFDSLTAVEMR.N
2010	2035	2699.3445	2699.3629	9.30E-18	K.SATGLALSPTLIFDYPNSAALAGYMR.R
2010	2035	2715.3388	2715.3578	7.60E-11	K.SATGLALSPTLIFDYPNSAALAGYMR.R+ Oxidation (M)
2036	2058	2384.1821	2384.1931	8.20E-15	R.RELLGSSPQDTSAVAAGEAELQR.I
2059	2066	825.5255	825.5324	1.90E-06	R.IVASIPVK.R
2059	2067	981.6271	981.6335	3.70E-06	R.IVASIPVKR.L

Table S7. NativeMark/NativePAGE calibration used to estimate [M_1*] MW by densitometry.

Standard	MW / kDa	log MW	Retention Factor
IgM Pentamer	1048	3.02	0.05
Apoferritin band 1	720	2.86	0.18
Apoferritin band 2	480	2.68	0.33
B-phycoerythrin	242	2.38	0.51
Lactate Dehydrogenase	146	2.16	0.69
Bovine Serum Albumin	66	1.82	0.86

Table S8. HiLoad 16/600 Superdex 200 (120 mL) SEC calibration used to estimate native protein MW.

Standard	V_e	Vol_0	Vol_t	V_e/V_0	MW / kDa	MW / Da	K_{av}	Lg (Da)
Ovalbumin	78	44	120	1.772727	43	43000	0.447	4.633
Conalbumin	73	44	120	1.659091	75	75000	0.382	4.875
Aldolase	64	44	120	1.454545	158	158000	0.263	5.199
Ferritin	47	44	120	1.068182	440	440000	0.0395	5.643
Thyroglobulin	45	44	120	1.022727	669	669000	0.0132	5.825

Table S9. Michaelis-Menten kinetics for the ketoreduction of **6** using *Mt*PKS12 [KR_{S1}][ER_1][KR_{C1}] and [M_1*] (10 mM), reported to 3 significant figures. Reactions were performed in technical triplicate. k_{cat}/K_M was calculated using the standard propagation of error.

Protein	V_{max} / µM min^{-1}	K_M / µM	k_{cat} / s^{-1}	k_{cat}/K_M / M^{-1} s^{-1}
[KRS_1][ER_1][KR_{C1}]	27.7 ± 1.66	1.55 x 10^3 ± 3.33 x 10^2	4.62 x10^{-2} ± 2.77 x 10^{-3}	29.8 ± 6.64
[M_1*]	39.8 ± 1.82	2.19 x 10^3 ± 3.12 x 10^2	6.64 x10^{-2} ± 3.03 x 10^{-3}	30.2 ± 4.54

Table S10. LC/ESI-MS analysis of *Bs*Sfp and *Mt*PKS12 [ACP_1].

Protein	Theoretical MW / Da	Observed MW / Da	Formula
*Bs*Sfp	28765.32	28814.33 ± 0.11	$C_{1283}H_{1943}N_{351}O_{383}S_{11}Mg_2$
*Mt*PKS12 *apo*-[ACP1]	13260.06	13284.72 ± 0.02	$C_{589}H_{935}N_{171}O_{174}S_2Mg$
*Mt*PKS12 *holo*-[ACP1]	13600.39	132624.53 ± 0.06	$C_{600}H_{956}N_{173}O_{180}S_3PMg$

Table S11. Calibration data for **5b** (2-methyltetradecanoic acid).

Dilution Factor	[5b] / µM	Mean Area	Standard Deviation
1	2231.66	7.75E+06	8.73E+01
2	1115.83	3.29E+06	3.08E+01
4	557.91	1.47E+06	1.55E+01
8	278.96	6.37E+05	3.28E+01
16	139.48	2.56E+05	4.38E+01
32	69.74	9.52E+04	3.04E+01
64	34.87	3.57E+04	1.69E+01
128	17.43	1.34E+04	2.04E+01
256	8.72	5.60E+03	1.61E+01
512	4.36	2.41E+03	3.55E+01
1024	2.18	1.14E+03	7.53E+01
2048	1.09	4.51E+03	7.60E+01
4096	0.54	2.80E+02	1.44E+02

Table S12. [KS_1][AT_1] TE specific activity calculations.

Substrate	TNB^{2-} / nmol	nmol (TNB^{2-}) min^{-1}	nmol (TNB^{2-}) min^{-1} mg^{-1}
**2a**	26.8 ± 1.30	0.0247 ± 1.20 $x10^{-3}$	0.253 ± 0.0122
**2b**	34.3 ± 1.71	0.0317 ± 1.57 $x10^{-3}$	0.323 ± 0.0161
**2d**	33.0 ± 3.05	0.0305 ± 2.81 $x10^{-3}$	0.311 ± 0.0287
**2e**	38.3 ± 1.99	0.0354 ± 1.84 $x10^{-3}$	0.361 ± 0.0187

Supplementary Figures

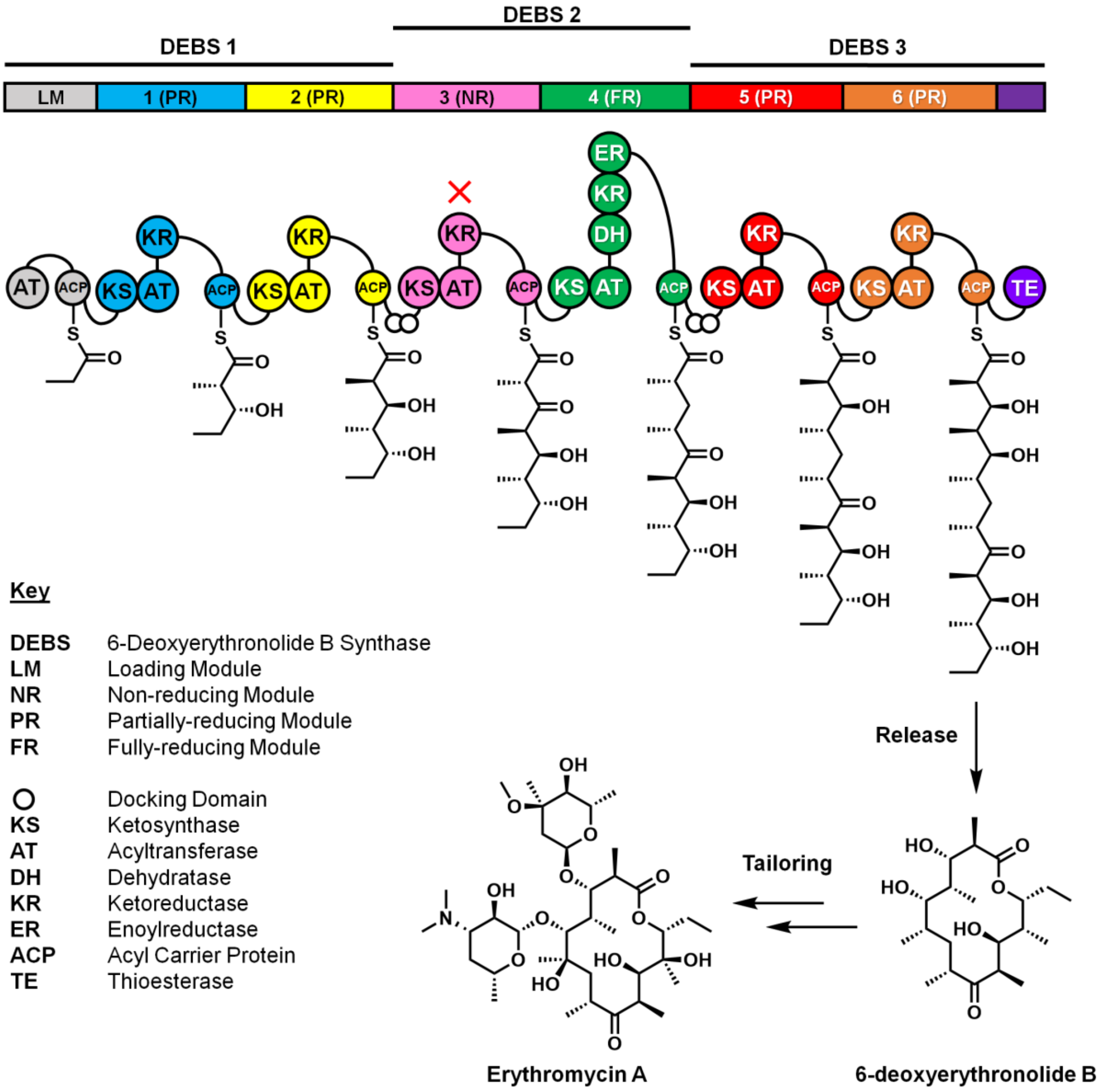

Fig. S1. 2D representation of DEBS, the archetypal multi-modular PKS. Modules 1, 2, 5 and 6 produce partially-reduced intermediates, whereas module 3 does not exhibit any reducing capability at all due to a naturally non-reducing KR (denoted with a red cross). Conversely, module 4 possesses the full arsenal of reductive domains to yield a saturated bond between C7 and C8. The thioesterase (TE) domain mediates the intramolecular cyclisation of the polyketide to furnish a C14 macrolactone, 6-deoxyerythronolide B. The macrolactone undergoes further tailoring reactions (glycosyation and oxidation) to yield the bioactive molecule erythromycin A.

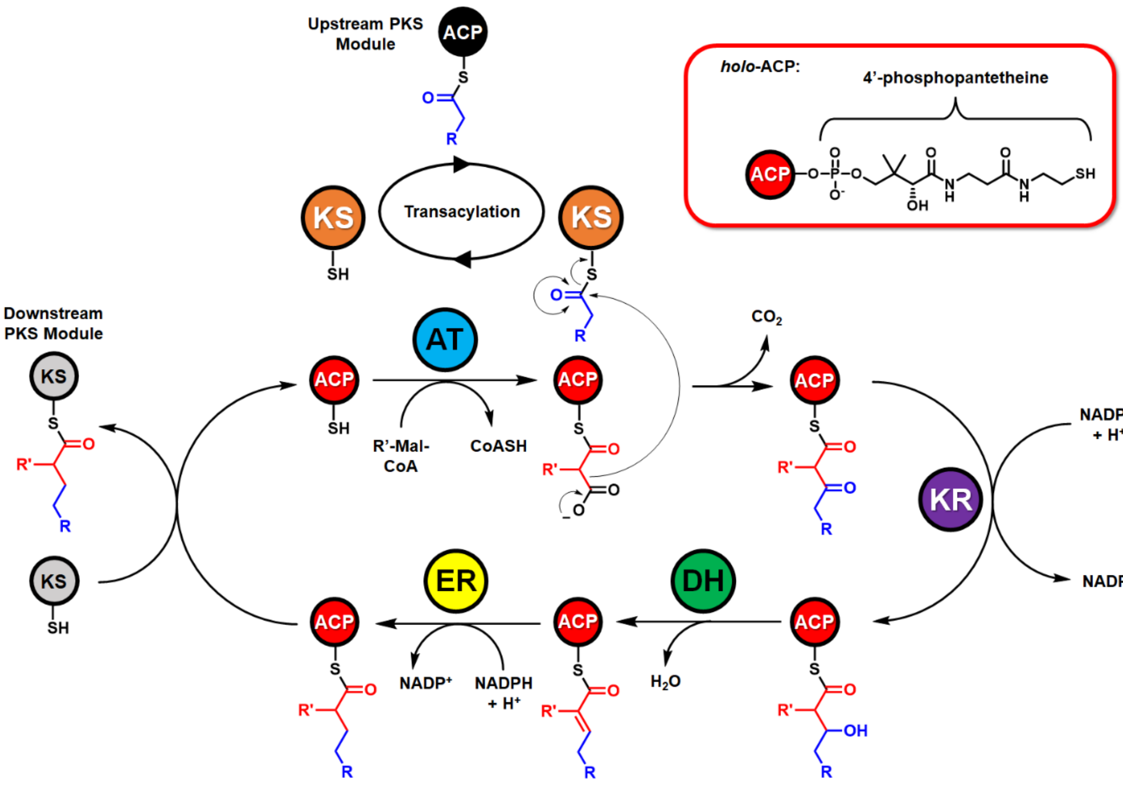

Fig. S2. Catalytic cycle of a fully-reducing PKS module embedded in a multi-modular PKS. Intermediates are loaded onto the KS domain by the upstream ACP, condensed with the extender unit and processed to yield a saturated β-carbon. The new intermediate is then transferred to the KS of the adjacent downstream module.

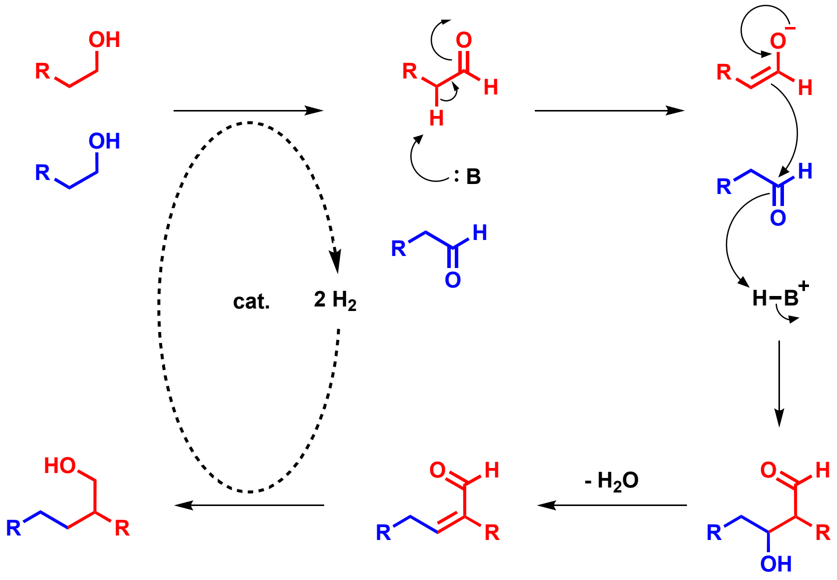

Fig. S3. The preparation of 2,2-dialkylethanols (Guerbet alcohols) via the Guerbet reaction. The Guerbet reaction proceeds at high temperatures with an excess of base and a co-catalyst.

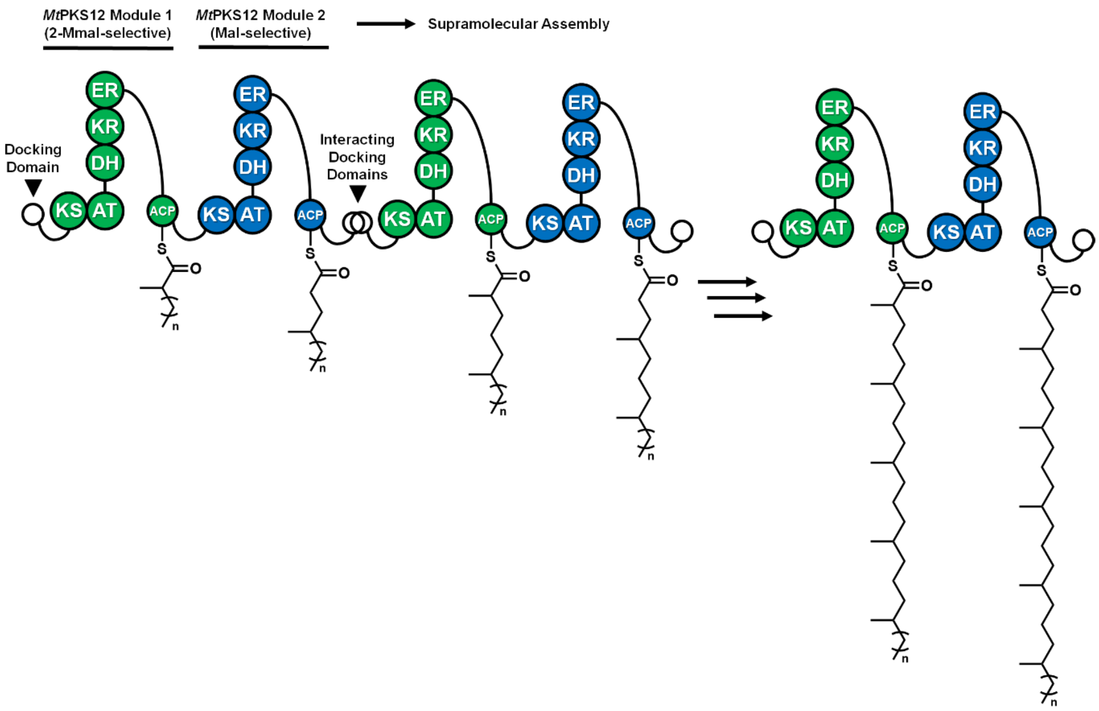

Fig. S4. The "modularly-iterative" catalysis of *Mt*PKS12. *Mt*PKS12 is a fully-reducing bimodule with N- and C-terminal docking domains. Supramolecular complexes are formed via interactions between these docking domains across multiple copies of *Mt*PKS12.

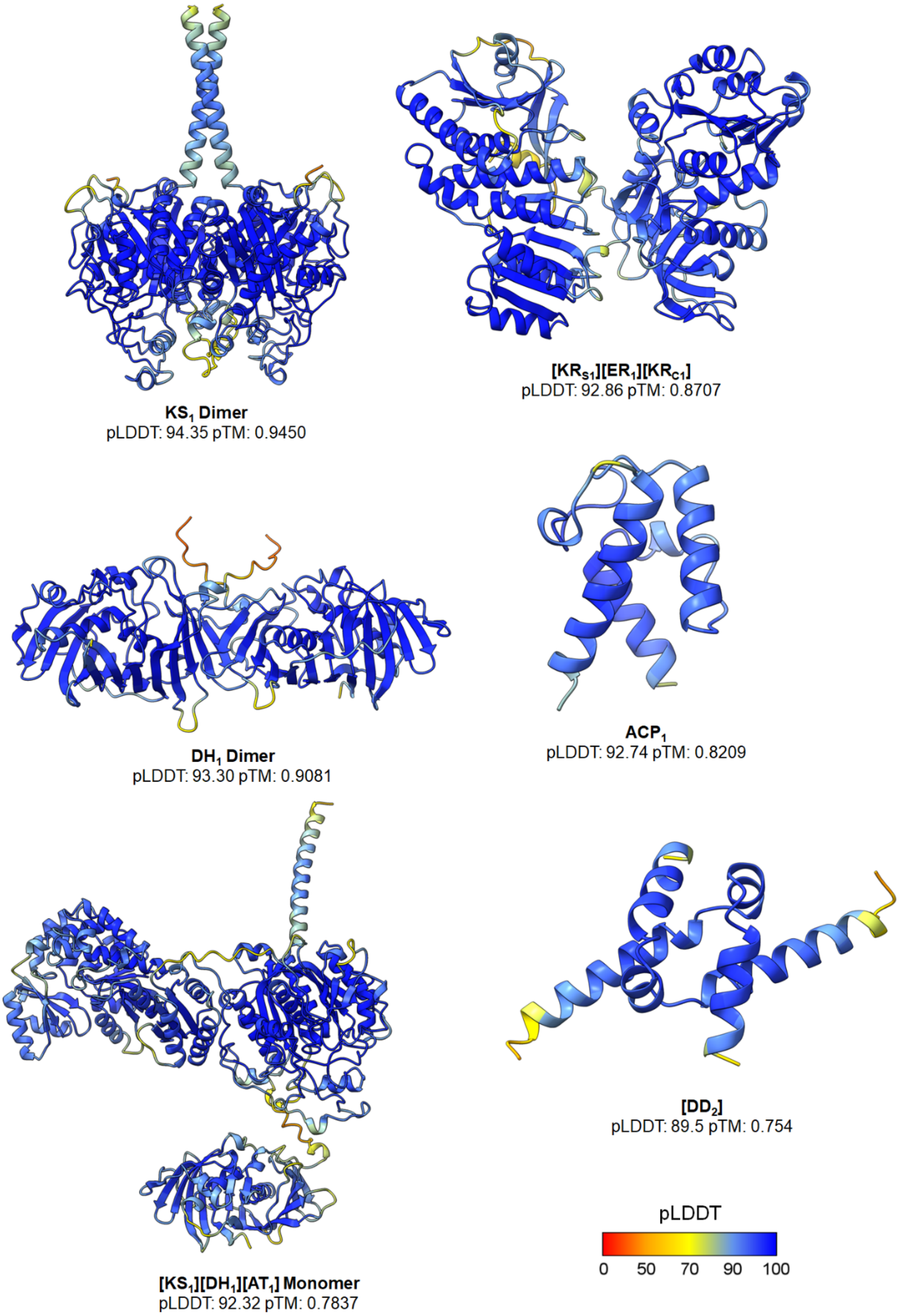

Fig. S5. Confidence metrics for the predicted domain fragments of *Mt*PKS12 [M_1].

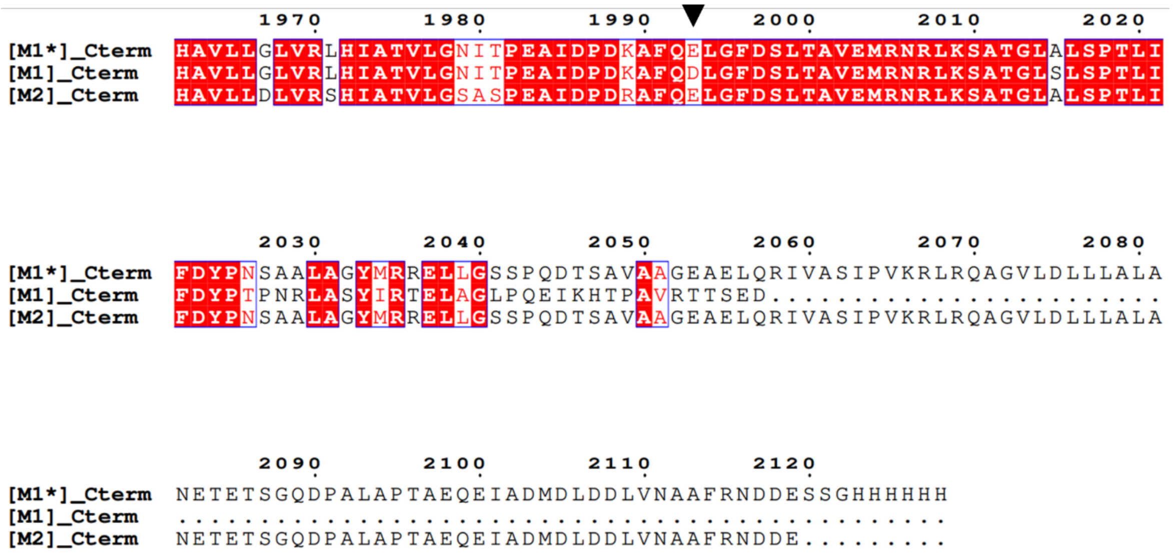

Fig. S6. The C-terminus of [M_1*] aligned with the wildtype *Mt*PKS12 [M_1] and [M_2]. The black arrow indicates where the [M_1*] C-terminus has been modified with [DD_2], derived from [M_2]. An additional C-terminal His-tag was appended to [M_1*] for subsequent IMAC purification. The displayed residue numbering pertains to the [M_1*] primary sequence.

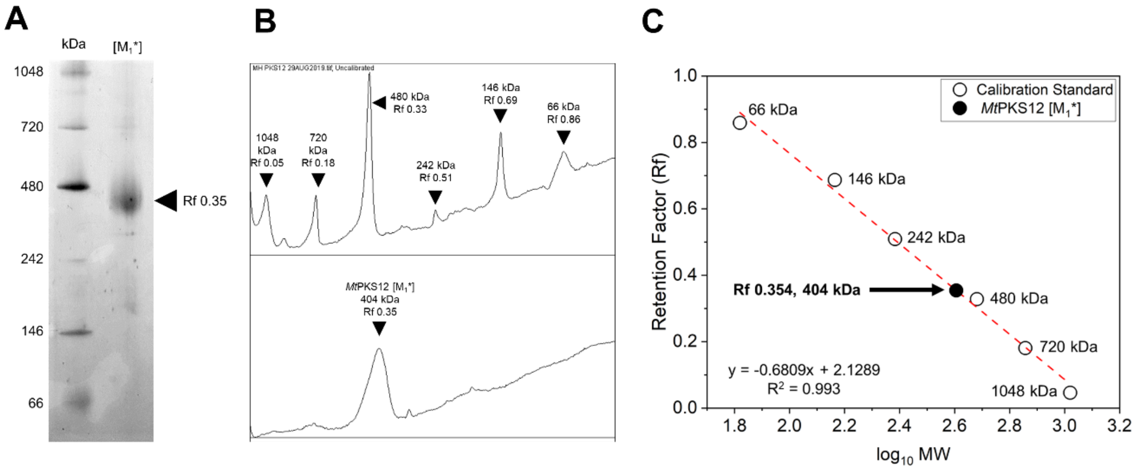

Fig. S7. NativePAGE analysis. A. NativePAGE gel of [M₁*] against NativeMark MW protein standards (see also Table S7). **B.** Densitometry plot of the NativePAGE gel. Rfs and MWs are indicated with black arrows. **C.** NativePAGE calibration showing retention factor (Rf) over the $\log_{10}$ MWs of NativeMark protein standards. The Rf and estimated native MW of [M₁*] is indicated by the black arrow.

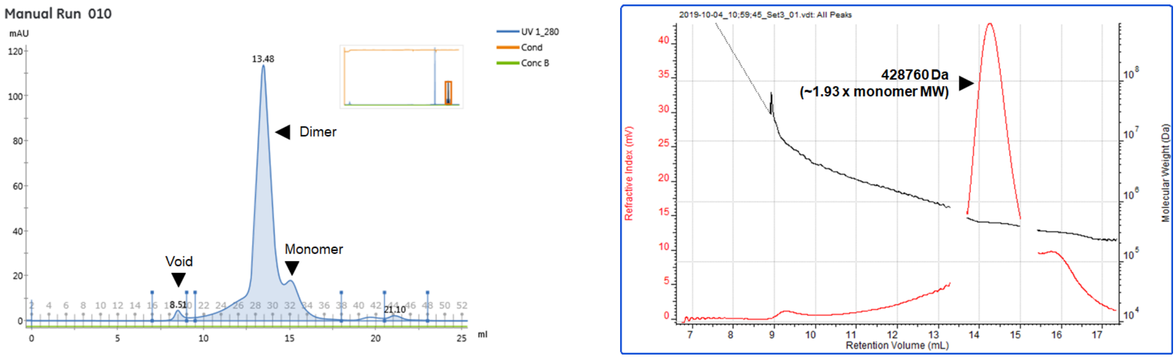

Fig. S8. SEC-MALS analysis of [M₁*]. A Viscotek SEC-MALS 20 was used for estimation of MW. 2 mg mL^{-1} [M₁*] was resolved on a Superose 6 Increase 10/300 GL and detected by tandem UV/vis (280 nm, left panel) and refractive index (RI, right panel). Polydispersity index for dimeric and monomeric species was 1.00 and 1.01, respectively. Buffer D was used as the mobile phase.

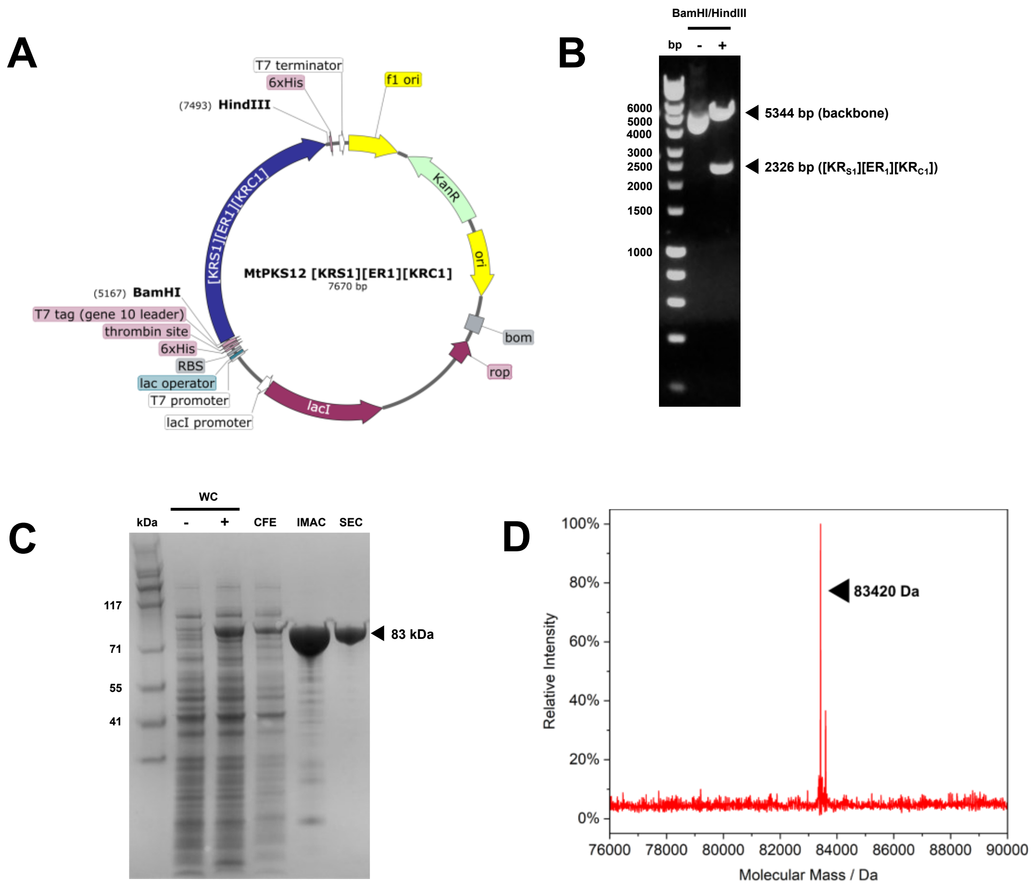

Fig. S9. Cloning and Purification of *Mt*PKS12 [KR_{S1}][ER_1][KR_{C1}]. A. Plasmid map of the pET28a *Mt*PKS12 [KR_{S1}]ER_1][KR_{C1}] expression construct (pIES02). **B.** Restriction endonuclease digest of pIES02. Lanes denoted (-) and (+) show uncut and cut plasmid respectively. A Hyperladder 1 kb DNA standard was used as the ladder. **C.** SDS-PAGE monitoring of protein expression and purification. Lanes denoted WC (*whole cell extract*) show the total protein content of *E. coli* BL21 (DE3) prior to (-) and post-induction (+) using 0.1 mM IPTG. Lane CFE (*cell-free extract*) shows the soluble protein content of *E. coli* BL21 (DE3) post-lysis. **D.** Maximum entropy deconvoluted MS of *Mt*PKS12 [KR_{S1}][ER_1][KR_{C1}].

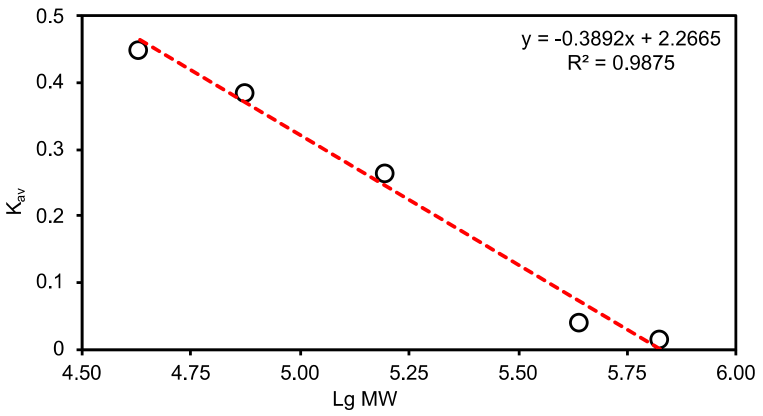

From this calibration plot, the MW of each protein is estimated using the following equations:

$$MW = 10^{\frac{Kav-2.2665}{-0.3892}}$$

$$K_{av} = \frac{V_e - V_o}{V_t - V_o}$$

Where:

V_e = Elution volume

V_o = Void volume (44 mL)

V_t = Total bed volume (120 mL)

Fig. S10. Calibration plot for the HiLoad 16/600 Superdex 200 pg (120 mL) SEC column.

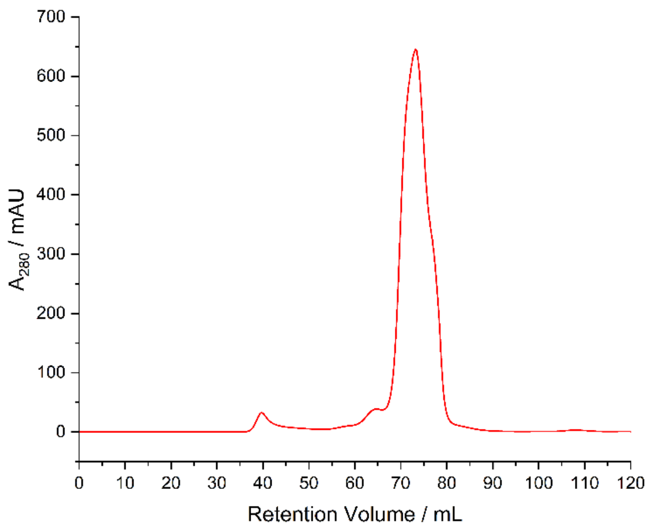

Fig. S11. SEC purification of [KR$_{S1}$][ER$_1$][KR$_{C1}$] using a HiLoad 16/600 Superdex 200 pg (120 mL) SEC column.

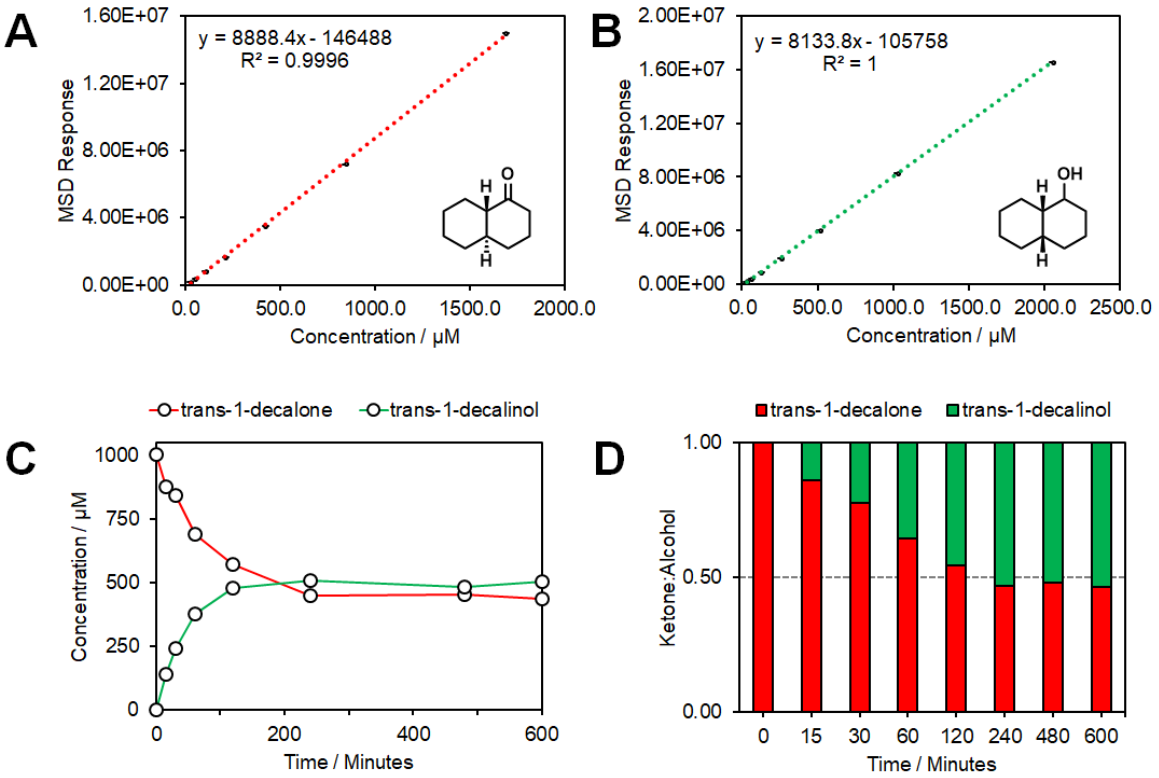

Fig. S12. Supplementary GC/EI-MS data for *trans*-1-decalone assay. A. *trans*-1-decalone (**6**) calibration plot. Each datapoint is an average of three injections (error bars present but obscured). **B.** A *cis*-1-decalinol calibration plot. This isomer was used as a surrogate calibrant in the absence of commercial *trans*-1-decalinol. Each datapoint is an average of three injections (standard deviations present but not visible). **C.** Analyte titres over time. **D.** The ratio of *trans*-1-decalone to *trans*-1-decalinol over time.

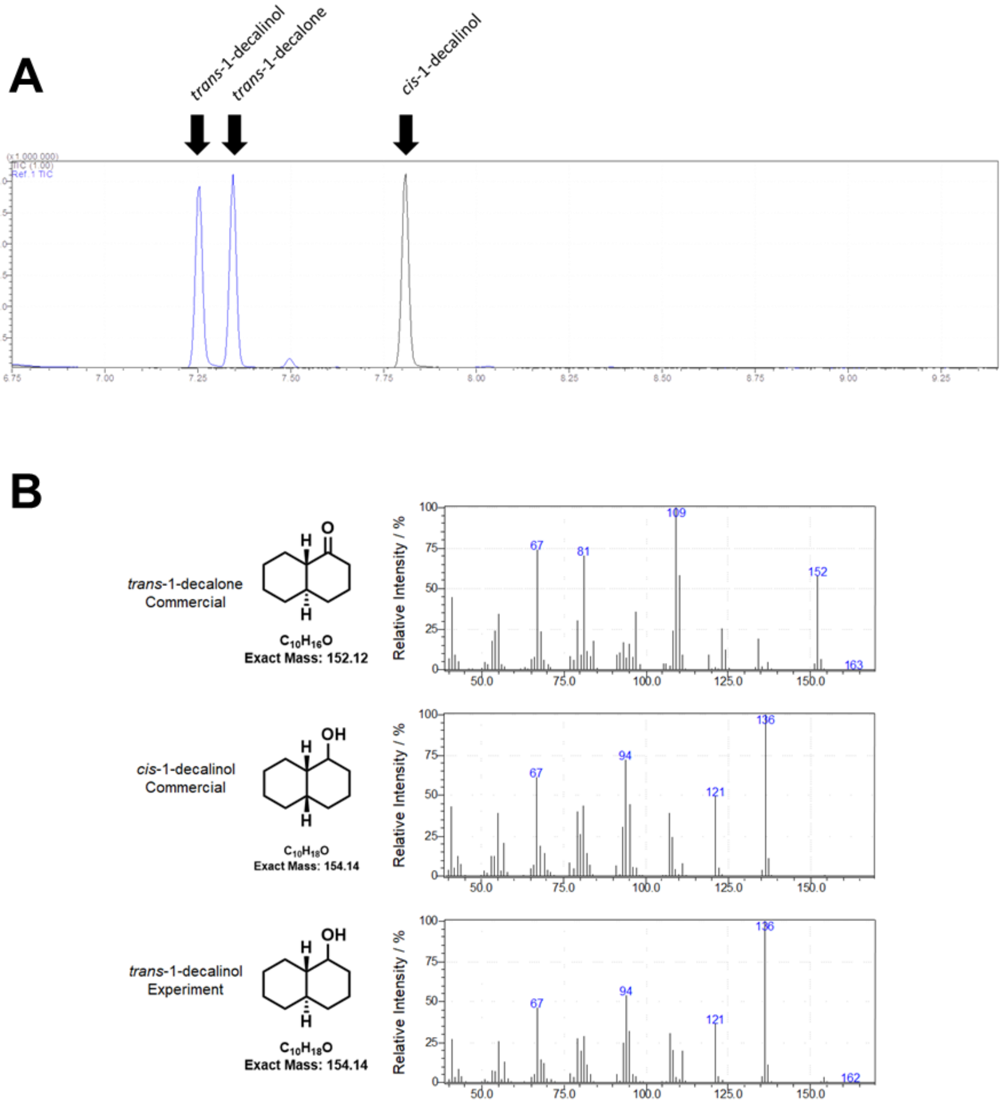

Fig. S13. Raw GC/EI-MS data of *cis/trans*-1-decalone. A. Overlaid, representative raw chromatograms of *trans*-1-decalone + *trans*-1-decalinol (blue) and commercial *cis*-1-decalinol (black). **B.** Raw EI-MS of *trans*-1-decalone (commercial standard, top) and *cis*-1-decalinol (commercial standard, middle). The EI-MS of *trans*-1-decalinol (bottom) was generated by the reduction of *trans*-1-decalone by *Mt*PKS12 [KR_1].

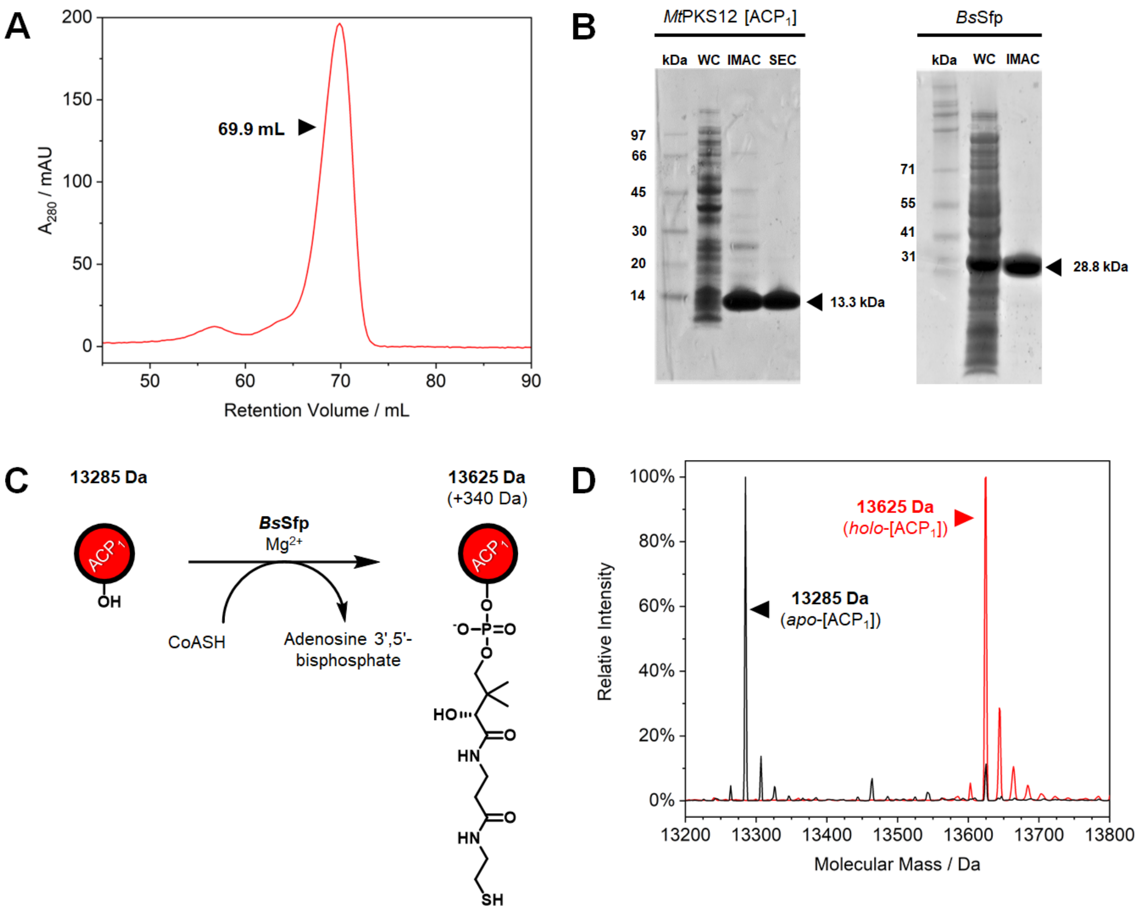

Fig. S14. *Mt*PKS12 [ACP1] handling and post-translational modification by *Bs*Sfp. A. SEC purification of [ACP1] using a HiLoad 16/600 Superdex 75 pg (120 mL) SEC column, monitored by UV-vis (280 nm). **B.** Purification of [ACP1] and *Bs*Sfp from *E. coli*. The Amersham Low MW Calibration Kit and HiMark Pre-stained Protein Standard were used as the ladder for [ACP1] and *Bs*Sfp respectively. Lanes denoted WC (*whole cell extract*) show the total protein content of *E. coli* BL21 (DE3) 18+ hours post-induction using 0.1 mM IPTG. **C.** The anticipated [ACP1] MW increase following modification by *Bs*Sfp. **D.** Deconvoluted protein-MS spectra of *apo*-[ACP1] and *holo*-[ACP1].

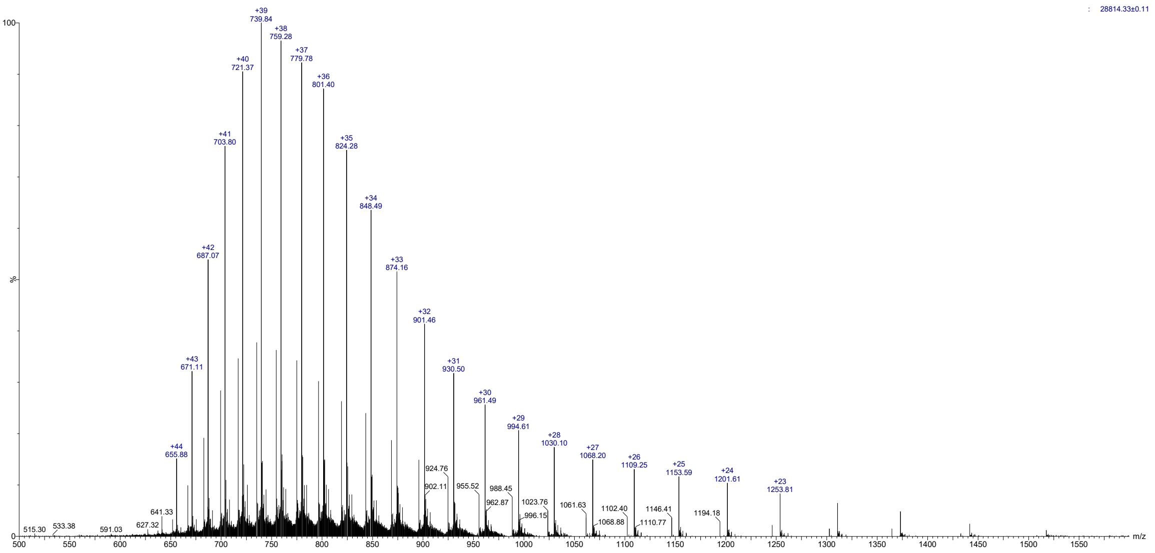

Fig. S15. LC/ESI-MS charge envelope of *Bs*Sfp (theoretical MW = 28765.32 Da). The computed MW (28814.33 ± 0.11 Da) is consistent with a protein with the elemental composition $C_{1283}H_{1943}N_{351}O_{383}S_{11}Mg_2$ (theoretical MW = 28,813.52 Da).

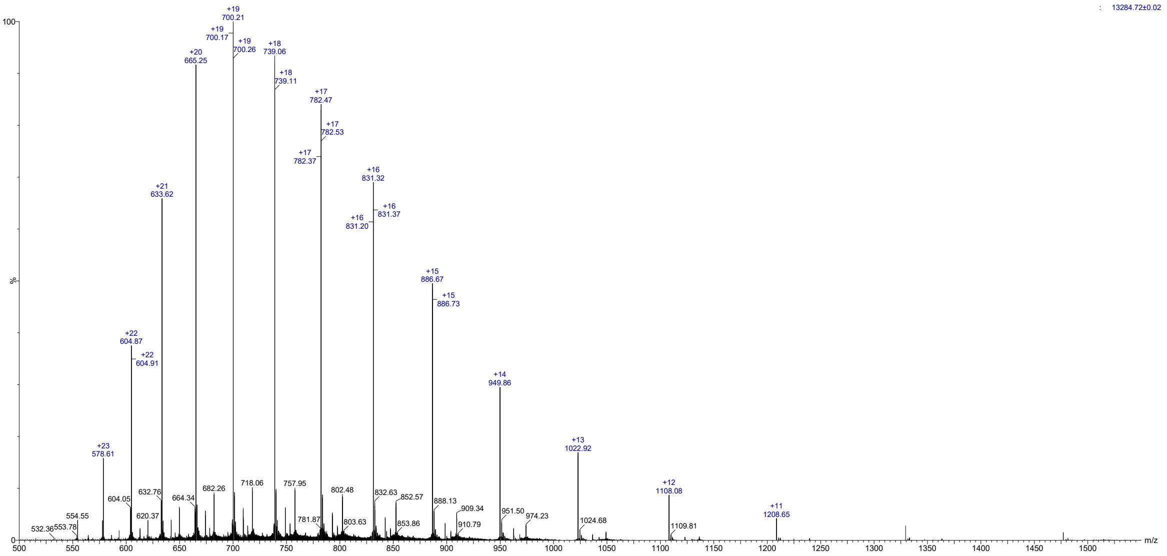

Fig. S16. LC/ESI-MS charge envelope of *Mt*PKS12 *apo*-[ACP1] (theoretical MW = 13260.06 Da). The computed MW (13284.72 ± 0.02 Da) is consistent with a protein with the elemental composition $C_{589}H_{935}N_{171}O_{174}S_2Mg$ (theoretical MW = 13,284.17 Da)

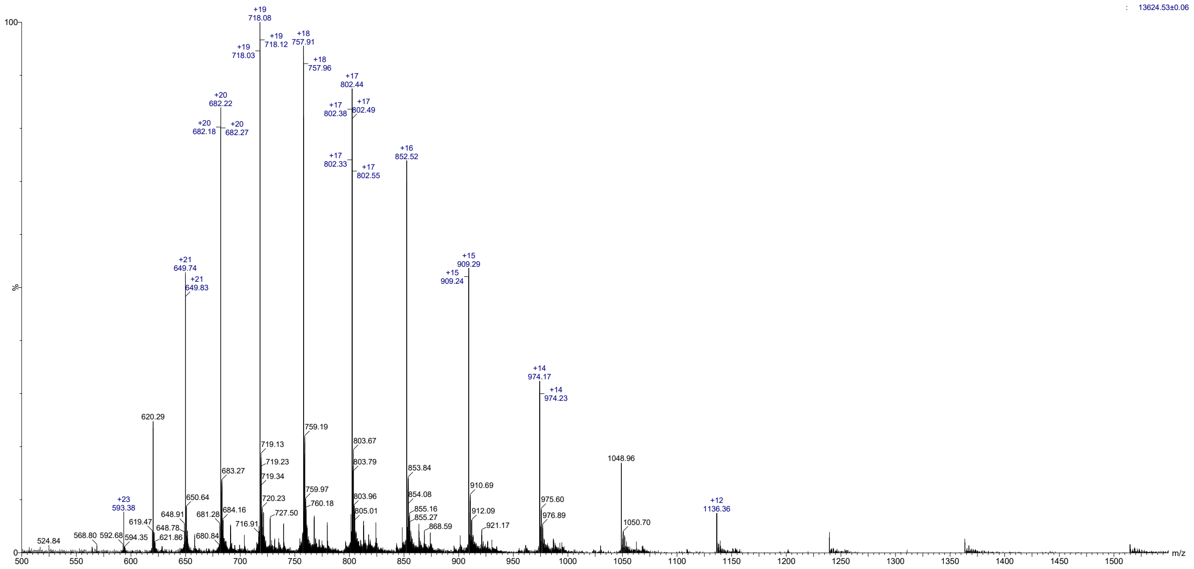

Fig. S17. LC/ESI-MS charge envelope of *Mt*PKS12 *holo*-[ACP1] (theoretical MW = 13,600.39 Da). The computed MW (132624.53 ± 0.06 Da) is consistent with a protein with the elemental composition $C_{600}H_{956}N_{173}O_{180}S_3PMg$ (theoretical MW = 13624.50 Da)

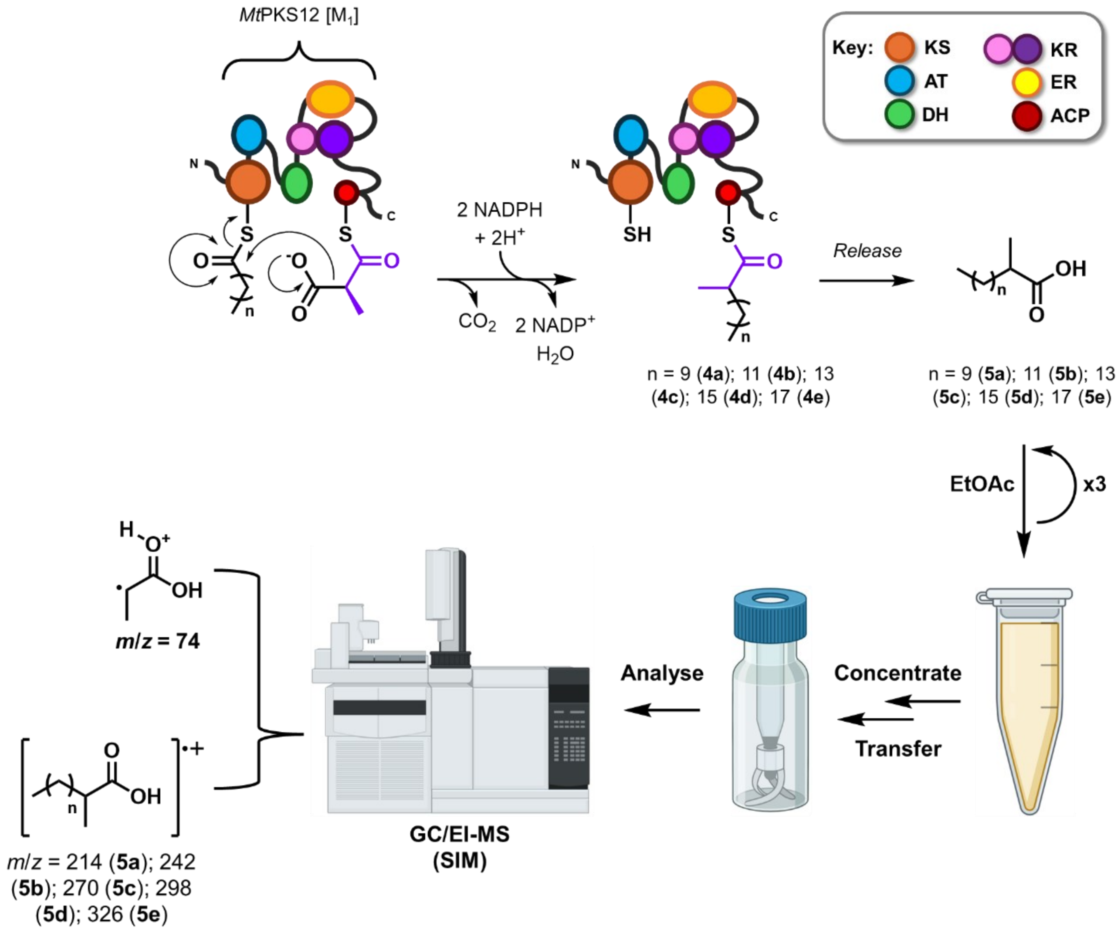

Fig. S18. A graphical summary of the preparation and analysis of [M1*] condensation products. The product is released by alkaline hydrolysis, extracted into organic solvent, dried and resuspended in a minimal volume suitable for GC/EI-MS sampling.

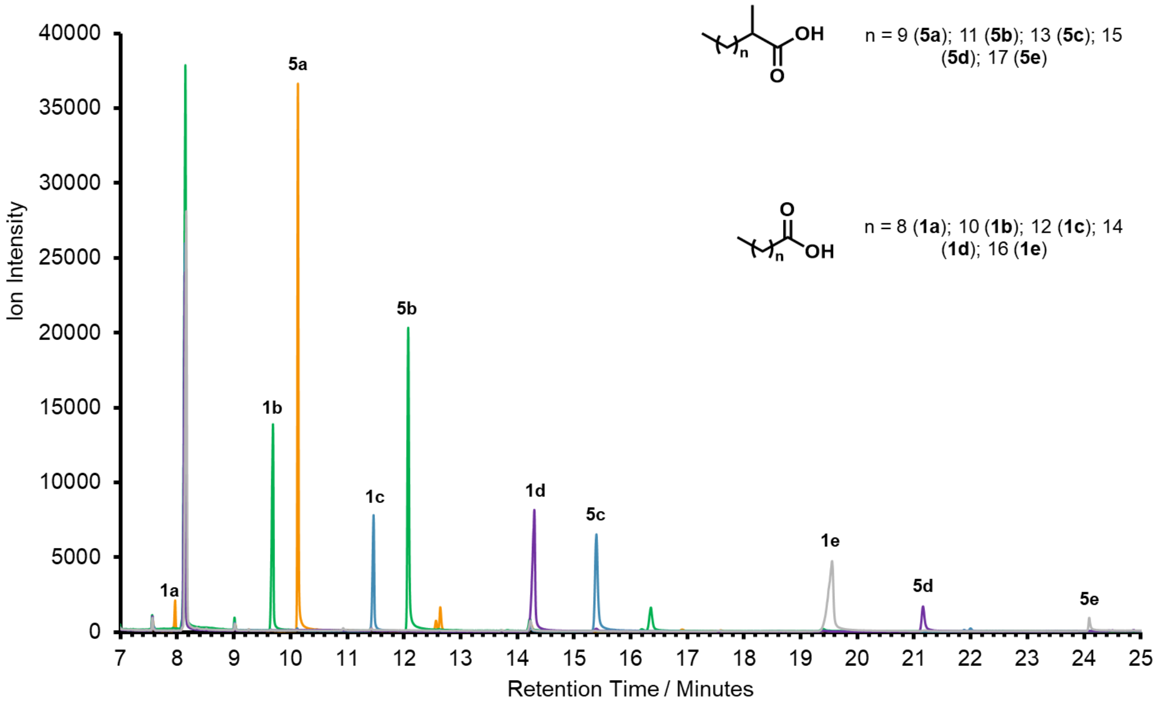

Fig. S19. Overlaid GC-EI/MS chromatograms showing the detection of [M$_1$*] Guerbet-like acids 5a-e and acyl-CoA derived fatty acids 1a-e. The instrument was run in SIM mode (m/z = 74).

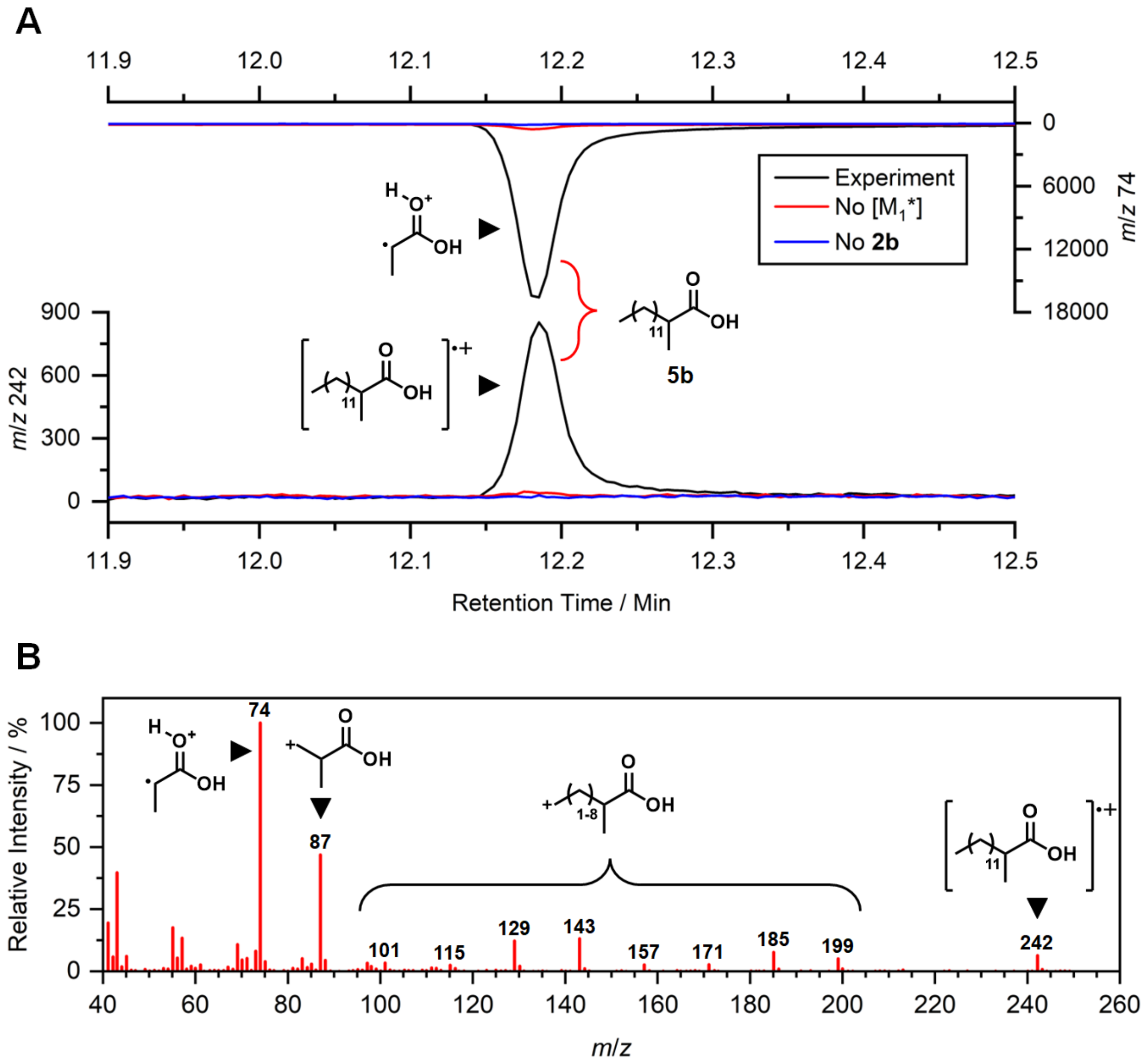

Fig. S20. Detection of [M₁*]-derived 5b (2-methyltetradecanoic acid) by GC/EI-MS. A. SIM detection of the **5b** product (12.19 minutes) by the simultaneous detection of the McLafferty ion (*m*/*z* 74, top chromatogram) and the molecular ion (*m*/*z* 242, bottom chromatogram). Both ions are absent in the negative controls. **B.** Experimentally-determined EI-MS fragmentation pattern of **5b** with key fragment ions assigned.

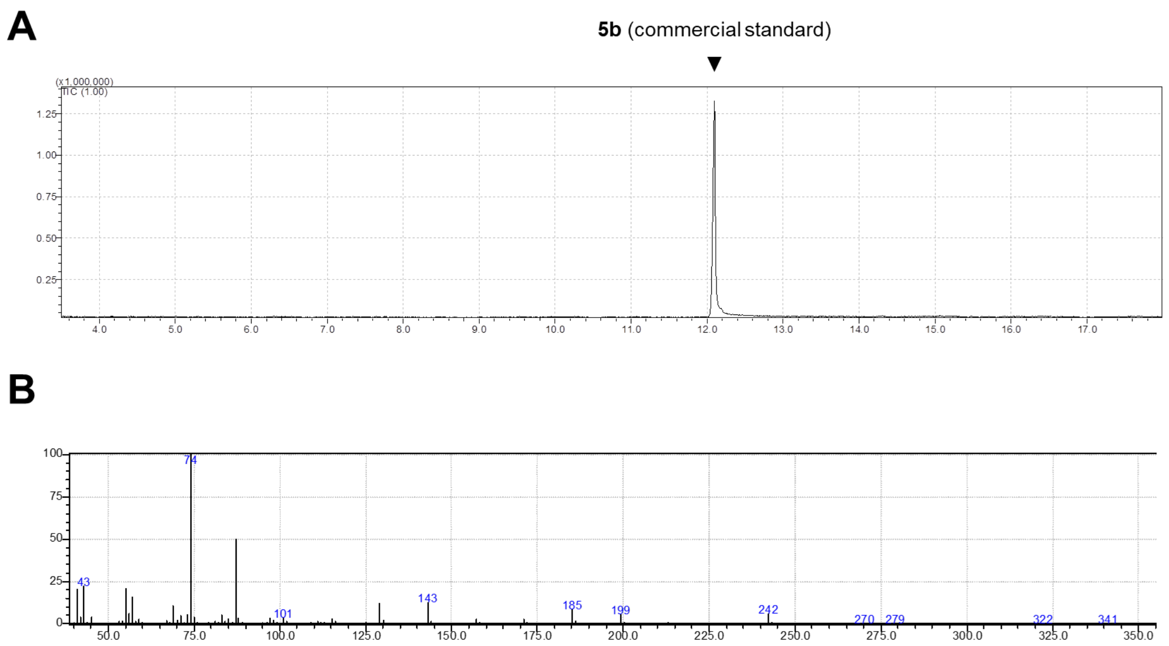

Fig. S21. Commercial standard of 5b (2-methyltetradecanoic acid) by GC/EI-MS. A. Total ion chromatogram (TIC) of a **5b** commercial standard (12.1 minutes). **B.** Raw EI-MS fragmentation pattern of **5b** commercial standard.

RMM	m	n	v	c	Purity	Actual Conc
g / mol	g	mol	L	µM	%	µM
242.403	2.76E-02	1.14E-04	0.050	2277.2	98	2231.7

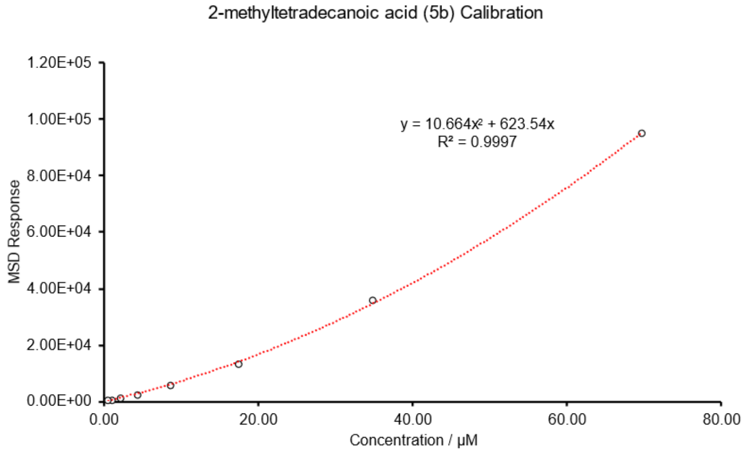

Fig. S22. 5b (2-methyltetradecanoic acid) calibration plot. Each calibration standard was run in triplicate in SIM mode (m/z = 74). The data was fit with a polynomial regression (order 2) to account for excessive curvilinear MSD response at lower concentrations. From the trendline equation y = ax^2 + bx, concentrations can be calculated for any given MSD response using the quadratic formula: $\frac{-b+\sqrt{b^2-4ay}}{2a}$.

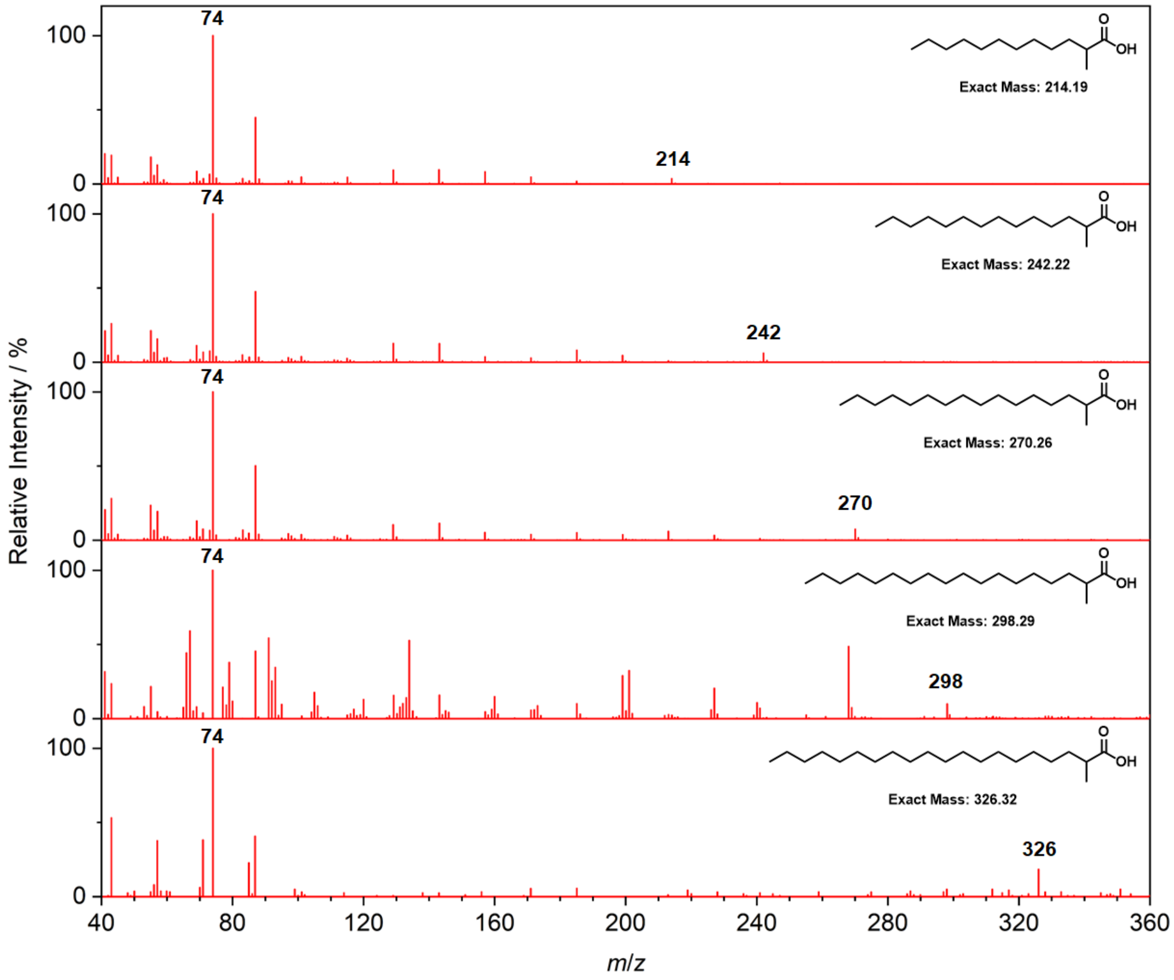

Fig. S23. Experimentally-determined EI-MS of [M$_1$*]-derived products 2-methyldodecanoic acid (**5a**), 2-methyltetradecanoic acid (**5b**), 2-methylhexadecanoic acid (**5c**), 2-methyloctadecanoic acid (**5d**) and 2-methyleicosanoic acid (**5e**). The McLafferty ions and molecular ions are numbered.

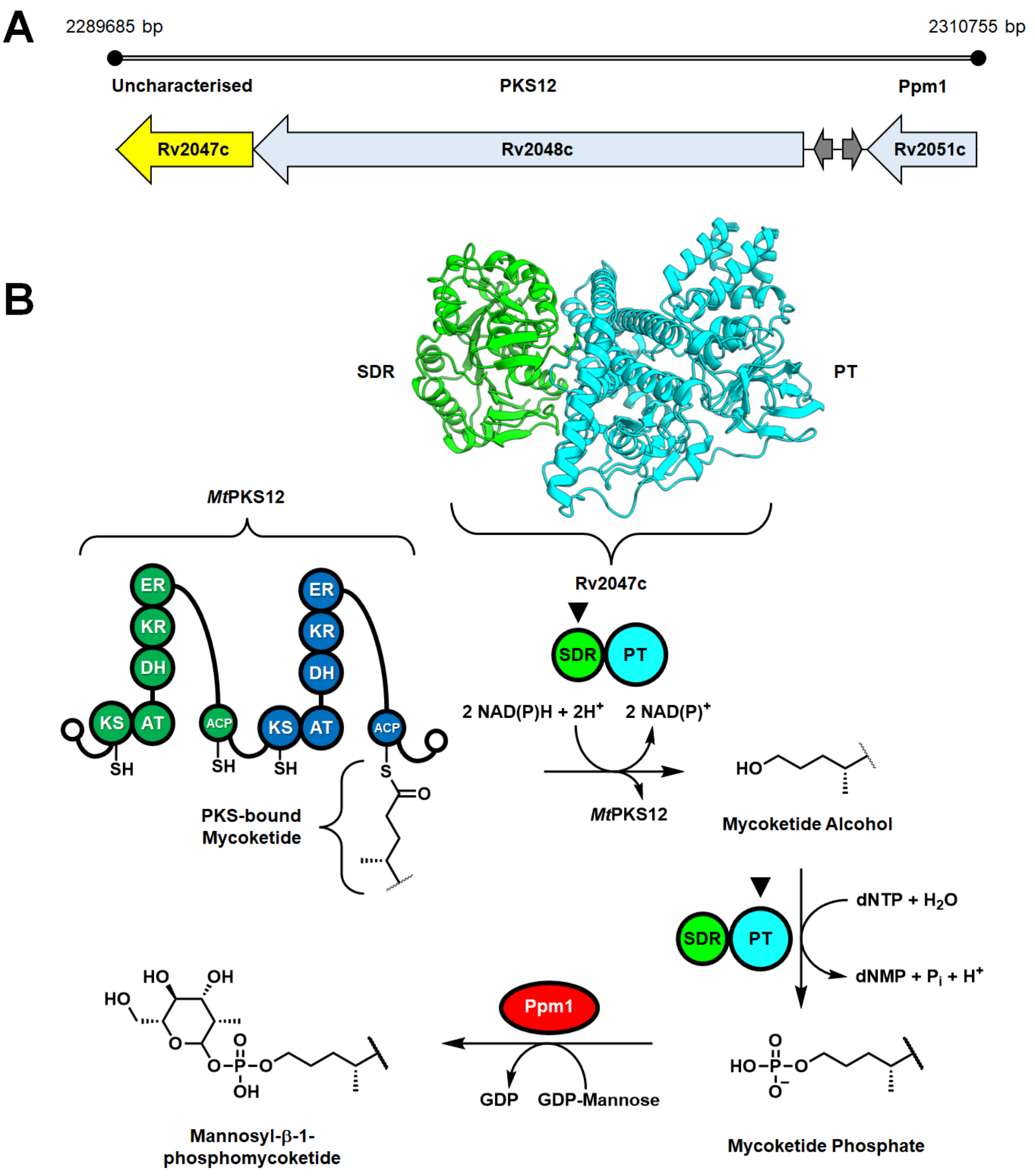

Fig. S24. Putative MPM BGC in *M. tuberculosis* H37Rv. A. The BGC was identified using antiSMASH and features Rv2047c (uncharacterised), Rv2048c (PKS12) and Rv2051c (Ppm1). **B.** The hypothesised biosynthesis of MPM. An AF2 model of Rv2047c is also shown.

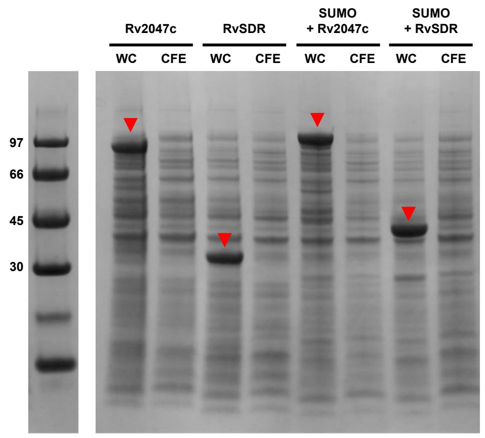

Fig. S25. SDS-PAGE analysis showing the total insolubility of expressed Rv2047c, the SDR domain of Rv2047c (RvSDR), and small ubiquitin-like modifier SUMO fusions thereof. Lanes denoted WC (*whole-cell extract*) represent the total protein content of *E. coli* BL21 (DE3). Lanes denoted CFE (*cell-free extract*) represent the soluble content of lysed *E.coli* BL21 (DE3).

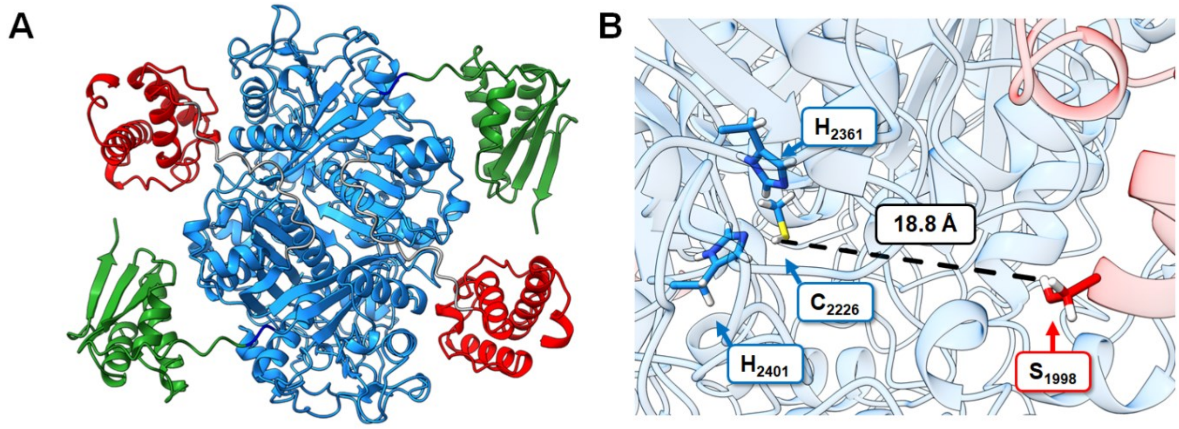

Fig. S26. Modelling the mutual recognition between *Mt*PKS12 [ACP1] and PKS12 [KS2]. A. An AF2 model of homodimeric *Mt*PKS12 [ACP_1][KS_2] (pLDDT = 90.6, pTM = 0.93) with the [KS_2][AT_2] linker (green). [ACP_1] and [KS_2] are coloured red and blue, respectively. B. The relative orientation of the [ACP_1] catalytic serine (S_{1998}) to the [KS_2] Cys-His-His catalytic triad.

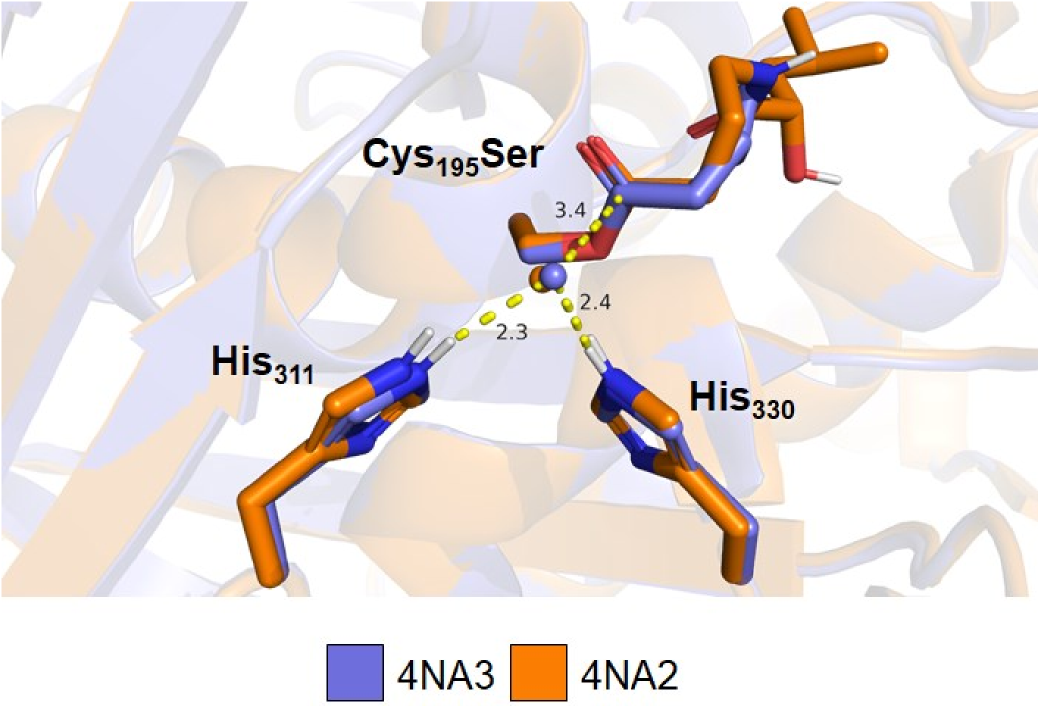

Fig. S27. Superimposition of modified Bacillaene [KS2] catalytic triads (PDB 4NA2 and 4NA3) with substrates bound. Water molecules are shown as coloured spheres. Measurements (in Å) pertain to PDB: 4NA3.

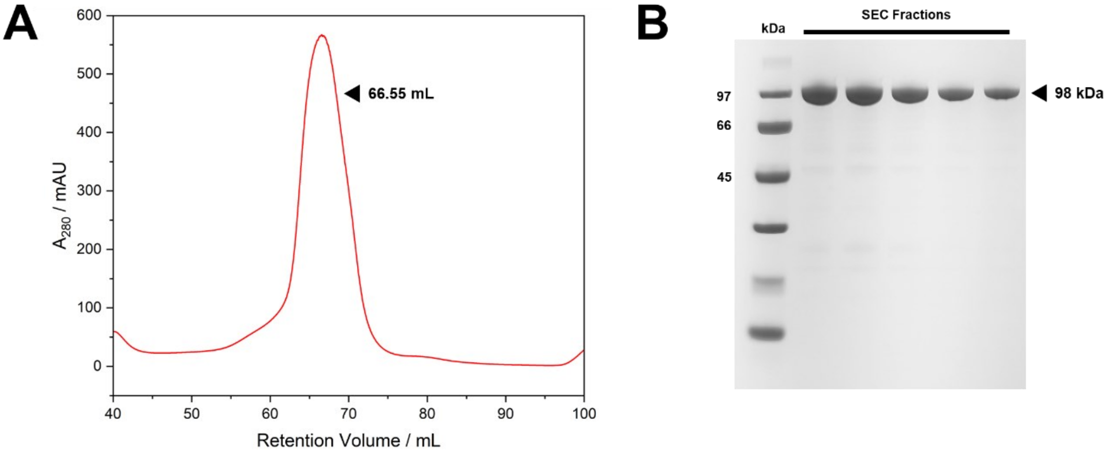

Fig. S28. Purification of *Mt*PKS12 [KS1][AT1]. A. SEC purification of [KS1][AT1] using a HiLoad 16/600 Superdex 200 pg (120 mL) SEC column. **B.** SDS-PAGE monitoring of protein purification by SEC.

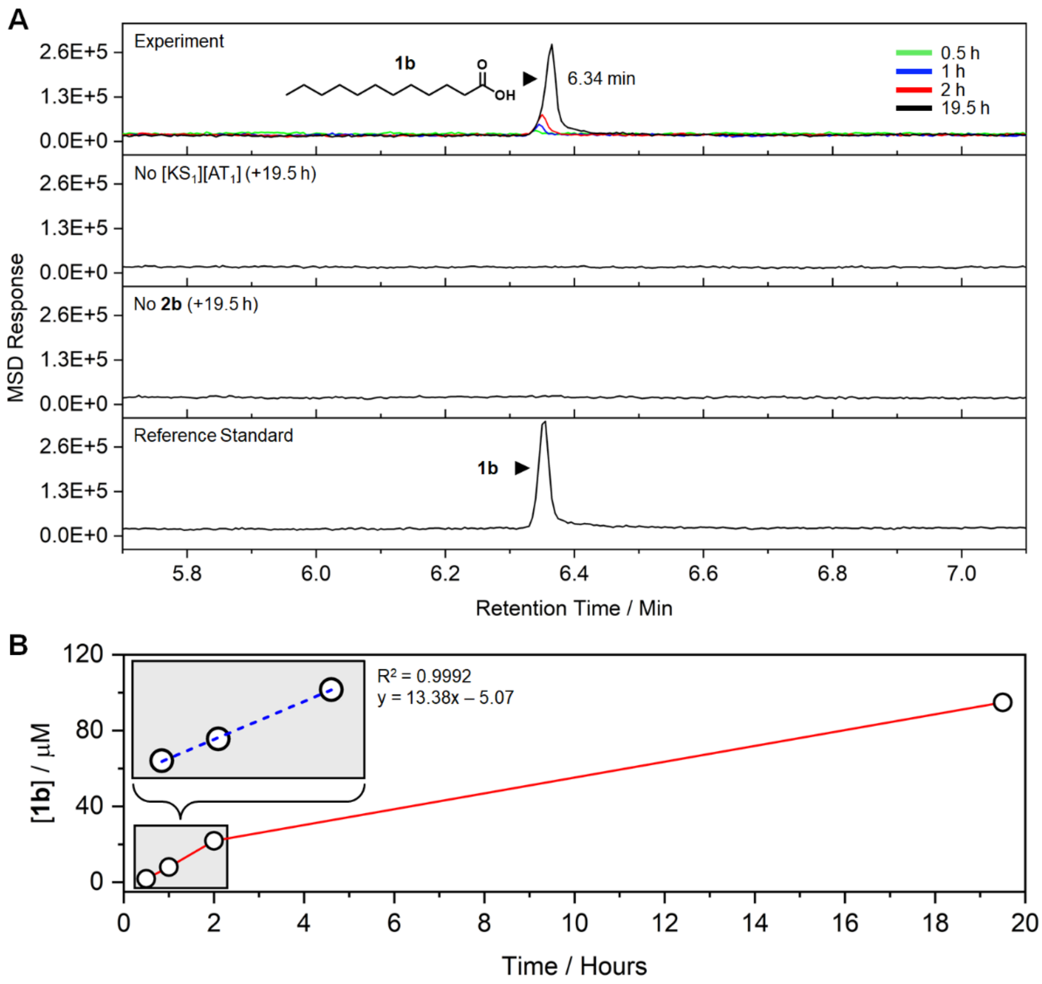

Fig. S29. *Mt*PKS12 [KS_1][AT_1]-catalysed hydrolysis of lauroyl-CoA (2b) to give lauric acid (1a). A. The slow accumulation of **1b** from the [KS_1]-catalysed hydrolysis of **2b**. **1b** is not present in either negative control. **B. 1b** titres over time. The first three timepoints are magnified with a linear fit.

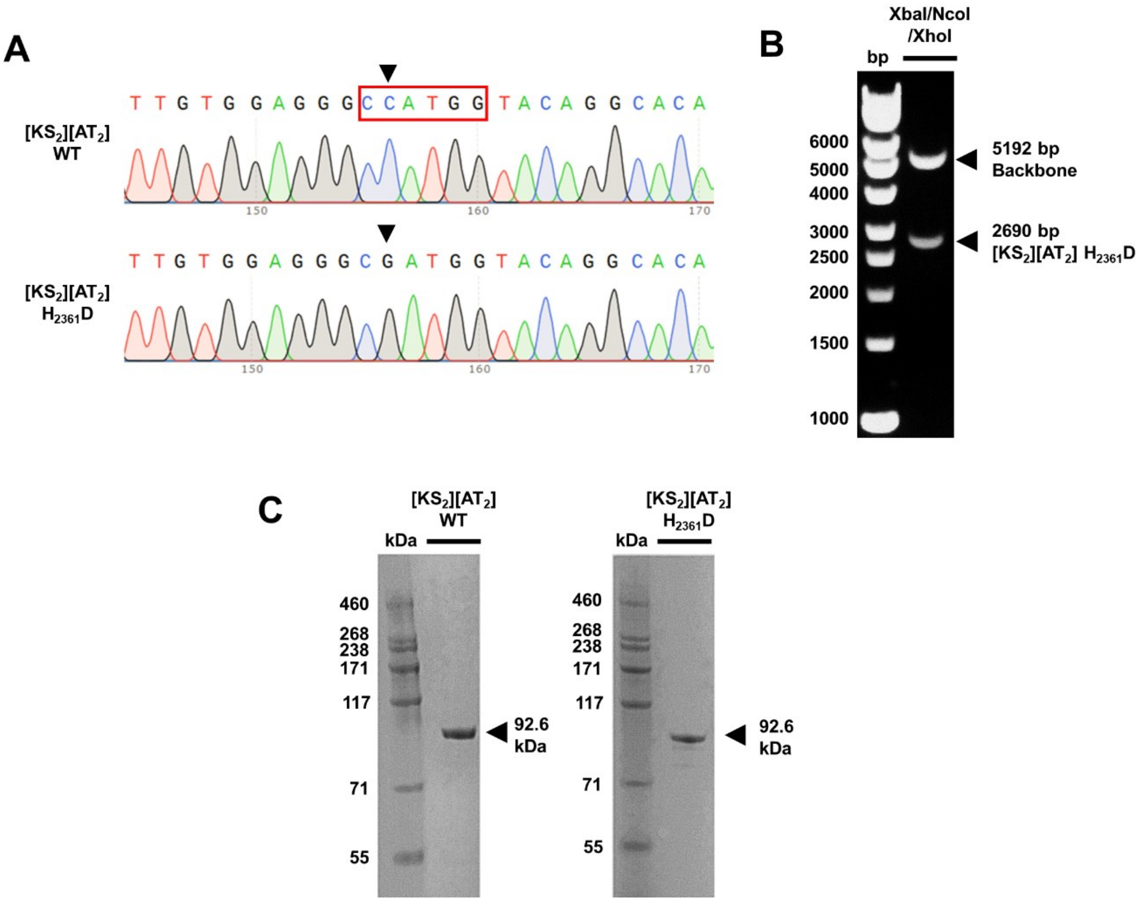

Fig. S30. ***Mt*PKS12 [KS$_2$][AT$_2$] mutagenesis and purification.** **A.** Sequencing data confirming the successful histidine (CAT) to aspartate (GAT) mutation in [KS$_2$] H$_{2361}$D. The natural NcoI restriction site (CCATGG) in the WT sequence is highlighted in a red box. **B.** Restriction enzyme digest of a positive H$_{2361}$D [KS$_2$][AT$_2$] mutant. As this mutation eliminates the NcoI restriction site, only two DNA fragments are generated. **C.** SDS-PAGE of SEC-purified WT and H$_{2361}$D [KS$_2$][AT$_2$]. A HiMark Pre-stained Protein Standard was used as the protein ladder.

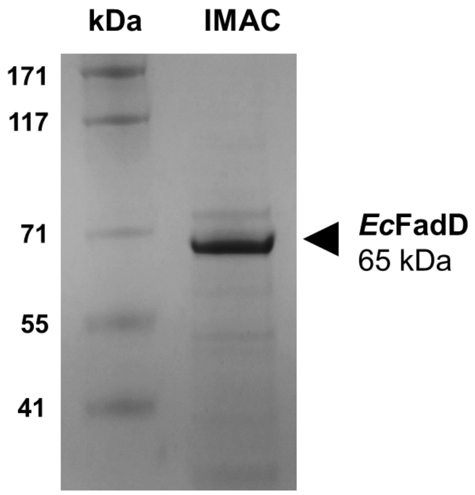

Fig. S31. Expression and purification of *Ec*FadD using HisTrap HP resin. A HiMark Pre-stained Protein Standard was used as the protein ladder.

Plasmid Maps

Sfp-pACYC

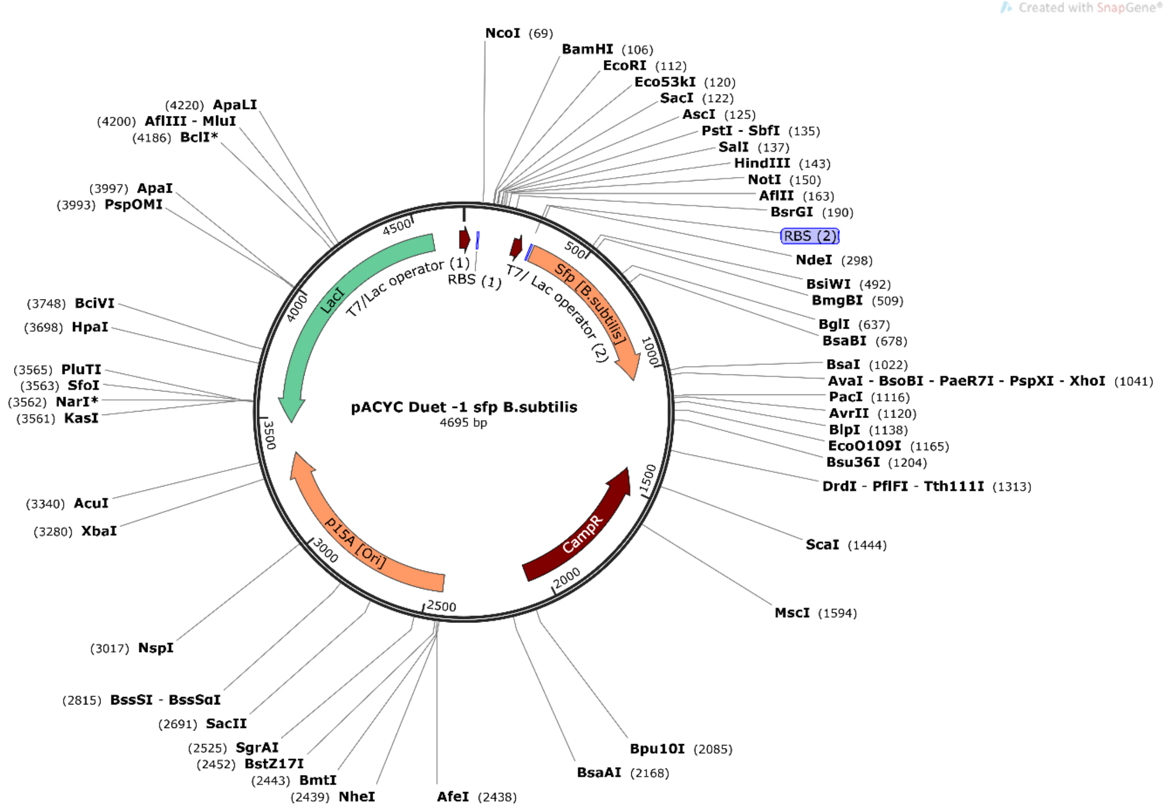

Sequence:

MGHHHHHHHHHHSSGHIDDDDKMKIYGIYMDRPLSQEENERFMTFISPEKREKCRRFYHKEDAHRTLLGDVLVRSVISRQYQLDKSDIRFS TQEYGKPCIPDLPDAHFNISHSGRWVIGAFDSQPIGIDIEKTKPISLEIAKRFFSKTEYSDLLAKDKDEQTDYFYHLWSMKESFIKQEGKGLSLP LDSFSVRLHQDGQVSIELPDSHSPCYIKTYEVDPGYKMAVCAAHPDFPEDITMVSYEELL

pISL52

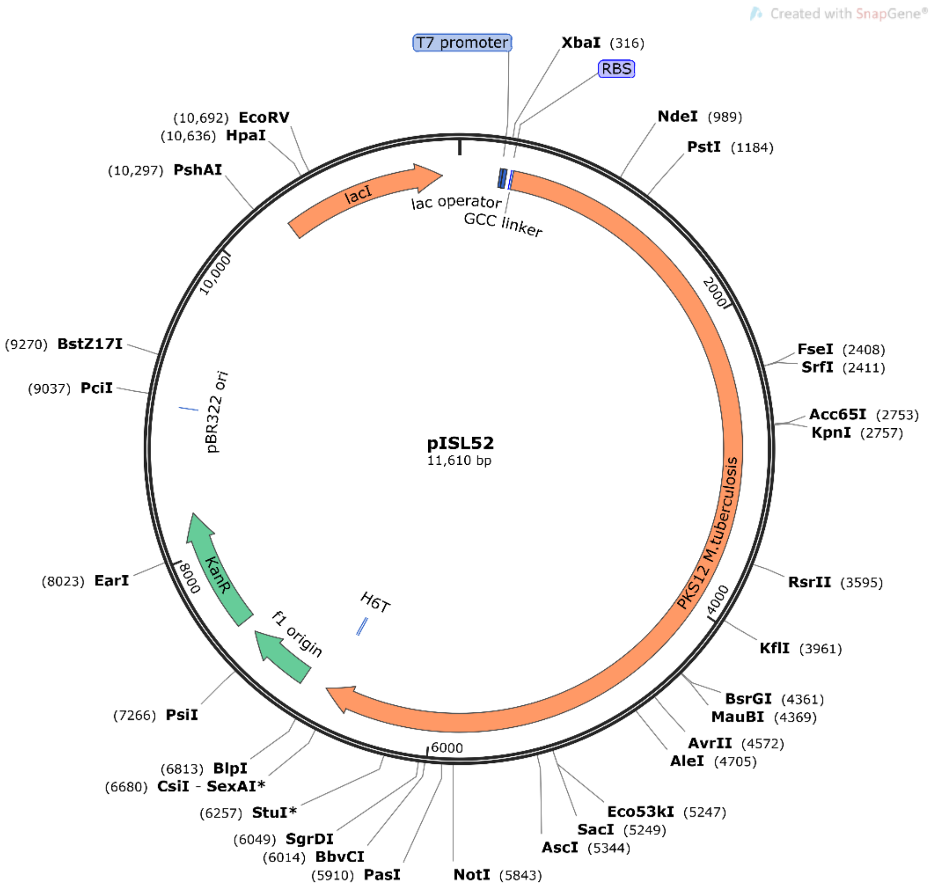

[M_1*] Sequence:

MVDQLQHATEALRKALVQVERLKRTNRALLERSSEPIAIVGMSCRFPGGVDSPEGLWQMVADARDVMSEFPTDRGWDLAGLFDPDPDVR
HKSYARTGGFVDGVADFDPAFFGISPSEALAMDPQHRMLLELSWEALERAGIDPTGLRGSATGVFAGLIVGGYGMLAEEIEGYRLTGMTSS
VASGRVAYVLGLEGPAVSVDTACSSSLVALHMAVGSLRSGECDLALAGGVTVNATPTVFVEFSRHRGLAPDGRCKPYAGRADGVGWSEG
GGMLVLQRLSDARRLGHPVLAVVVGSAVNQDGASNGLTAPNGPSQQRVVRAALANAGLSAAEVDVVEGHGTGTTLGDPIEAQALLATYG
QDRGEPGEPLWLGSVKSNMGHTQAAAGVAGVIKMVLAMRHELLPATLHVDVPSPHVDWSAGAVELLTAPRVWPAGARTRRAGVSSFGIS
GTNAHVIIEAVPVVPRREAGWAGPVVPWVVSAKSESALRGQAARLAAYVRGDDGLDVADVGWSLAGRSVFEHRAVVVGGDRDRLLAGL
DELAGDQLGGSVVRGTATAAGKTVFVFPGQGSQWLGMGIELLDTAPAFAQQIDACAEAFAEFVDWSLVDVLRGAPGAPGLDRVDVVQPV
LFAVMVSLAELWKSVAVHPDAVIGHSQGEIAAAYVAGALSLRDAARVVTLRSKLLAGLAGPGGMVSIACGADQARDLLAPFGDRVSIAVV
NGPSAVVVSGEVGALEELIAVCSTKELRTRRIEVDYASHSVEVEAIRGPLAEALSGIEPRSTRTVFFSTVTGNRLDTAGLDADYWYRNVRQT
VLFDQAVRNACEQGYRTFIESSPHPALITGVEETFAACTDGDSEAIVVPTLGRGDGGLHRFLLSAASAFVAGVAVNWRGTLDGAGYVELPT
YAFDKRRFWLSAEGSGADVSGLGLGASEHPLLGAVVDLPASGGVVLTGRLSPNVQPWLADHAVSDVVLFPGTGFVELAIRAGDEVGCSVL
DELTLAAPLLLPATGSVAVQVVVDAGRDSNSRGVSIFSRADAQAGWLLHAEGILRPGSVEPGADLSVWPPAGAVTVDVADGYERLATRGY
RYGPAFRGLTAMWARGEEIFAEVRLPEAAGGVGGFGVHPALLDAVLHAVVIAGDPDELALPFAWQGVSLHATGASAVRARIAPAGPSAVS
VELADGLGLPVLSVASMVARPVTERQLLAAVSGSGPDRLFEVIWSPASAATSPGPTPAYQIFESVAADQDPVAGSYVRSHQALAAVQSWLT
DHESGVLVVATRGAMALPREDVADLAGAAVWGLVRSAQTEHPGRIVLVDSDAATDDAAIAMALATGEPQVVLRGGQVYTARVRGSRAA
DAILVPPGDGPWRLGLGSAGTFENLRLEPVPNADAPLGPGQVRVAMRAIAANFRDIMITLGMFTHDALLGGEGAGVVVEVGPGVTEFSVG
DSVFGFFPDGSGTLVAGDVRLLLPMPADWSYAEAAAISAVFTTAYYAFIHLADVQPGQRVLIHAGTGGVGMAAVQLARHLGLEVFATASK
GKWDTLRAMGFDDDHISDSRSLEFEDKFRAATGGRGFDVVLDSLAGEFVDASLRLVAPGGVFLEMGKTDIRDPGVIAQQYPGVRYRAFDL
FEPGRPRMHQYMLELATLFGDGVLRPLPVTTFDVRRAPAALRYLSQARHTGKVVMLMPGSWAAGTVLITGGTGMAGSAVARHVVARHG
VRNLVLVSRRGPDAPGAAELVAELAAAGAQVQVVACDAADRAALAKVIADIPVQHPLSGVIHTAGALDDAVVMSLTPDRVDVVLRSKVD
AAWHLHELTRDLDVSAFVMFSSMAGLVGSSGQANYAAANSFLDALAAHRRAHGLPAISLGWGLWDQASAMTGGLDAADLARLGREGVL
ALSTAEALELFDTAMIVDEPFLAPARIDLTALRAHAVAVPPMFSDLASAPTRRQVDDSVAAAKSKSALAHRLHGLPEAEQHAVLLGLVRLH
IATVLGNITPEAIDPDKAFQELGFDSLTAVEMRNRLKSATGLALSPTLIFDYPNSAALAGYMRRELLGSSPQDTSAVAAGEAELQRIVASIPV
KRLRQAGVLDLLLALANETETSGQDPALAPTAEQEIADMDLDDLVNAAFRNDDESSGHHHHHH*

pIES01

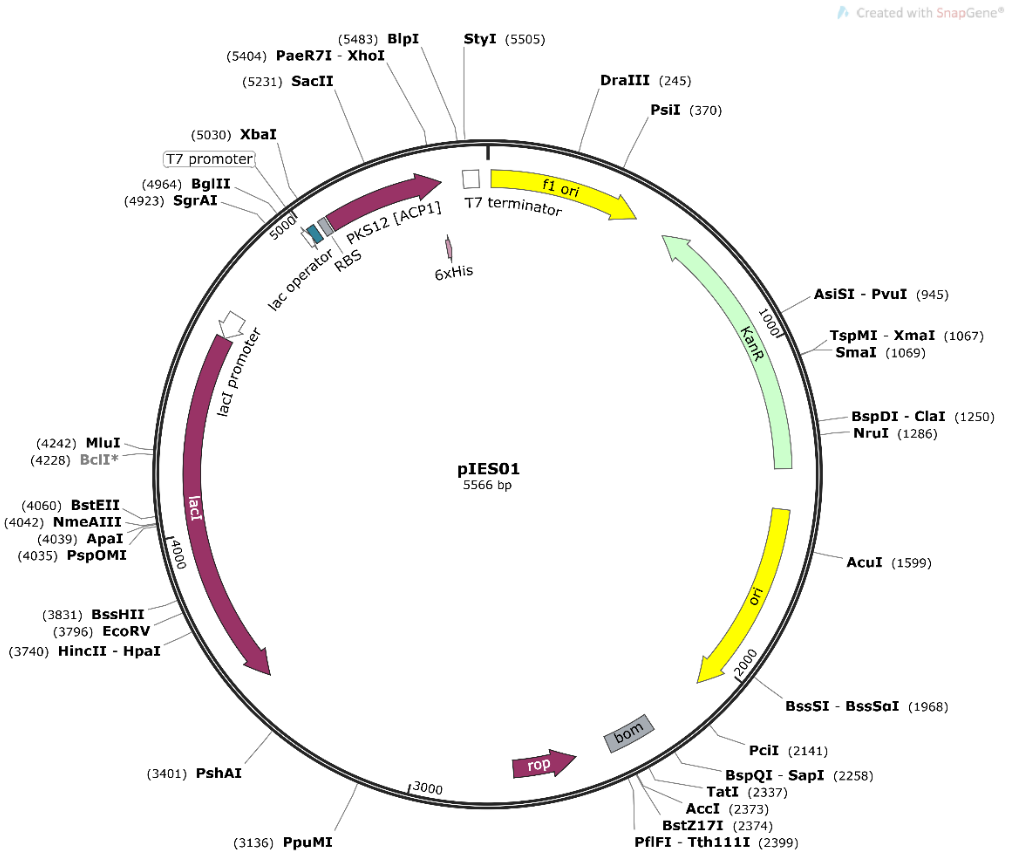

*Mt*PKS12 [ACP$_1$] Sequence:

MLAHRLHGLPEAEQHAVLLGLVRLHIATVLGNITPEAIDPDKAFQDLGFDSLTAVEMRNRLKSATGLSLSPTLIFDYPTPNRLASYIRTELAG
LPQEIKHTPAVRTTSEEDLEHHHHHH*

pIES02

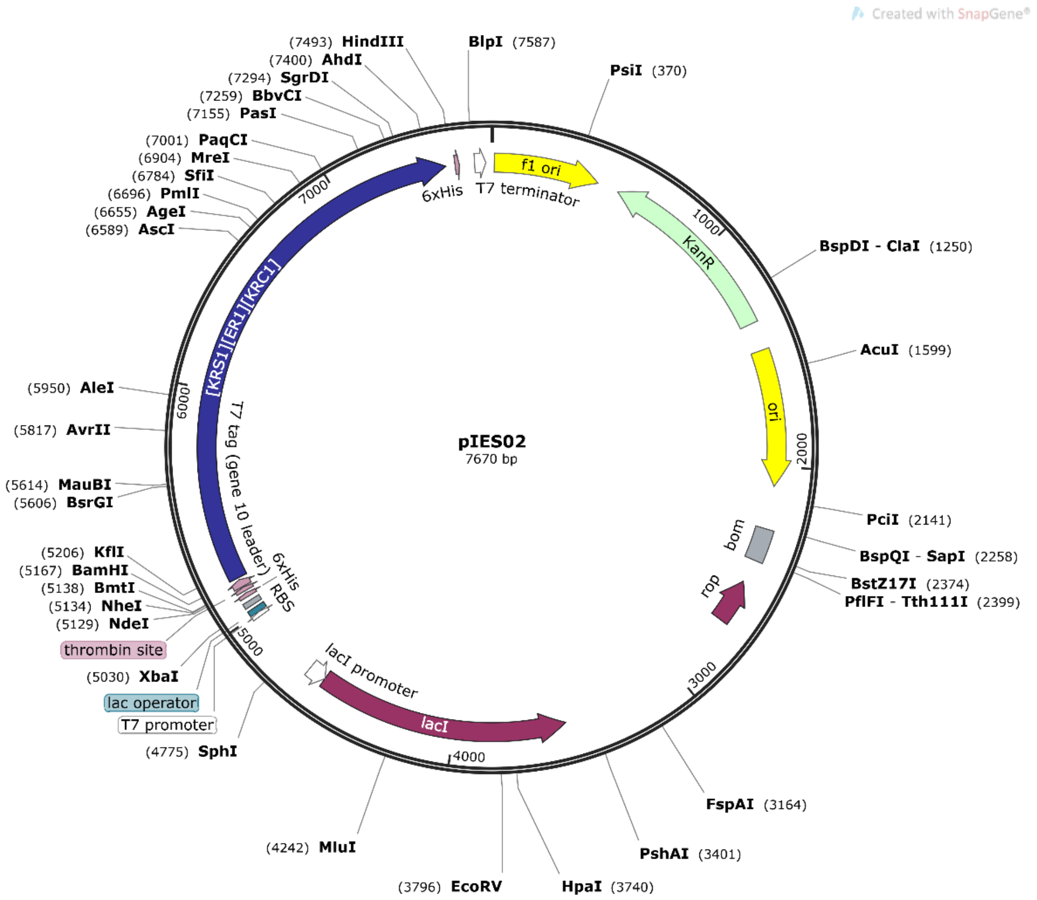

*Mt*PKS12 [KR_{s1}][ER_1][KR_{C1}] Sequence:

MGSSHHHHHHSSGLVPRGSHMASMTGGQQMGRGSERQLLAAVSGSGPDRLFEVIWSPASAATSPGPTPAYQIFESVAADQDPVAGSYVRS
HQALAAVQSWLTDHESGVLVVATRGAMALPREDVADLAGAAVWGLVRSAQTEHPGRIVLVDSDAATDDAAIAMALATGEPQVVLRGGQ
VYTARVRGSRAADAILVPPGDGPWRLGLGSAGTFENLRLEPVPNADAPLGPGQVRVAMRAIAANFRDIMITLGMFTHDALLGGEGAGVVV
EVGPGVTEFSVGDSVFGFFPDGSGTLVAGDVRLLLPMPADWSYAEAAAISAVFTTAYYAFIHLADVQPGQRVLIHAGTGGVGMAAVQLAR
HLGLEVFATASKGKWDTLRAMGFDDDHISDSRSLEFEDKFRAATGGRGFDVVLDSLAGEFVDASLRLVAPGGVFLEMGKTDIRDPGVIAQ
QYPGVRYRAFDLFEPGRPRMHQYMLELATLFGDGVLRPLPVTTFDVRRAPAALRYLSQARHTGKVVMLMPGSWAAGTVLITGGTGMAGS
AVARHVVARHGVRNLVLVSRRGPDAPGAAELVAELAAAGAQVQVVACDAADRAALAKVIADIPVQHPLSGVIHTAGALDDAVVMSLTP
DRVDVVLRSKVDAAWHLHELTRDLDVSAFVMFSSMAGLVGSSGQANYAAANSFLDALAAHRRAHGLPAISLGWGLWDQASAMTGGLD
AADLARLGREGVLALSTAEALELFDTAMIVDEPFLAPARIDLTALRAHAVAVPPMFSDLASAPTRRQVDDSVAAAKSKSALAHRLHGL*

pIES03

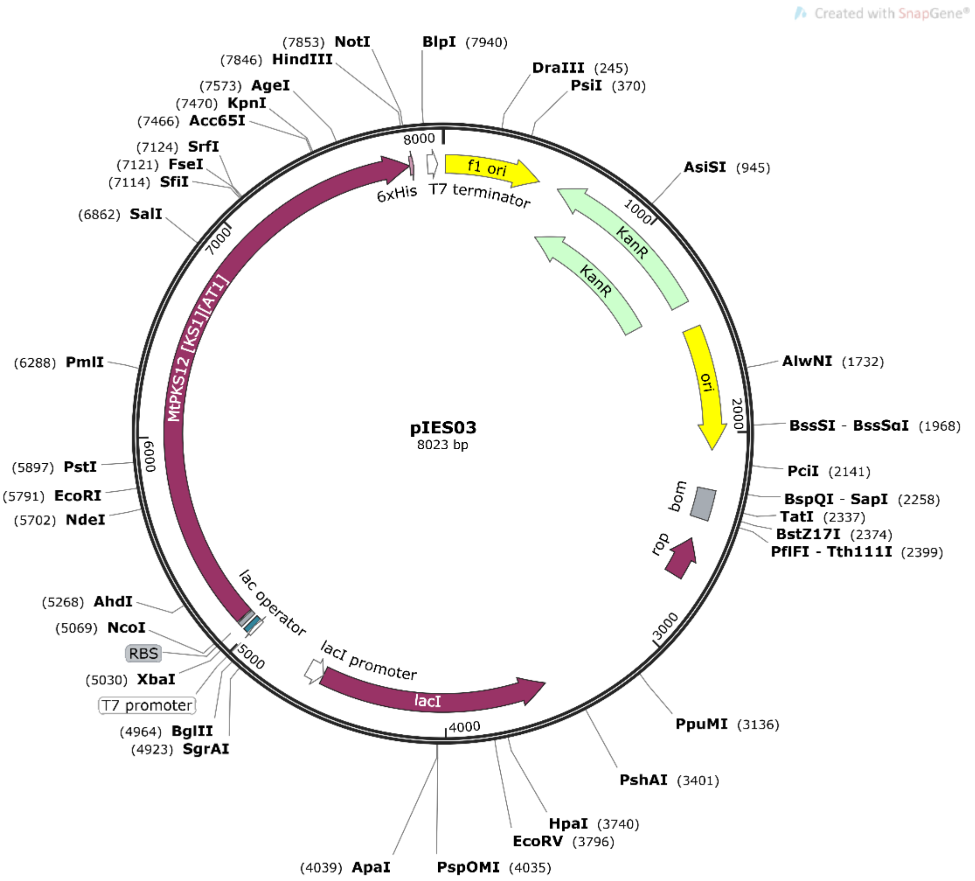

*Mt*PKS12 [KS$_1$][AT$_1$] Sequence:

MVDQLQHATEALRKALVQVERLKRTNRALLERSSEPIAIVGMSCRFPGGVDSPEGLWQMVADARDVMSEFPTDRGWDLAGLFDPDPDVR
HKSYARTGGFVDGVADFDPAFFGISPSEALAMDPQHRMLLELSWEALERAGIDPTGLRGSATGVFAGLIVGGYGMLAEEIEGYRLTGMTSS
VASGRVAYVLGLEGPAVSVDTACSSSLVALHMAVGSLRSGECDLALAGGVTVNATPTVFVEFSRHRGLAPDGRCKPYAGRADGVGWSEG
GGMLVLQRLSDARRLGHPVLAVVVGSAVNQDGASNGLTAPNGPSQQRVVRAALANAGLSAAEVDVVEGHGTGTTLGDPIEAQALLATYG
QDRGEPGEPLWLGSVKSNMGHTQAAAGVAGVIKMVLAMRHELLPATLHVDVPSPHVDWSAGAVELLTAPRVWPAGARTRRAGVSSFGIS
GTNAHVIIEAVPVVPRREAGWAGPVVPWVVSAKSESALRGQAARLAAYVRGDDGLDVADVGWSLAGRSVFEHRAVVVGGDRDRLLAGL
DELAGDQLGGSVVRGTATAAGKTVFVFPGQGSQWLGMGIELLDTAPAFAQQIDACAEAFAEFVDWSLVDVLRGAPGAPGLDRVDVVQPV
LFAVMVSLAELWKSVAVHPDAVIGHSQGEIAAAYVAGALSLRDAARVVTLRSKLLAGLAGPGGMVSIACGADQARDLLAPFGDRVSIAVV
NGPSAVVVSGEVGALEELIAVCSTKELRTRRIEVDYASHSVEVEAIRGPLAEALSGIEPRSTRTVFFSTVTGNRLDTAGLDADYWYRNVRQT
VLFDQAVRNACEQGYRTFIESSPHPALITGVEETFAACTDGDSEAIVVPTLGRGDGGLHRFLLSAASAFVAGVAVNWRGTLDGAGYVELPT
YAFDKRRFWLSAEGSGADVSGLGLGASGKLAAALEHHHHHH*

pIES11

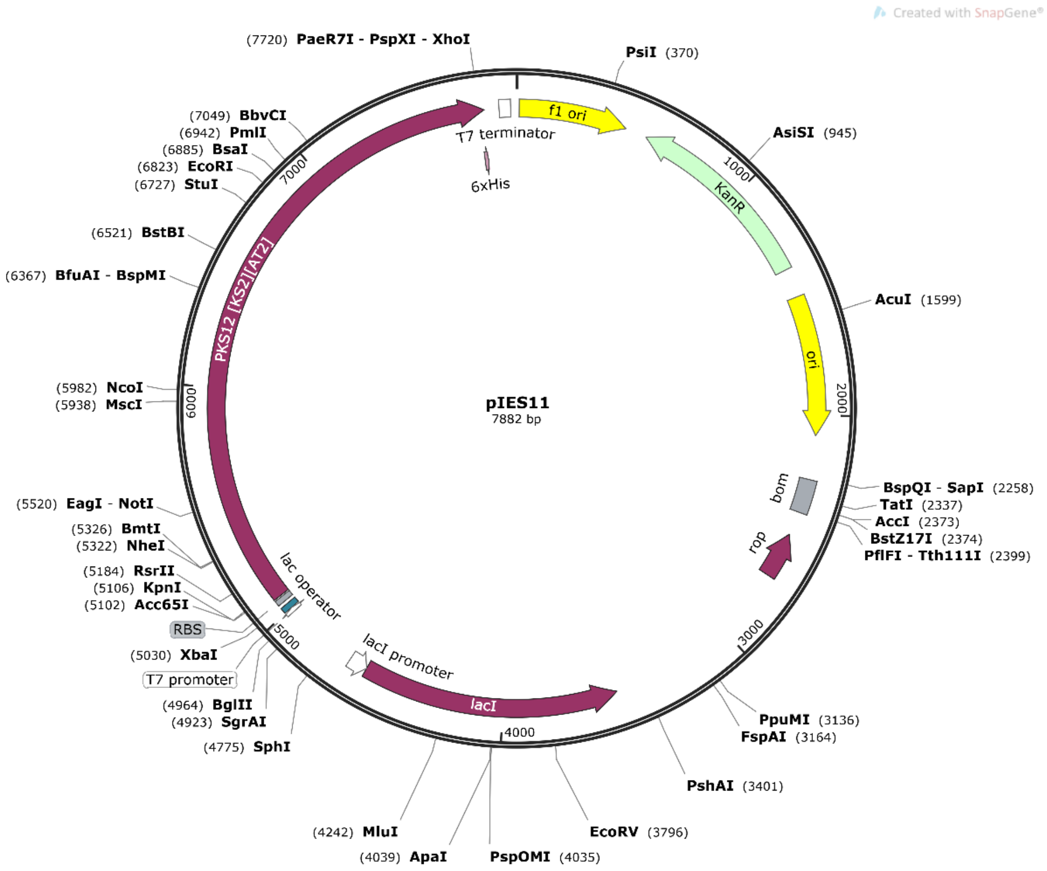

*Mt*PKS12 [KS_2][AT_2] (wildtype) Sequence:

MPIAIVGMACRYPGGVNSPDDMWDMLIQGRDVLSEFPADRGWDLAGLYNPDPDAAGACYTRTGGFVDGVGDFDPAFFGVGPSEALAMD
PQHRMLLELSWEALERAGIDPTGLRGSATGVFAGVMTQGYGMFAAEPVEGFRLTGQLSSVASGRVAYVLGLEGPAVSVDTACSSSLVALH
MAVGSLRSGECDLALAGGVTVNATPDIFVEFSRWRGLSPDGRCKAFAAAADGTGFSEGGGMLVLQRLSDARRLGHPVLAVVVGSAVNQD
GASNGLTAPNGPSQQRVVRAALANAGLSAAEVDVVEG**H**GTGTTLGDPIEAQALLATYGQDRGEPGEPLWLGSVKSNMGHTQAAAGVAG
VIKMVLAMRHELLPATLHVDVPSPHVDWSAGAVELLTAPRVWPAGARTRRAGVSSFGISGTNAHVIIEAVPVVPRREAGWAGPVVPWVVS
AKSESALRGQAARLAAYVRGDDGLDVADVGWSLAGRSVFEHRAVVVGGDRDRLLAGLDELAGDQLGGSVVRGTATAAGKTVFVFPGQG
SQWLGMGMGLHAGYPVFAEAFNTVVGELDRHLLRPLREVMWGHDENLLNSTEFAQPALFAVEVALFRLLGSWGVRPDFVMGHSIGELSA
AHVAGVLSLENAAVLVAARGRLMQALPAGGAMVAVQAAEEEVRPLLSAEVDIAAVNGPASLVISGAQNAVAAVADQLRADGRRVHQLA
VSHAFHSPLMDPMIDEFAAVAAGIAIGRPTIGVISNVTGQLAGDDFGSAAYWRRHIRQAVRFADSVRFAQAAGGSRFLEVGPSGGLVASIEE
SLPDVAVTTMSALRKDRPEPATLTNAVAQGFVTGMDLDWRAVVGEAQFVELPTYAFQRRRFWLSGDGVAADAAGLGLAASELEHHHHH
H*

pIES12

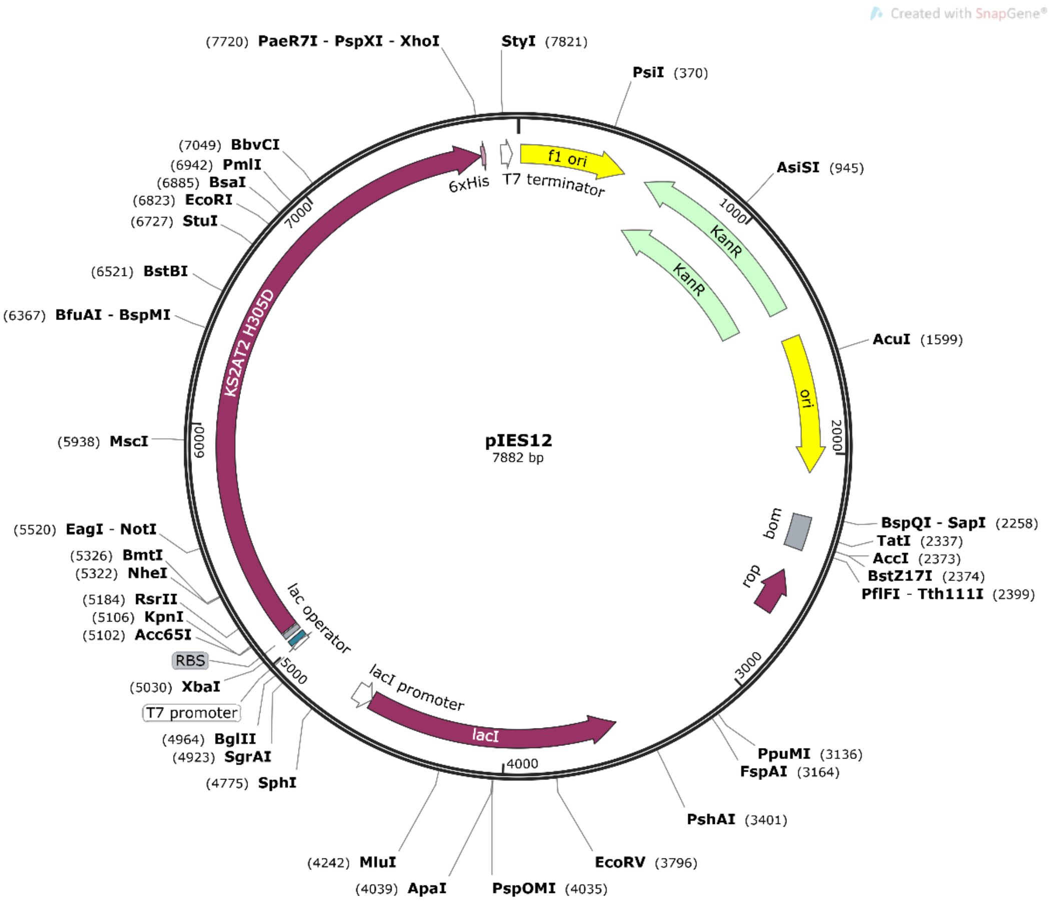

*Mt*PKS12 [KS$_2$][AT$_2$] (H$_{2361}$D) Sequence:

MPIAIVGMACRYPGGVNSPDDMWDMLIQGRDVLSEFPADRGWDLAGLYNPDPDAAGACYTRTGGFVDGVGDFDPAFFGVGPSEALAMD
PQHRMLLELSWEALERAGIDPTGLRGSATGVFAGVMTQGYGMFAAEPVEGFRLTGQLSSVASGRVAYVLGLEGPAVSVDTACSSSLVALH
MAVGSLRSGECDLALAGGVTVNATPDIFVEFSRWRGLSPDGRCKAFAAAADGTGFSEGGGMLVLQRLSDARRLGHPVLAVVVGSAVNQD
GASNGLTAPNGPSQQRVVRAALANAGLSAAEVDVVEG**D**GTGTTLGDPIEAQALLATYGQDRGEPGEPLWLGSVKSNMGHTQAAAGVAG
VIKMVLAMRHELLPATLHVDVPSPHVDWSAGAVELLTAPRVWPAGARTRRAGVSSFGISGTNAHVIIEAVPVVPRREAGWAGPVVPWVVS
AKSESALRGQAARLAAYVRGDDGLDVADVGWSLAGRSVFEHRAVVVGGDRDRLLAGLDELAGDQLGGSVVRGTATAAGKTVFVFPGQG
SQWLGMGMGLHAGYPVFAEAFNTVVGELDRHLLRPLREVMWGHDENLLNSTEFAQPALFAVEVALFRLLGSWGVRPDFVMGHSIGELSA
AHVAGVLSLENAAVLVAARGRLMQALPAGGAMVAVQAAEEEVRPLLSAEVDIAAVNGPASLVISGAQNAVAAVADQLRADGRRVHQLA
VSHAFHSPLMDPMIDEFAAVAAGIAIGRPTIGVISNVTGQLAGDDFGSAAYWRRHIRQAVRFADSVRFAQAAGGSRFLEVGPSGGLVASIEE
SLPDVAVTTMSALRKDRPEPATLTNAVAQGFVTGMDLDWRAVVGEAQFVELPTYAFQRRRFWLSGDGVAADAAGLGLAASELEHHHHH
H*

References

(1) Eng, C. H.; Backman, T. W. H.; Bailey, C. B.; Magnan, C.; Garcia Martin, H.; Katz, L.; Baldi, P.; Keasling, J. D. ClusterCAD: a computational platform for type I modular polyketide synthase design. *Nucleic Acids Res* **2018**, *46* (D1), D509-D515. DOI: 10.1093/nar/gkx893.
(2) Sievers, F.; Wilm, A.; Dineen, D.; Gibson, T. J.; Karplus, K.; Li, W.; Lopez, R.; McWilliam, H.; Remmert, M.; Soding, J.; et al. Fast, scalable generation of high-quality protein multiple sequence alignments using Clustal Omega. *Mol Syst Biol* **2011**, *7*, 539. DOI: 10.1038/msb.2011.75.
(3) Robert, X.; Gouet, P. Deciphering key features in protein structures with the new ENDscript server. *Nucleic Acids Res* **2014**, *42* (Web Server issue), W320-324. DOI: 10.1093/nar/gku316.
(4) Blin, K.; Shaw, S.; Augustijn, H. E.; Reitz, Z. L.; Biermann, F.; Alanjary, M.; Fetter, A.; Terlouw, B. R.; Metcalf, W. W.; Helfrich, E. J. N.; et al. antiSMASH 7.0: new and improved predictions for detection, regulation, chemical structures and visualisation. *Nucleic Acids Res* **2023**, *51* (W1), W46-W50. DOI: 10.1093/nar/gkad344.
(5) Gasteiger, E.; Gattiker, A.; Hoogland, C.; Ivanyi, I.; Appel, R. D.; Bairoch, A. ExPASy: The proteomics server for in-depth protein knowledge and analysis. *Nucleic Acids Res* **2003**, *31* (13), 3784-3788. DOI: 10.1093/nar/gkg563.
(6) Jumper, J.; Evans, R.; Pritzel, A.; Green, T.; Figurnov, M.; Ronneberger, O.; Tunyasuvunakool, K.; Bates, R.; Zidek, A.; Potapenko, A.; et al. Highly accurate protein structure prediction with AlphaFold. *Nature* **2021**, *596* (7873), 583-589. DOI: 10.1038/s41586-021-03819-2.
(7) Mirdita, M.; Schutze, K.; Moriwaki, Y.; Heo, L.; Ovchinnikov, S.; Steinegger, M. ColabFold: making protein folding accessible to all. *Nat Methods* **2022**, *19* (6), 679-682. DOI: 10.1038/s41592-022-01488-1.
(8) Pettersen, E. F.; Goddard, T. D.; Huang, C. C.; Meng, E. C.; Couch, G. S.; Croll, T. I.; Morris, J. H.; Ferrin, T. E. UCSF ChimeraX: Structure visualization for researchers, educators, and developers. *Protein Sci* **2021**, *30* (1), 70-82. DOI: 10.1002/pro.3943.
(9) Datsenko, K. A.; Wanner, B. L. One-step inactivation of chromosomal genes in Escherichia coli K-12 using PCR products. *Proc Natl Acad Sci U S A* **2000**, *97* (12), 6640-6645. DOI: 10.1073/pnas.120163297.
(10) Yu, D.; Ellis, H. M.; Lee, E. C.; Jenkins, N. A.; Copeland, N. G.; Court, D. L. An efficient recombination system for chromosome engineering in Escherichia coli. *Proc Natl Acad Sci U S A* **2000**, *97* (11), 5978-5983. DOI: 10.1073/pnas.100127597.
(11) Doublet, B.; Douard, G.; Targant, H.; Meunier, D.; Madec, J. Y.; Cloeckaert, A. Antibiotic marker modifications of lambda Red and FLP helper plasmids, pKD46 and pCP20, for inactivation of chromosomal genes using PCR products in multidrug-resistant strains. *J Microbiol Methods* **2008**, *75* (2), 359-361. DOI: 10.1016/j.mimet.2008.06.010.
(12) Liu, L. P.; Yang, X.; Zhao, X. J.; Zhang, K. Y.; Li, W. C.; Xie, Y. Y.; Jia, S. R.; Zhong, C. A Lambda Red and FLP/FRT-Mediated Site-Specific Recombination System in Komagataeibacter xylinus and Its Application to Enhance the Productivity of Bacterial Cellulose. *ACS Synth Biol* **2020**, *9* (11), 3171-3180. DOI: 10.1021/acssynbio.0c00450.
(13) Kufs, J. E.; Hoefgen, S.; Rautschek, J.; Bissell, A. U.; Graf, C.; Fiedler, J.; Braga, D.; Regestein, L.; Rosenbaum, M. A.; Thiele, J.; Valiante, V. Rational Design of Flavonoid Production Routes Using Combinatorial and Precursor-Directed Biosynthesis. *ACS Synth Biol* **2020**, *9* (7), 1823-1832. DOI: 10.1021/acssynbio.0c00172.
